# Electrocatalytic Ammonia Oxidation by Pyridyl-Substituted Ferrocenes

**DOI:** 10.1021/jacs.4c14483

**Published:** 2025-02-14

**Authors:** Md Estak Ahmed, Richard J. Staples, Thomas R. Cundari, Timothy H. Warren

**Affiliations:** † Department of Chemistry, Michigan State University, East Lansing, Michigan 48824, United States; ‡ Department of Chemistry, University of North Texas, Denton, Texas 76203, United States

## Abstract

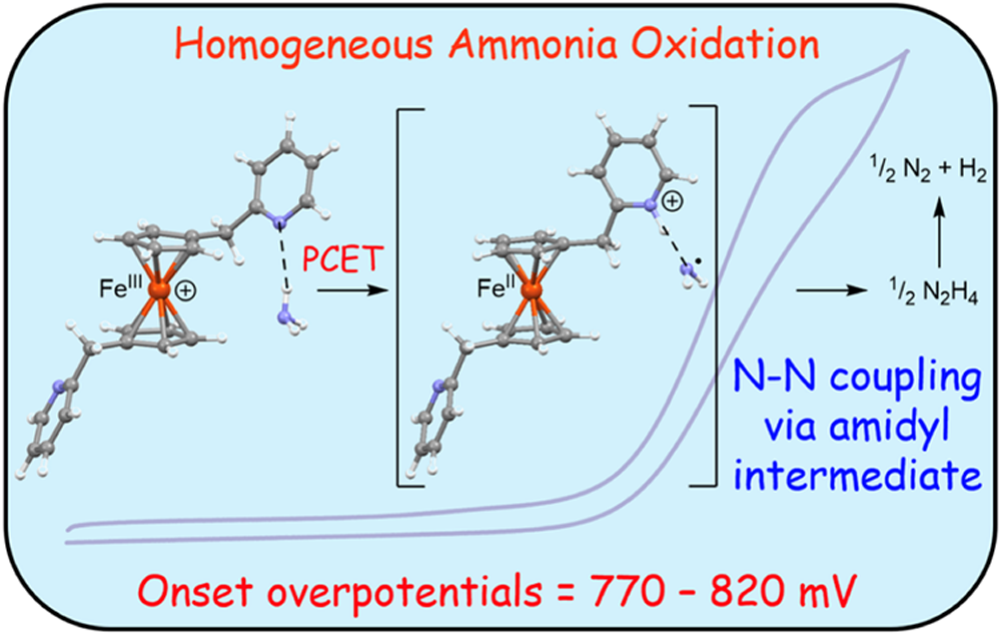

Ammonia (NH_3_) is a promising carbon-free fuel when prepared from sustainable resources. First-row transition metal electrocatalysts for ammonia oxidation are an enabling technology for sustainable energy production. We describe electrocatalytic ammonia oxidation using robust molecular complexes based on Earth-abundant iron. Electrochemical studies of ferrocenes with covalently attached pyridine arms reveal facile ammonia oxidation in DMSO (2.4 M NH_3_) with modest overpotentials (η = 770–820 mV) and turnover frequencies (125–560 h^–1^). Experimental and computational studies indicate that the pendant pyridyl base serves as an H-bond acceptor with an N–H bond of ammonia that transfers a proton to the pyridine following oxidation by the attached ferrocenium moiety in a proton-coupled electron transfer (PCET) step. This generates an amidyl (^•^NH_2_) radical stabilized via H-bonding to a pendant pyridinium moiety that rapidly dimerizes to hydrazine (H_2_N–NH_2_), which is easily oxidized to nitrogen (N_2_) at the glassy carbon working electrode. This report identifies a general strategy to oxidize ammonia via H-bonding to a base (B:), thereby activating [B···H-NH_2_] toward PCET by a proximal oxidant to form [BH···NH_2_]^+/•^ radical cations, which are susceptible to dimerization to form easily oxidized hydrazine.

## Introduction

To address critical energy and climate challenges, sustainable fuels and feedstocks are essential.^1^ As the demand for renewable energy increases, ammonia stands out as an emission-free energy carrier, capable of storing and transporting energy in molecular form.^2^ Ammonia, with an annual global production exceeding 180 million metric tons,^3^ possesses high energy density and efficient distribution networks.^4,5^ Its significant hydrogen storage capacity (17.6% by weight) coupled with low cost and ease of liquefaction make ammonia an attractive medium to store and transport hydrogen for later use.^6^ This is especially true for green ammonia produced through renewable energy resources.^7−9^

**Figure 1:**
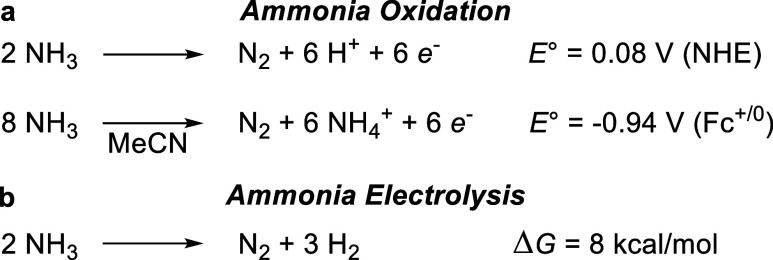
Thermodynamics of ammonia oxidation.

Electrocatalytic ammonia oxidation (AO)^10−13^ converts ammonia to nitrogen gas along with protons and electrons for use in a direct ammonia fuel cell^14,15^ (Figure 1a). Alternatively, ammonia electrolysis (Figure 1b) releases hydrogen stored in ammonia under an applied potential without the need for the high temperatures associated with ammonia cracking.^16,17^ While heterogeneous catalysts enable ammonia oxidation with noble metal catalysts such as Pt,^10,11^ many heterogeneous catalysts based on first-row transition metals such as Ni^10,18^ require large overpotentials over 1.1 V and also exhibit lower catalytic activity for ammonia oxidation.

**Figure 2:**
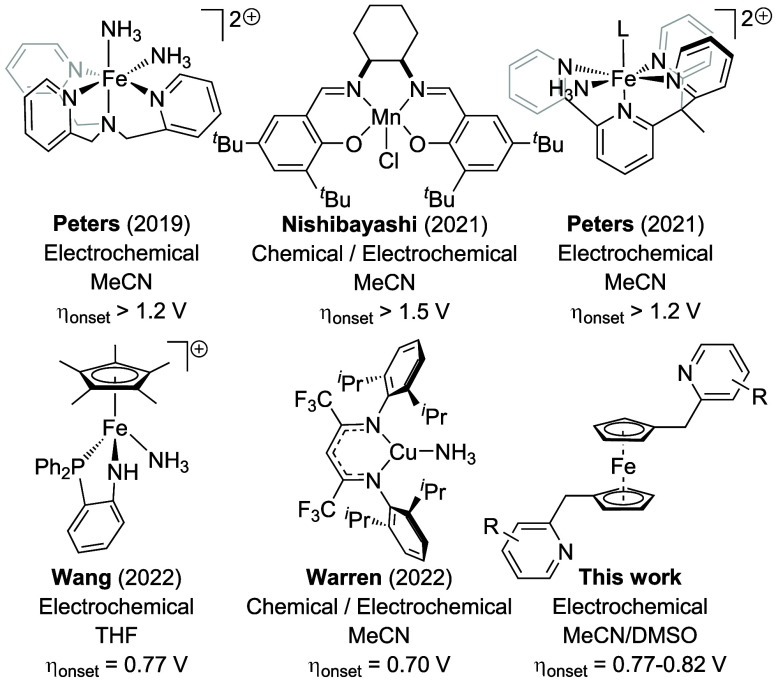
Molecular catalysts based on first-row transition metal elements reported for ammonia oxidation to dinitrogen.

Molecular catalysts for ammonia oxidation offer greater opportunities to uncover mechanistic features that aid in the development of more effective catalysts.^12,13^ Initial reports of molecular catalysts for homogeneous ammonia oxidation rely on expensive, ruthenium-based catalysts,^19−23^ many akin to those utilized in water oxidation catalysis.^24−26^ Catalysts based on first-row transition metals such as Mn,^27^ Fe,^28−31^ and Cu^32,33^ have attracted attention due to sustainability concerns (Figure 2). A persistent challenge for molecular electrocatalysts, however, is their stability in high concentrations of ammonia due to the ready formation of Werner complexes such as [M(NH_3_)_*x*_]^*y*+^ (*x* = 4–6; *y* = 2–3) via ligand displacement.^28,29^

**Figure 3:**
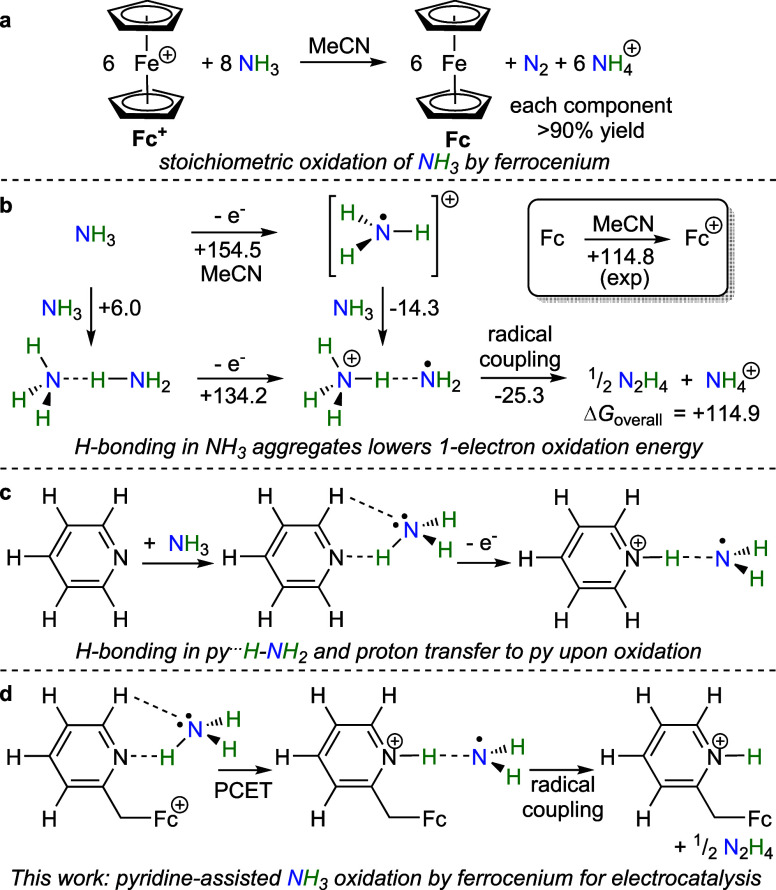
(a) Stoichiometric ammonia oxidation by ferrocenium (Fc^+^). (b) Outer sphere oxidation of monomeric and H-bonded ammonia dimer to hydrazine at B3LYP+GD3BJ/6-311++G(d,p)/SMD-acetonitrile level of theory. Free energies in kcal/mol at 298.15 K (ref (20)). (c) 1-e^–^ oxidation of py···H-NH_2_ to form [pyH···NH_2_]^+/•^. (d) Fc^+^ oxidation of a tethered py···H-NH_2_ species to promote ammonia oxidation.

Recently, our group has shown that ferrocenium (Fc^+^) oxidizes ammonia stoichiometrically to dinitrogen (Figure 3a).^34^ UV–vis kinetics data indicates that ammonia oxidation is first order in [Fc^+^] with a mixed second and third order dependence upon [NH_3_]. Critically, DFT analysis suggests that aggregation of ammonia into H-bonded dimers^35^ or trimers^36^ renders 1-electron oxidation significantly thermodynamically more feasible as compared to an isolated ammonia molecule (Figure 3b).^34^

Seeking to encourage the interaction of ammonia with a base that lowers the potential required for 1-electron oxidation, we note that pyridine forms a H-bonded dimer with ammonia (Figure 3c). Predicted by computational methods^37,38^ and demonstrated in the gas phase by microwave spectroscopy,^37^ the gas phase py···H-NH_2_ binding energy is *ca*. 3 kcal/mol. DFT studies also indicate that 1-electron oxidation of the H-bonded py···H-NH_2_ complex results in movement of a proton from NH_3_ to pyridine, forming a [pyH···NH_2_]^+/•^ radical cation.^37^ The oxidized species is thus an amidyl radical (^•^NH_2_) engaged in H-bonding to a pyridinium cation [pyH]^+^ (Figure 3c). Concomitant proton transfer from ammonia to pyridine upon 1-electron oxidation of py···H-NH_2_ significantly lowers the ionization energy relative to gas-phase ammonia^38^ in this proton-coupled electron transfer (PCET).^39^ This mirrors the dramatic lowering of the potential required for 1-electron oxidation of phenols by 0.5–0.7 V via PCET in H-bonded py···HOAr species relative to free phenols in MeCN solution. In these H-bonded systems, proton transfer to py upon 1-electron oxidation forms [pyH···OAr]^+/•^ radical cations.^40^

These observations led us to consider whether nonoxidizable bases covalently attached to ferrocene might facilitate ammonia oxidation (Figure 3d). Specifically, the formation of a H-bonded py···H-NH_2_ species in the vicinity of an oxidized Fc^+^ center could facilitate electron transfer to reduce Fc^+^ to Fc. The resulting H-bonded amidyl radical formed via PCET would be primed for coupling to form H_2_N-NH_2_, which is much more vulnerable to oxidation than NH_3_.

## Results and Discussion

### Comparing MeCN and DMSO for Ammonia Oxidation by Ferrocene

**Figure 4:**
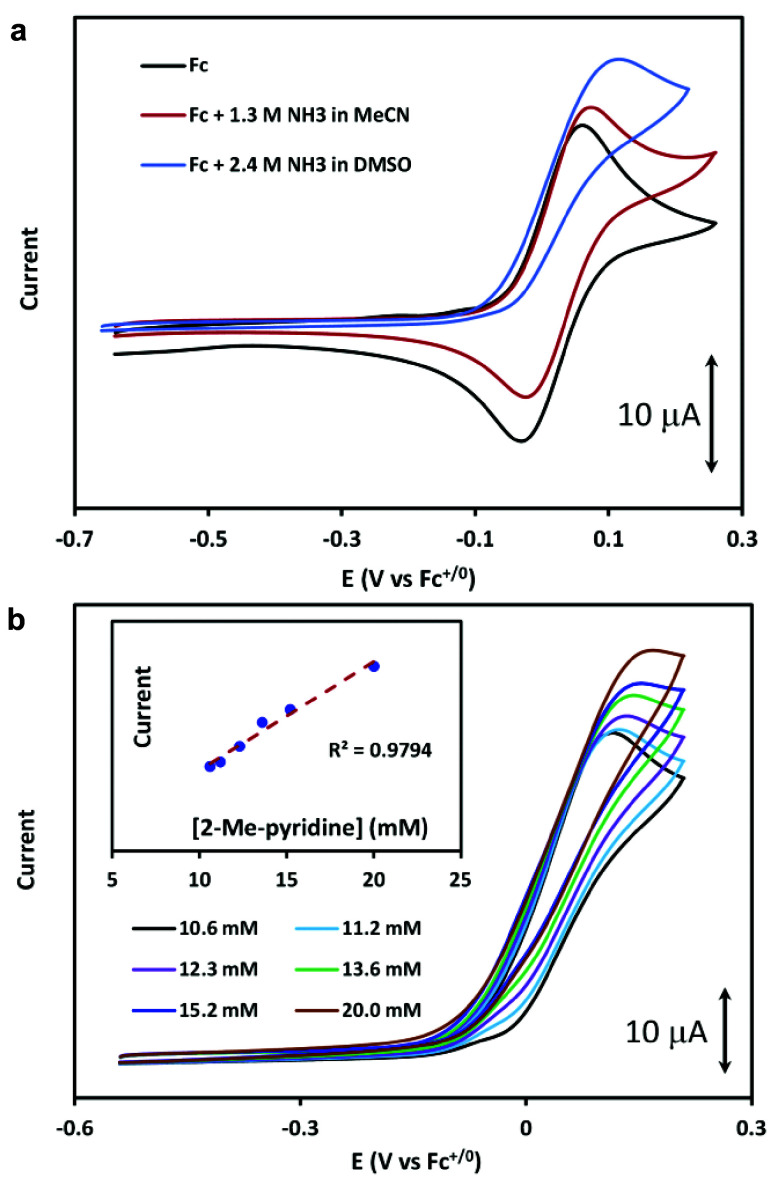
Electrocatalytic ammonia oxidation by ferrocene. (a) Comparison of cyclic voltammograms (CVs) of 1 mM Fc in MeCN without NH_3_ (black), in MeCN (1.3 M NH_3_) (maroon), and DMSO (2.4 M NH_3_) (blue). (b) Cyclic voltammograms of 2.4 mM Fc solution in DMSO (2.4 M NH_3_) with various 2-Mepy concentrations. (inset) Linear peak current vs [2-Mepy]. Conditions: 0.1 M TBAPF_6_ with GC working, Pt counter and Ag/AgNO_3_ reference electrodes; 100 mVs^–1^ scan rate.

One motivation for the molecular modification of ferrocene for ammonia oxidation is that this electrocatalytic process is slow in NH_3_-saturated MeCN with 0.1 M [Bu_4_N]PF_6_ ([NH_3_] = 1.3 M). Analyzed by CV, there is only a very modest increase in oxidation current and decrease in reduction current versus Fc in MeCN without NH_3_ (Figure 4a). Saturating a 0.1 M [Bu_4_N]PF_6_ solution of DMSO with ammonia, however, results in a significantly higher NH_3_ concentration of 2.4 M. This results in a dramatically stronger electrocatalytic response for ammonia oxidation.

### Enhancement of Ammonia Oxidation by Ferrocene with Added Base

We examined the cyclic voltammetry of ferrocene (2.4 mM) in ammonia-saturated DMSO (2.4 M NH_3_) with 2-methylpyridine to explore the hypothesis that a pyridine could activate ammonia toward 1-electron oxidation. Increasing the concentration of 2-methylpyridine (2-Mepy) from 10.6 to 20.0 mM leads to a linear increase in the catalytic current for ammonia oxidation (Figure 4b). This indicates that the external pyridine base enhances the rates of ammonia oxidation with a first order dependence in [2-Mepy].

### Synthesis and Characterization of ^py′2^Fc Complexes

Owing to the rate enhancement in electrocatalytic ammonia oxidation by ferrocene in the presence of 2-methylpyridine, we sought to examine simple ferrocene complexes (η^5^-C_5_H_4_CH_2_(2-py′))_2_Fe (^**py′2**^**Fc**) bearing pendant pyridyl groups py′ tethered by a methylene linker to the cyclopentadienyl rings. This design minimizes the distance of the pendant pyridine to the Fc/Fc^+^ center while ensuring that the attached heteroaromatic ring is not in conjugation with the cyclopentadienyl ring that could increase the reduction potential of the Fc^+^/Fc couple.^41,42^

**Figure 5:**
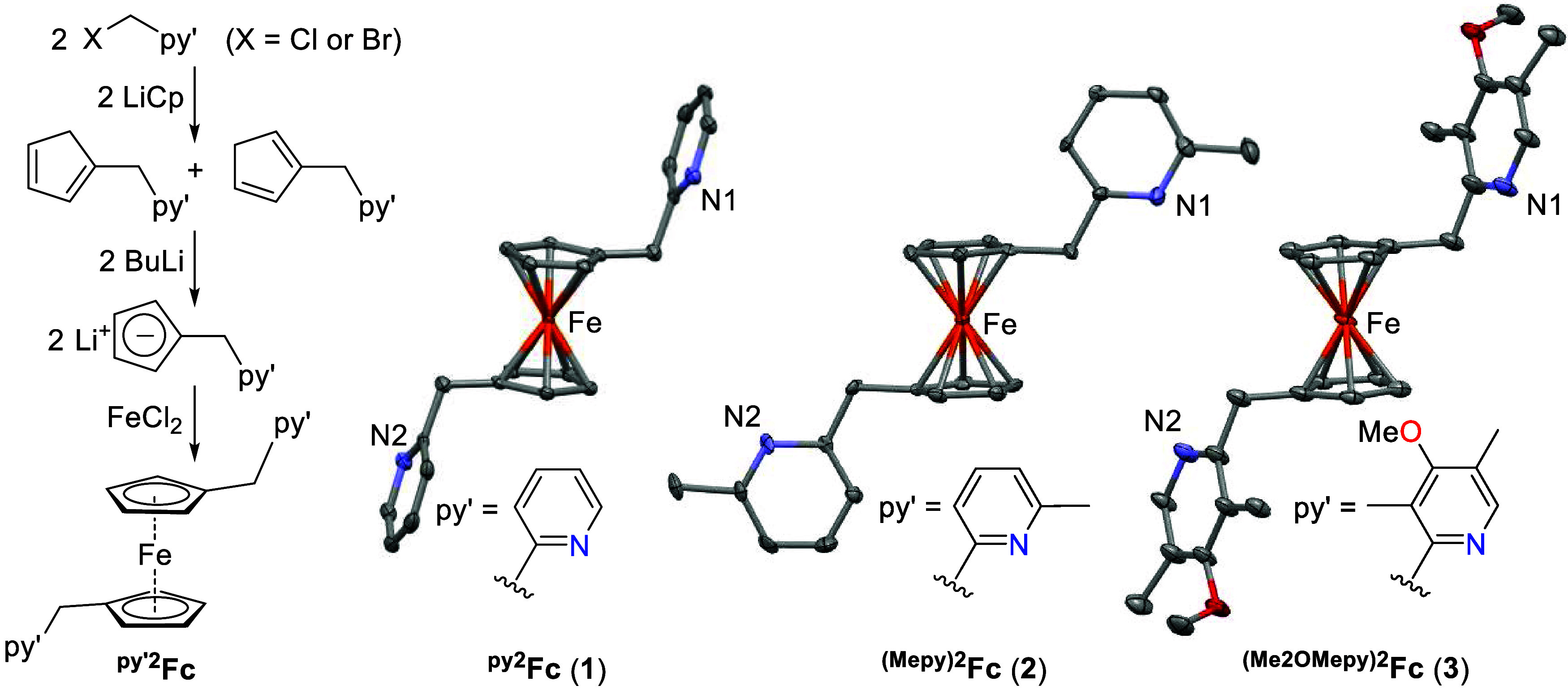
Synthesis of 1,1′-disubstituted methylpyridyl ferrocenes **1**–**3** (left). Single-crystal X-ray structures of complexes **1**–**3** (right). Thermal ellipsoids are shown at 50% probability. All hydrogen atoms are omitted for clarity.

We followed procedures similar to those reported for ^**py2**^**Fc** (**1**) to synthesize complexes (η^5^-C_5_H_4_CH_2_(2-py′))_2_Fe with three different pyridine moieties py′ (py′ = pyridyl (**1**), 6-methylpyridyl (**2**), and 3,5-dimethyl-4-methoxypyridyl (**3**)) (Figure 5).^43,44^ Reaction of the corresponding X-CH_2_py′ (X = Cl or Br) precursors with LiCp provides isomers of the corresponding cyclopentadienes C_5_H_5_CH_2_py′, which after deprotonation by BuLi to Li[C_5_H_4_CH_2_py′] undergoes transmetalation with FeCl_2_ to provide symmetrically 1,1′-substituted metallocenes **1**–**3**.

### Assignment of Overpotential in MeCN for Electrocatalytic Ammonia Oxidation by Ferrocenes **1**–**3**

We briefly examined the cyclic voltammetry (CV) of these 1,1′-2-pyridylmethyl disubstituted ferrocenes in MeCN because the thermodynamic potential for ammonia oxidation in this nonaqueous solvent has been established at −0.94 V vs Fc^+^/Fc.^45^ Utilizing a 0.1 M *n*-tetrabutylammonium hexafluorophosphate (TBAPF_6_) supporting electrolyte and a glassy carbon (GC) working electrode, we observe a reversible redox process at −0.040 V vs Fc^+/0^ (Figure S19a). Saturating the electrolytic solution with NH_3_ results in a 1.3 M NH_3_ solution in MeCN and a substantial increase in the oxidative current, signifying electrocatalytic ammonia oxidation (Figure S19a). The onset potential (deflection point of oxidation wave)^46^ at −120 mV vs Fc^+^/Fc corresponds to an overpotential of 820 mV. Complexes **2** and **3** also exhibit electrocatalytic ammonia oxidation in MeCN with onset potentials of −140 and −170 mV representing overpotentials of 800 and 770 mV, respectively (Figures S19b and S19c).

### Electrocatalytic Ammonia Oxidation by Ferrocenes **1**–**3** in DMSO

**Figure 6:**
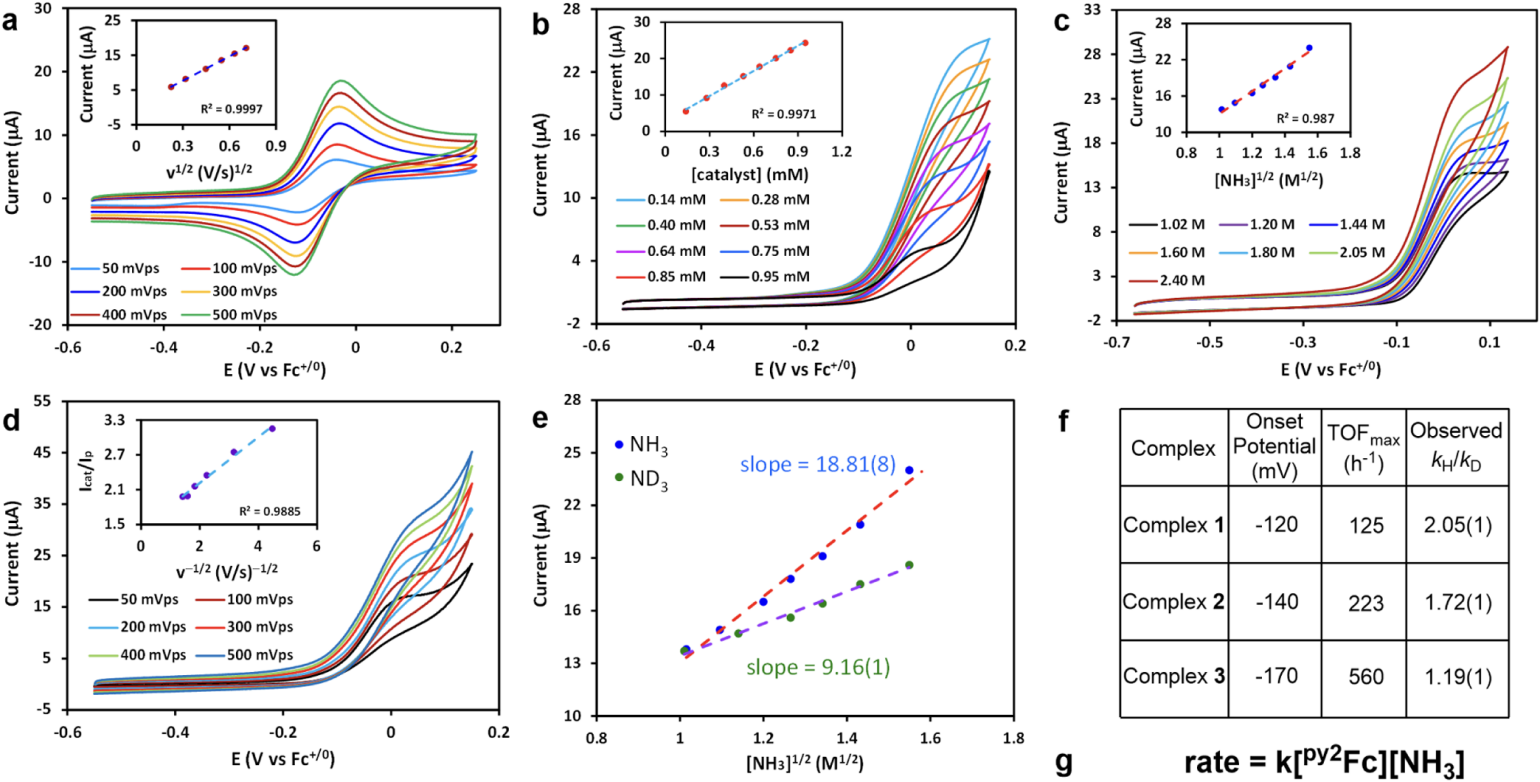
Electrocatalytic ammonia oxidation. (a) Cyclic voltammograms of 1.0 mM ^**py2**^**Fc** solution in DMSO at various scan rates. (b) Cyclic voltammograms of ^**py2**^**Fc** in the presence of various concentration of catalyst in DMSO containing 2.4 M NH_3_. (**c**) Cyclic voltammograms of 1.0 mM ^**py2**^**Fc** solution in DMSO with increasing concentration of ammonia. (d) Cyclic voltammograms of 1.0 mM ^**py2**^**Fc** in DMSO containing 2.4 M NH_3_ at different scan rates. (inset) *I*_cat_/*I*_p_ vs ν^–1/2^ plot. (e) Concentration of NH_3_ or ND_3_ vs catalytic current plot to determine the *k*_H_/*k*_D_ value. (f) Comparison of thermodynamic and kinetic parameters. Onset potential in MeCN and TOF_max_ in DMSO. (g) Rate law of ammonia oxidation. Conditions: 100 mM TBAPF_6_ supporting electrolyte with GC working, Pt counter and Ag/AgNO_3_ reference electrodes.

Due to the increase in the rate of ammonia oxidation by Fc in 2.4 M NH_3_ in DMSO, we more fully evaluated electrocatalytic ammonia oxidation by pyridyl-substituted ferrocenes **1**–**3**. Cyclic voltammetry of complex **1** in DMSO with 0.1 M TBAPF_6_ supporting electrolyte and a GC working electrode shows a reversible redox process at −0.072 V vs Fc^+/0^ (Figure 6a). The peak-to-peak separation of Δ*E* = 76 mV indicates near-Nernstian behavior. The linear variation of peak currents (*I*_p_) with the square root of the scan rates indicates a homogeneous redox couple that following the Randles-Sevcik relationship^47^ enabling the determination of the electrochemical diffusion coefficient of 1.5 × 10^–6^ cm^2^ s^–1^. Likewise, complexes **2** and **3** that bear increasingly electron-rich pyridine pendants show a reversible and quasi-reversible redox process at more anodic potentials, −0.085 V (Δ*E* = 65 mV) and −0.100 V (Δ*E* = 125 mV), respectively (Figures S21 and S22). In both cases the peak currents (*I*_p_) vary linearly with the square root of the scan rates (Figures S21 and S22).

We examined the CV response as a function of the catalyst and ammonia concentration to outline the mechanism for electrocatalysis. Focusing on pyridine-substituted ferrocene ^**py2**^**Fc** (**1**), keeping the ammonia concentration constant ([NH_3_] = 2.4 M) results in a catalytic current (*I*_cat_) that increases linearly with catalyst concentration (0.14–0.95 mM) range (Figure 6b). Thus, the reaction is first order in [catalyst]. Holding the catalyst concentration constant ([^**py2**^**Fc**] = 1.0 mM) and changing the ammonia concentration within the range of 1.02–2.40 M, we found that *I*_cat_ increases as the square root of [NH_3_] (Figure 6c, inset). By analyzing the relationship between *I*_cat_ and [substrate], the electrocatalytic process is also first order in [NH_3_] (Figure 6c). Therefore, we establish the rate law: rate = *k*[^**py2**^**Fc**][NH_3_]. Based on the catalytic response obtained at different scan rates, we determined that at an ammonia concentration of 2.4 M, the TOF_max_ value is 125 h^–1^ (Figure 6d). Related CV analyses of complexes **2** and **3** (Figures S25 and S26) reveal that electrocatalytic ammonia oxidation rates are also first order in [catalyst] as well as [NH_3_]. At 2.4 M NH_3_ concentration, the TOF_max_ is determined to be 223 and 560 h^–1^, respectively for complexes **2** and **3**. Comparison of the TOF values and onset potentials (Figure 6d) reveals a remarkable trend for catalysts **1**–3: *the rate increases with decreasing overpotential*!

Performing ammonia oxidation in varying concentrations of ND_3_, the linear response of *I*_cat_ vs [ND_3_]^1/2^ is shallower than the corresponding response of *I*_cat_ vs [NH_3_]^1/2^, which indicates a primary kinetic isotope effect for each catalyst **1**–3 (Figures 6e, S29c and S30c); the *k*_H_/*k*_D_ values decrease as the pyridine arm becomes more electron-rich with values of 2.05(1), 1.72(1) and 1.19(1), for catalysts **1**, **2**, and **3**, respectively.

### Controlled Potential Electrolysis and Product Analysis

**Figure 7:**
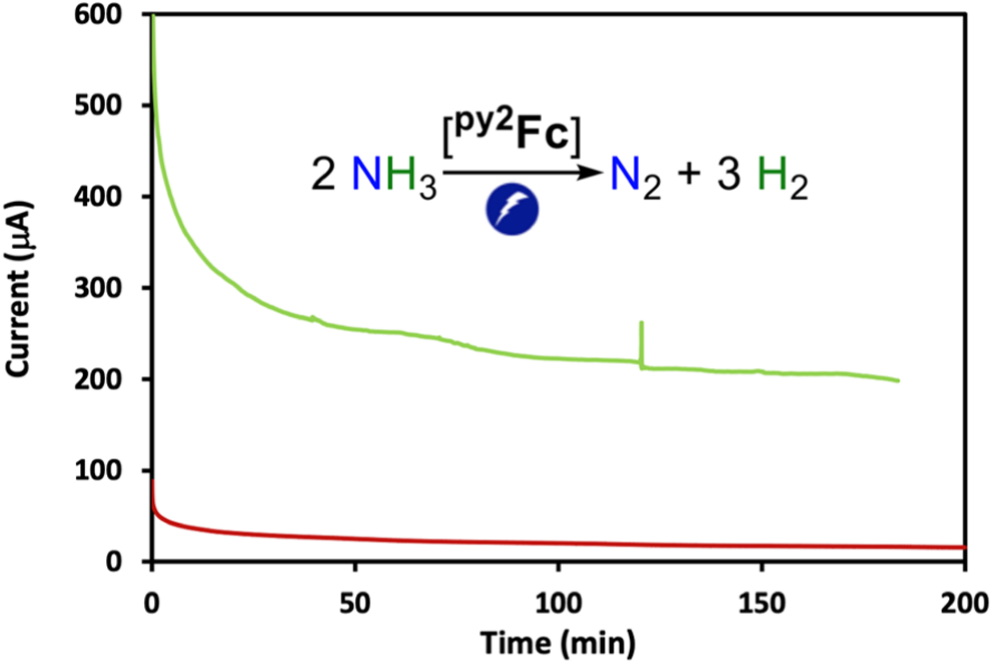
Long-term controlled potential electrolysis of ^**py2**^**Fc** catalyst at 0.1 V vs Fc^+/0^ in 2.4 M NH_3_ in DMSO (1.0 mM ^**py2**^**Fc**, green line; no catalyst, maroon line).

To verify the electrocatalytic nature of the anodic current controlled potential electrolysis (CPE) was performed at 0.1 V vs Fc^+/0^ in DMSO using a GC-plate (1.2 cm^2^), Ag/AgNO_3_, and Pt mesh as working, reference, and counter electrodes, respectively (see SI for detailed experimental setup). Current vs time plots in the CPE of complex **1** showed a substantial increase in charge passed compared to the background electrolyte solution (Figure 7). Headspace gas analysis of the CPE cell by gas chromatography revealed two gaseous products, H_2_ and N_2_, in the molar ratio of 2.74:1 with the corresponding Faradaic yield of 76% for N_2_ evolution. No other gaseous products were detected and analysis of the solution phase did not reveal other nitrogenous products. These results suggest that complex **1** is an electrocatalyst for the oxidation of NH_3_ to N_2_. A long-term CPE experiment (20.2 h) performed in a two-compartment electrochemical cell gave a TON of about 5. A rinse test experiment performed on the glassy carbon electrode after 2 h of CPE in a NH_3_ saturated DMSO solution without the catalyst showed no electrocatalytic current, suggesting the catalytic activity originates from soluble complex **1** (Figure S33). After 3 h of controlled potential electrolysis complexes **2** and **3** also exhibit a similar Faradaic yield for N_2_ evolution as shown in Table S2 (75–80%). The Faradaic yield for H_2_ evolution is 69–72% for complexes **1**–**3** (Table S2). ^1^H NMR analysis of a DMSO-*d*_6_ solution with 2.0 mM **^py2^Fc** (**1**) and 100 mM [Bu_4_N]PF_6_ supporting electrolyte before and after CPE for 2 h reveals no more than 5% loss of catalyst **1** (Figure S32).

### Mechanistic Insights: Ammonia Interaction with ^py2^Fc and Fc^+^

**Scheme 1:**
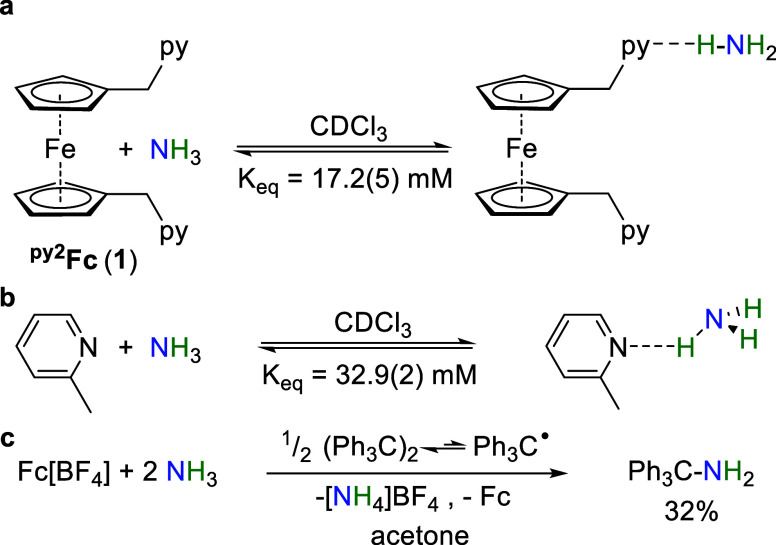
Equilibrium Constants in CDCl_3_ for Ammonia Binding to (a) ^py2^Fc (**1**) and (b) 2-Methylpyridine. (c) Reaction of Gomberg’s Dimer with Fc[BF_4_] in the Presence of Excess NH_3_

We experimentally observe an interaction between the pyridine arm of catalyst **1** and ammonia via ^1^H NMR spectroscopy in CDCl_3_. Both a broadening and upfield shift of the py ^1^H NMR resonances occur as the NH_3_ concentration increases to 130 mM allowing us to estimate the equilibrium constant as 17.2(5) mM^–1^ (Scheme 1a, Figures S16 and S17), commensurate with the equilibrium constant of 32.9(2) mM^–1^ estimated for the association of NH_3_ with the (ferrocene-free) 2-methylpyridine under related conditions (Scheme 1b, Figure S18). Attempts to perform similar analyses of NH_3_ binding in MeCN or DMSO at similar concentrations result in no noticeable NMR shifts, likely reflecting lower py′/NH_3_ binding constants in these significantly more polar solvents.

We sought chemical evidence for generation of the base-stabilized amidyl radical (Figure 3b,c) upon reaction with oxidized catalysts **1**^**+**^–**3**^**+**^ by trapping with the trityl radical Ph_3_C^•^ generated from Gomberg’s dimer. Unfortunately, the oxidized catalysts **1**^**+**^–**3**^**+**^ are not isolable which mirrors the observation that many base-substituted ferrocenes are not stable in their oxidized states.^48^ Therefore, we added Fc[BF_4_] to NH_3_-saturated acetone-*d*_6_ containing 1/2 equiv. Gomberg’s dimer, which resulted in the formation of Ph_3_C-NH_2_ in 32% yield (Scheme 1c). Control experiments indicate that Gomberg’s dimer does not react with NH_3_ in the absence of Fc^+^ to form Ph_3_C-NH_2_ nor does it react with Fc^+^ in the absence of NH_3_.

### Computational Studies: Pyridine-Assisted NH_3_ Oxidation

**Figure 8:**
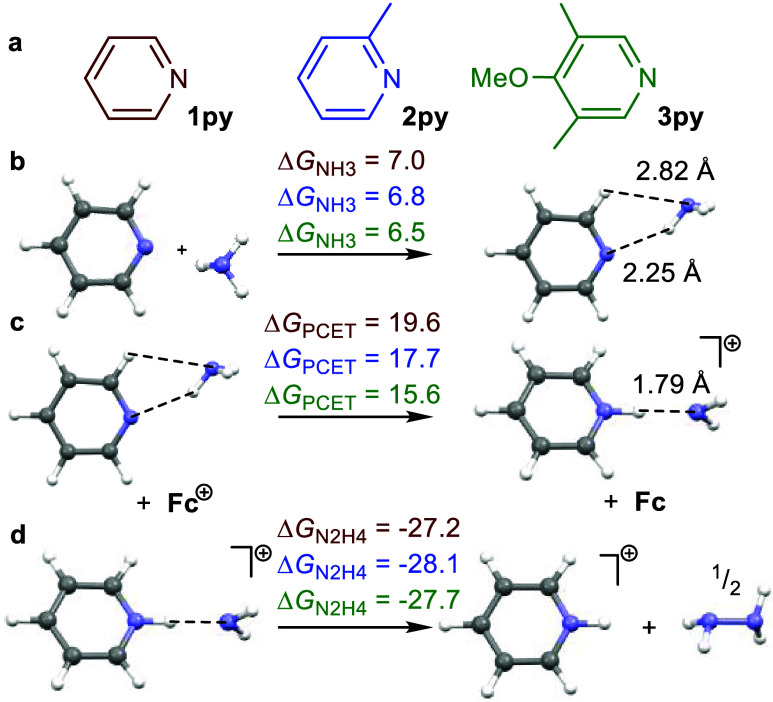
Pyridine-ammonia H-bonding and oxidation in DMSO. (a) Pyridine models. (b) Comparison of Δ*G*_NH3_, Δ*G*_PCET_, and Δ*G*_N2H4_ for various substituted pyridenes. (c, d) Free energies (bold) in kcal/mol at 298.15 K (UB3LYP+GD3BJ/6-311++G(d,p)/SMD-DMSO//B3LYP/6-311+G(d,p)/gas).

To better understand the role of pyridine derivatives in promoting the 1-electron oxidation of ammonia in polar solvents such as DMSO, we employed computational methods at the UB3LYP+GD3BJ/6-311++G(d,p)/SMD-DMSO//B3LYP/6-311+G(d,p)/gas level of theory. Focusing on the pyridine/NH_3_ interaction, we initially examined simple pyridine models for the ferrocene appended pyridyl substituents in catalysts **1**–**3** (Figure 8a).

At this level of theory, the association of NH_3_ with pyridine via H-bonding^37,38^ is endergonic in DMSO (Figure 8b). The attraction is driven by enthalpy (Δ*H* = −1.9 to −2.1 kcal/mol), increasingly favored as the pyridine becomes more electron-rich. Nonetheless, it is uphill in free energy (Δ*G* = 6.5 to 7.0 kcal/mol) due to entropy and further weakened by stabilization of the reactants in the continuum DMSO solvent model.

One-electron oxidation of the py′···H-NH_2_ H-bonded adduct results in proton transfer from ammonia to pyridine to form [py′H···NH_2_]^+/•^ radical cations.^38^ Solvation significantly reduces the energetic cost of 1-electron oxidation as does the py′/NH_3_ interaction. For instance, this level of theory predicts Δ*G* = 233.6 kcal/mol for the gas-phase ionization of NH_3_ (exp = 232.2(5) kcal/mol),^49^ which drops dramatically in DMSO solvent to Δ*G* = 153.8 kcal/mol. The py′···H-NH_2_ interaction further lowers the 1-electron oxidation energy to 135.6 to 131.6 kcal/mol for pyridines **1py −3py**, and is the lowest for the electron-rich 3,5-dimethyl-4-methoxypyridine (**3py**). Scaling these energies against the calculated Fc/Fc^+^ couple in DMSO (116.0 kcal/mol (DFT)) reveals that the free energy corresponding to PCET that forms [py′H···NH_2_]^+/•^ upon oxidation of py′···H-NH_2_ by Fc^+^ drops from 19.6 to 15.6 kcal/mol as the pyridine becomes more electron-rich (Figure 8c).

Critically, DFT calculations predict the [py′H···NH_2_]^+/•^ radical cations to be unstable toward N–N coupling to form H_2_N-NH_2_ by 27–28 kcal/mol (Figure 8d). These [py′H···NH_2_]^+/•^ species are pyridinium (py′H^+^) stabilized amidyl radicals with essentially all of the unpaired electron density on the amidyl N atom (Figures S46, S51, and S56). Thus, irreversible N–N coupling could provide a driving force to counteract uphill PCET promoted by Fc^+^ to convert the H-bonded ammonia adduct py′···H-NH_2_ to the radical cation [py′H···NH_2_]^+/•^. The overall thermodynamic favorability of ammonia oxidation by Fc^+^ promoted by pyridines **1py**–**3py** increases from 0.6 to 5.6 kcal/mol in free energy (Table S11), becoming more favorable as the pyridine becomes more electron-rich.

### Computational Studies: Ammonia Oxidation by Pyridine-Substituted Ferrocenes 1–3

**Figure 9:**
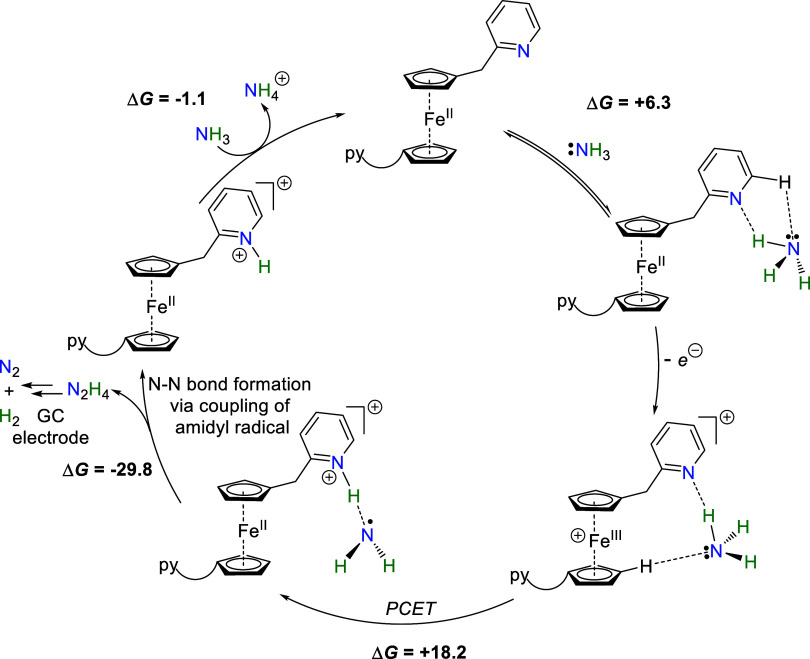
Proposed catalytic cycle for electrocatalytic NH_3_ oxidation by catalyst **1**. Free energies (bold) in kcal/mol at energies (bold) in kcal/mol at 298.15 K.

**Figure 10:**
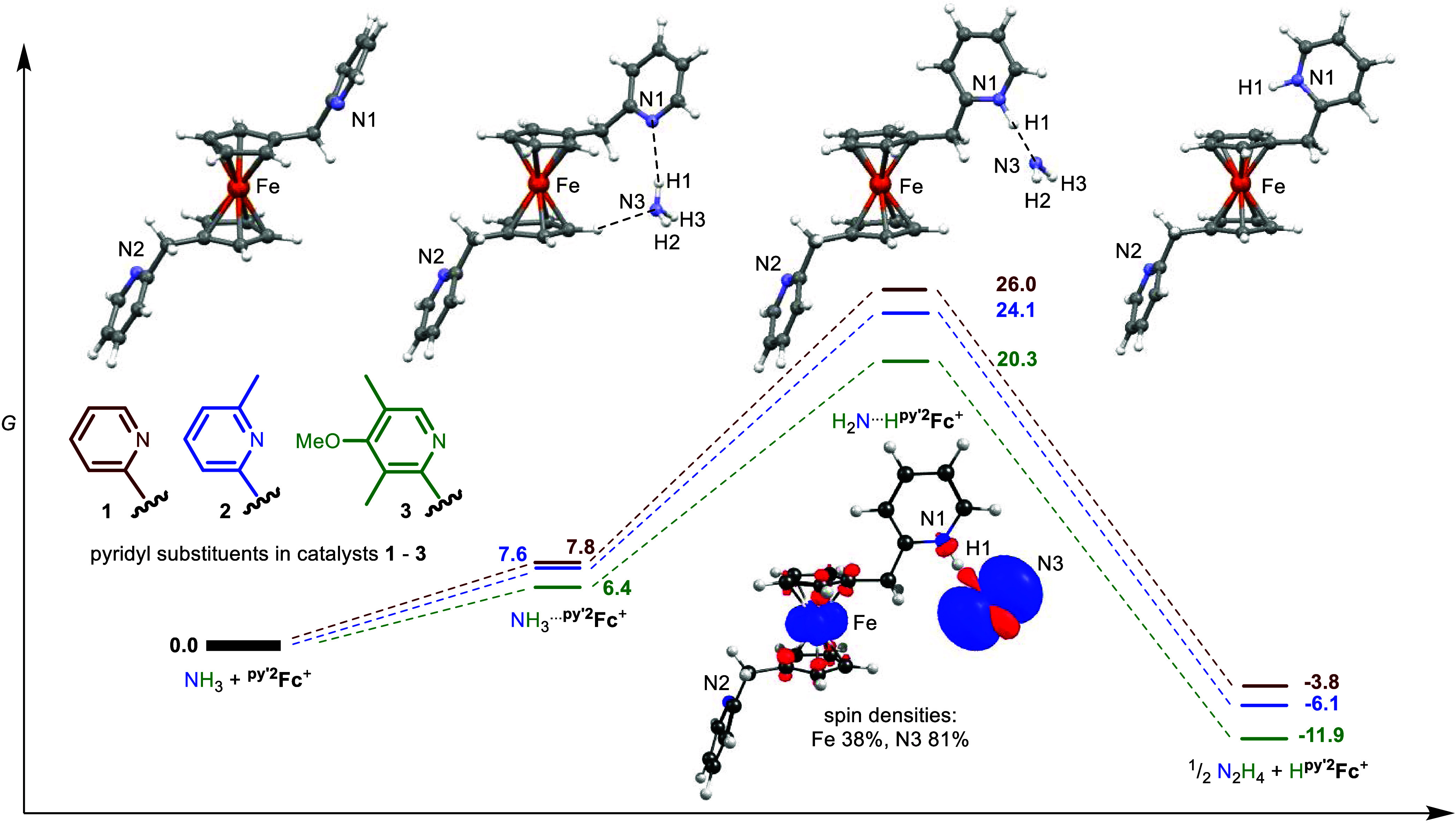
Reaction coordinate diagram for NH_3_ oxidation by ^**py′2**^**Fc**^**+**^ species in DMSO. Different colors correspond to the differently substituted ferrocene complexes ^**py2**^**Fc** (**1**, maroon), ^**(Mepy)2**^**Fc** (**2**, blue), and ^**(Me2OMepy)2**^**Fc** (**3**, green). DFT structures of intermediates shown based on ^**py2**^**Fc**^**+**^ (**1**^**+**^), including a spin density plot of the PCET intermediate {H_2_N···H^py′2^Fc}^+^. Free energies (bold) in kcal/mol at 298.15 K (UB3LYP+GD3BJ/6-311++G(d,p)/SMD-DMSO//B3LYP/6-311+G(d,p)/gas).

Trends in ammonia binding and electron-transfer energies revealed from detailed consideration of py′···H-NH_2_ species (Figure 8) extend to full models of the pyridine-substituted ferrocenes **1**–**3** that serve as electrocatalysts for ammonia oxidation (Figures 9 and 10; Tables S5–S9). NH_3_ association to the neutral ^**py′2**^**Fc** (3.8–6.3 kcal/mol; Table S5) and cationic models ^**py′2**^**Fc**^**+**^ models (6.4–7.8 kcal/mol; Table S6) is modestly uphill in free energy, and favored by increasing the electron-richness of the pendant pyridine. Interestingly, the lowest energy NH_3_ binding mode to cationic ^**py′2**^**Fc**^**+**^ complexes involves ammonia serving as an H-bond donor to the pendant pyridine and an H-bond acceptor from a cyclopentadienyl C–H bond (Figures 10, S60, S69 and S78). The dual H-bonding interaction between ^**py′2**^**Fc**^**+**^ cations **1**^**+**^–**3**^**+**^ and NH_3_ places the ammonia close to the Fe center (Fe···N = 4.49–4.93 Å), facilitating electron-transfer from ammonia to the oxidized Fc^+^ fragment. While the initial binding of NH_3_ can occur to either the reduced ^py^′^2^Fc or oxidized ^py^′^2^Fc^+^ forms, for simplicity we illustrate NH_3_ binding to reduced ^py^′^2^Fc in the catalytic cycle that appears in Figure 9. This also reflects that the catalysts rest in their reduced state in solution away from the electrode.

Proton transfer to the pendant pyridine in {H_2_NH···^**py′2**^**Fc**}^+^ species accompanies uphill electron transfer from the H-bonded ammonia to the ferrocenium moiety. Performing a relaxed energy scan on the ammonia-bound {H_2_NH···^**py′2**^**Fc**}^+^ species that lengthens the ammonia H_2_N···H distance indicates that the H···Npy′ distance shortens to become a pyridinium fragment (Hpy′^+^) bound to an amidyl radical (^•^NH_2_) in {H_2_N···H^**py′2**^**Fc**}^+^. This signals electron transfer from bound NH_3_ to give a reduced ferrocene fragment (Figures 10 (inset) and S61, S70, and S79). By constraining the pyN-H distance, optimized structures for these intramolecular electron transfer products {H_2_N···H^**py′2**^**Fc**}^+^ allow the estimation of the thermodynamic barriers for PCET. The energies required for PCET drop from 18.2 to 13.9 kcal/mol as the pendant pyridine becomes more electron-rich. Highly favorable, irreversible bimolecular N–N coupling of amidyl radicals in {H_2_N···H^**py′2**^**Fc**}^+^ species to H_2_N-NH_2_ (Δ*G* = −29.8 to −32.2 kcal/mol) drives uphill PCET. Overall oxidation of ammonia by ^**py′2**^**Fc**^**+**^ to hydrazine and ^H**py′2**^**Fc**^**+**^ is 3.8–11.9 kcal/mol downhill, and most favored by the electron-rich pyridine pendant in catalyst **3**. Deprotonation of the pyridinium pendant by excess NH_3_ returns catalysts **1**–3 (Table S9).

### Isotope Effects in NH_3_/ND_3_ Binding to Pyridines

While PCET reactions typically give rise to primary kinetic isotope effects,^50,51^ the KIEs observed in the electrocatalytic ammonia oxidation range from 2.05(1) to 1.19(1) (Figure 6f). We rationalize these as net KIEs that come from the competition between normal KIEs (KIE > 1) for the PCET step^50,52^ and an inverse equilibrium isotope effect (EIE < 1) for the preceding ammonia binding step (Figure 8b). For instance, H-bonding typically results in a preference of D over H in the bridging position.^53^ Using the simple pyridine models **1py**–**3py**, our DFT studies predict EIE’s of 0.94 to 0.91 that decrease as the pyridine arm becomes more electron-rich (Table S10). In the combination of ammonia binding equilibria (Figure 8b) and PCET (Figure 8c) steps, the lower EIE for ammonia binding with the electron-rich pyridine **3py** rationalizes the decreasing observed KIEs in ammonia oxidation by pyridine-substituted ferrocenes **1**–**3**.

## Conclusions

This work introduces robust, molecular electrocatalysts **1**–**3** for ammonia oxidation based on ferrocene with the earth-abundant metal iron at its center. Rather than featuring intimate metal-ammonia interactions as in previous molecular electrocatalysts, ammonia binding to a pendant pyridine arm represents the key enabling feature. Hydrogen bonding between a pyridine pendant and ammonia close to an oxidized ferrocene center promotes PCET to form the ferrocene linked [pyH···NH_2_]^+/•^ radical cation susceptible to N–N coupling to form hydrazine. While we do not detect hydrazine, it readily undergoes oxidation at GC electrodes at significantly lower potentials (−0.4 V vs Fc^+^/Fc)^32^ than the Fc-based catalysts **1**–3. Moreover, electrocatalytic AO rates increase with decreasing onset overpotentials for catalysts **1**–3 in 2.4 M NH_3_ DMSO, a solvent that enables higher ammonia concentrations than THF (0.34 M)^19^ or MeCN (1.3 M).^32^

A critical feature of this electrocatalyst system is the H-bonding interaction between ammonia and the pendant pyridine arm. Through H-bonding to the nonoxidizable base, the ammonia molecule becomes more electron-rich, significantly lowering the potential required for oxidation. Not only do more electron-rich pyridines enhance the H-bonding interaction, but they also stabilize the [pyH···NH_2_]^+/•^ radical cation generated upon 1-electron oxidation of ammonia. Moreover, the [pyH···NH_2_]^+/•^ radical cation possesses essentially all of its radical character at the amidyl N atom, priming it for bimolecular N–N coupling to H_2_N-NH_2_.

The pendant pyridine arm in catalysts **1**–3 ensures that ammonia and ferrocene center remain in close contact. This proximity (Fe···N: 4.49–4.93 Å) facilitates electron transfer from the pyridine-activated ammonia to the oxidized ferrocene. Proton transfer accompanies electron transfer (PCET) to form an amidyl radical H-bonded a pyridinium pendant arm on the reduced ferrocene center. Importantly, highly favorable, irreversible N–N coupling of the amidyl radicals to hydrazine drives the uphill PCET reaction.

A combination of experimental and computational observations suggest that the PCET step is turnover limiting for molecular AO catalysts **1**–3. Since increasing the electron-richness of the pendant pyridine favors both the H-bonding and PCET steps as well as modestly lowers the potential of the ^**py2**^′**Fc**^**+**^/^**py′2**^**Fc** redox couple, we observe increasing rates for electrocatalytic AO with decreasing onset overpotentials. While catalysts **1**–3 possess two pendant pyridine arms due to their ease of synthesis (Figure 5), we note that only one pendant pyridine is required. Electrochemical assessment of (η^5^-C_5_H_4_CH_2_(2-py))(η^5^-Cp)Fe (^**py**^**Fc**; **4**) reveals a similar TOF_max_ (117 h^–1^) and onset potential (−100 mV) as compared to catalyst **1** with two pyridine arms (TOF_max_ = 125 h^–1^; onset potential = −120 mV) (Figure S27; Table S1).

This study introduces H-bonding between ammonia and redox stable bases as a design element to enable ammonia oxidation at modest potentials. Ferrocene serves as a robust redox mediator^54,55^ whose effectiveness is enhanced by covalently attaching pyridine arms for ammonia activation. These studies suggest that highly electron-rich, nonoxidizable bases coupled with chemically stable redox mediators could enable ammonia oxidation at lower potentials controlled by the electronic features of the redox mediator.

## Supporting Information Available

Experimental details; materials and methods; UV–vis, X-ray structure details, computational details (PDF)
