# Supplementary material for: Electrocatalytic Ammonia Oxidation by Pyridyl-Substituted Ferrocenes
Source: J Am Chem Soc. 2025 Feb 14;147(8):6514–22. doi: 10.1021/jacs.4c14483 (PMC11869277; doi:10.1021/jacs.4c14483)
Supplement: Supplementary file 1 — ja4c14483_si_001.pdf [file ja4c14483_si_001.pdf]

Supporting Information

Electrocatalytic Ammonia Oxidation by Pyridyl Substituted Ferrocenes

Md Estak Ahmed,† Richard J. Staples,† Thomas R. Cundari,‡ and Timothy H. Warren†,*

†Department of Chemistry, Michigan State University, East Lansing, MI 48824, United States

‡Department of Chemistry and CASCaM, University of North Texas, Denton, Texas 76203, United States

*Corresponding author email: warre155@msu.edu

Table of Contents

1. General Procedures **S2**
2. Materials **S2**
3. Synthesis of Pyridyl Substituted Ferrocene complexes (**1** – **4**) **S3**
   3.1. Ligand Synthesis **S3**
   3.2. Synthesis of Ferrocenes with Pendant Pyridine Arms **S9**
4. Reaction of Gomberg's Dimer with •NH_2 Intermediate **S17**
5. Determination of the Equilibrium Binding Constant Between Ammonia and 2-Me-pyridine and Pyridine-substituted Ferrocene **S18**
6. Electrochemistry **S21**
7. Determination of Overpotential of Ammonia Oxidation in MeCN Solvent **S22**
8. Determination of Diffusion Coefficient of Substituted Ferrocene Complexes by Cyclic Voltammetry in DMSO **S23**
9. Rate and Mechanistic Information of Ferrocene Complexes from Cyclic Voltammetry Experiments **S25**
10. Controlled Potential Electrolysis (CPE) **S33**
11. Rinse Test **S34**
12. Gas Chromatography (GC) to Detect N_2 and H_2 **S35**
13. Faradaic Yield (FY) Calculation form CPE Experiments **S36**
14. Determination of Turnover Number (TON) for Complex **1** **S38**
15. Crystallographic Details **S39**
16. Computational Results **S44**
17. References **S73**

1. General Procedures

Synthesis was conducted in a dry nitrogen atmosphere using MBraun gloveboxes and/or standard Schlenk techniques unless specified otherwise. ^{1}H and ^{13}C{^{1}H} NMR spectra were recorded on a Varian 500 MHz spectrometer at room temperature. For ^{1}H NMR spectra the residual signal of deuterated solvent serves as an internal standard. The chemical shift (δ) values are expressed in ppm relative to tetramethylsilane. A PerkinElmer 2400 Series II CHNS/O analyzer was used for CHN elemental analyses. UV−vis spectra were recorded on Agilent 8454 Diode Array spectrometer equipped with stirrer and Unisoku USP−203 cryostat for variable temperature (−105 °C to 90 °C) experiments.

2. Materials

All chemicals were purchased from common vendors (e.g., Sigma-Aldrich, Acros Organics, Strem Chemicals, TCI, Fisher Scientific) and used without further purification unless otherwise mentioned. Molecular sieves (4A, 4-8 mesh beads) were obtained from Fisher Scientific and activated *in vacuo* at 250 °C for 48 h. Extra dry solvents (≥ 99.5%) with Acroseal® and deuterated solvents were purchased from Acros Organics and Cambridge Isotope Laboratories, respectively. Both anhydrous and deuterated solvents were sparged with nitrogen and stored over activated 4A molecular sieves under a nitrogen atmosphere. 2-(chloromethyl)pyridine hydrochloride, 2-(bromomethyl)-6-methylpyridine, 2-chloromethyl-4-methoxy-3,5-dimethylpyridine hydrochloride, and ^{n}BuLi (2.5 M in hexane) were purchased from Sigma-Aldrich and used without further purification. Lithium cyclopentadienide was purchased from Fisher Scientific and used as received. $FeCl_2$ was purchased from Strem and used as received. Anhydrous NH_3 was purchased from Airgas. ND_3 cylinders were purchased from Sigma-Aldrich. $FeCl_2$•$(THF)_{1.5}$ was prepared following the literature procedure.[1] Gomberg's dimer was synthesized following a literature procedure.[2]

3. Synthesis of Pyridyl Substituted Ferrocene complexes (1 – 4)

3.1. Ligand Synthesis

3.1.1. Synthesis of 2-(cyclopenta-1,3-dien-1-ylmethyl)pyridine and 2-(cyclopenta-1,4-dien-1-ylmethyl)pyridine (1:0.8 mixture)

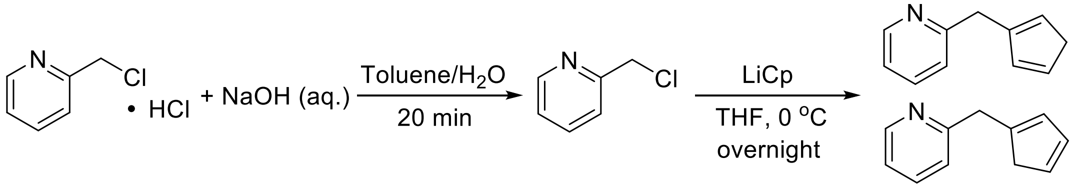

The compound was synthesized by a slight modification of the previously reported procedure.[3] To a solution of NaOH in water (2.25 g, 56.25 mmol, 1.37 equiv.), 2-picolyl chloride hydrochloride (6.74 g, 41.09 mmol, 1.0 equiv.) was added. The resulting mixture was then extracted three times with toluene (20 mL) and the combined organic layers were dried with $MgSO_4$. This mixture was slowly added dropwise to a solution of LiCp (3.24 g, 45.25 mmol, 1.10 equiv.) in 10 mL THF at 0 °C. During the addition, an off-white precipitate formed and the reaction mixture turned brown. After allowing the mixture to stir overnight, an excess of H_2O was added and the layers were separated. The organic layer was then dried with $MgSO_4$ and the solvent was removed to obtain a dark, golden oil. Flash column chromatography was performed to purify the product (basic aluminum oxide, pentane/diethyl ether = 1/4) to give clear colorless oil. The resulting product was a mixture of 2-(cyclopenta-1,3-dien-1-ylmethyl)pyridine and 2-(cyclopenta-1,4-dien-1-ylmethyl)pyridine (1:0.8 mixture). The product was highly sensitive to air, and the color of the pure product changed to pink within a few min of exposure to air at room temperature. To avoid the Diels-Alder dimerization reaction we immediately used the synthesized ligand for the next steps. The isolated yield was 43%. ^{1}H NMR (500 MHz, $CDCl_3$, 298 K): δ (ppm) 8.43 (m, 2H), 7.44 (m, 2H), 7.03 (m, 2H), 6.96 (m, 2H), 6.35–5.98 (m, 6H), 3.82, 3.80 (2 × d, 4H), 2.87, 2.80 (2 × m, 4H). $^{13}C\{^{1}H\}$ NMR (125 MHz, $CDCl_3$, 298 K): δ (ppm) 160.61, 160.15, 149.23, 149.20, 146.29, 144.11, 136.34, 136.28, 134.32, 134.05, 132.28, 131.66, 128.60, 128.16, 122.89, 122.84, 121.07, 121.05, 43.19, 41.31, 39.98, 39.07. HRMS (ESI+) for $C_{11}H_{12}N^{+}$ $(M + H)^{+}$: calcd. 158.1010, found 158.0983.

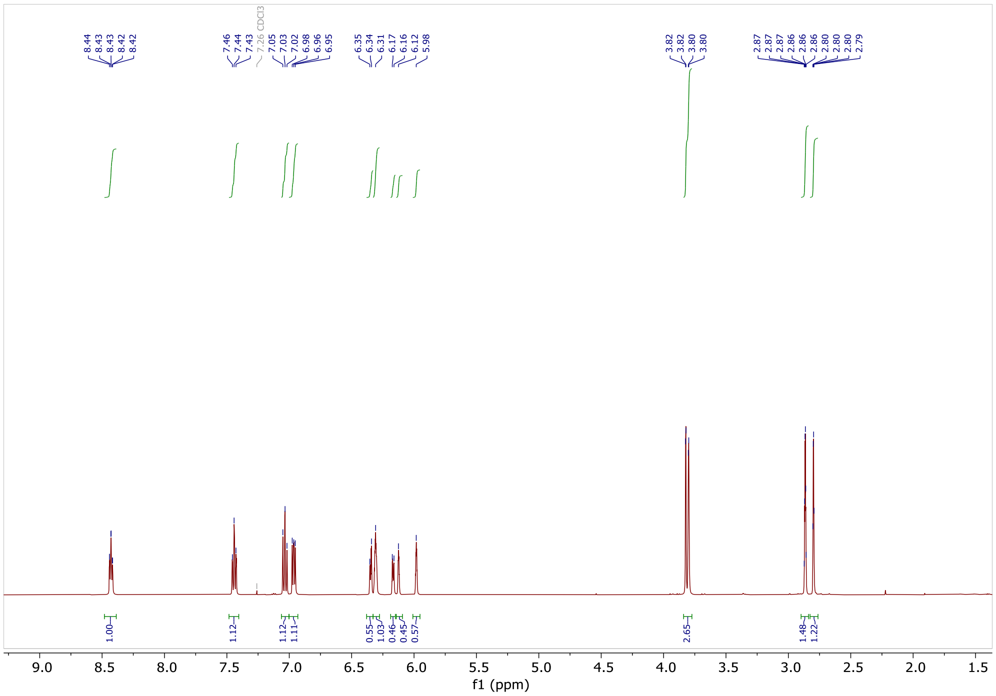

Figure S1. ^{1}H NMR spectrum (500 MHz, $CDCl_3$, 22 °C) of 2-(cyclopenta-1,3-dien-1-ylmethyl)pyridine and 2-(cyclopenta-1,4-dien-1-ylmethyl)pyridine isomers.

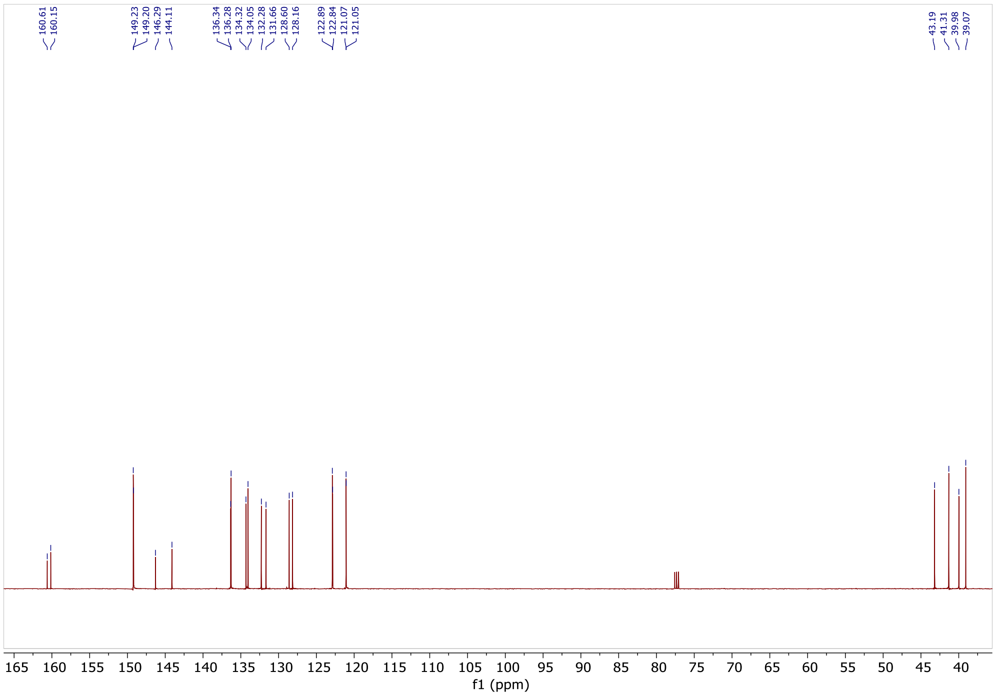

Figure S2. $^{13}C\{^{1}H\}$ NMR spectrum (125 MHz, $CDCl_3$, 22 °C) of 2-(cyclopenta-1,3-dien-1-ylmethyl)pyridine and 2-(cyclopenta-1,4-dien-1-ylmethyl)pyridine isomers.

3.1.2. Synthesis of 2-(cyclopenta-1,3-dien-1-ylmethyl)-6-methylpyridine and 2-(cyclopenta-1,4-dien-1- ylmethyl)-2-methylpyridine (1:1 mixture)

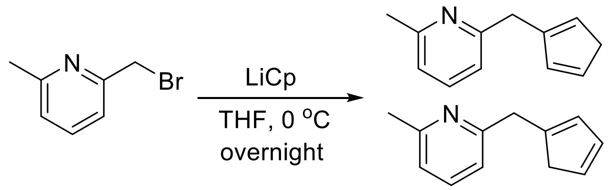

The compound was synthesized by a slight modification of the previously reported procedure.[3] 2-(bromomethyl)-6-methylpyridine (3.24 g, 45.25 mmol, 1.0 equiv.) was dissolved in 15 mL THF and cooled to –40 °C in the glovebox. The solution was slowly added to a cold solution of LiCp (3.24 g, 45.25 mmol, 1.10 equiv.) in 20 mL THF at –40 °C. During the addition, an off-white precipitate formed, and the reaction mixture turned brown. After allowing the mixture to stir overnight, an NH_4Cl saturated excess H_2O was added, and the layers were separated. The organic layer was then dried with $MgSO_4$, and the solvent was removed to obtain a dark, golden oil. Flash column chromatography was performed to purify the product (basic aluminum oxide, pentane/diethyl ether = 1/4) to give clear colorless oil. The resulting product was a mixture of 2-(cyclopenta-1,3-dien-1-ylmethyl)-2-methylpyridine and 2-(cyclopenta-1,4-dien-1-ylmethyl)-2-methylpyridine (1:1 mixture). The product was highly sensitive to air, and the color of the pure product changed to pink within a few min of exposure to air at room temperature. To avoid the Diels-Alder dimerization reaction we immediately used the synthesized ligand for the next steps. The isolated yield was 42%. ^{1}H NMR (500 MHz, $CDCl_3$, 298 K): δ (ppm) 7.42 (m, 2H), 6.91 (m, 4H), 6.43–6.02 (m, 6H), 3.87, 3.84 (2 × d, 4H), 2.93, 2.88 (2 × m, 4H), 2.51 (2 × s, 6H). $^{13}C\{^1H\}$ NMR (125 MHz, $CDCl_3$, 298 K): δ (ppm) 160.00, 159.54, 157.72, 146.60, 144.30, 136.62, 136.58, 134.54, 133.97, 132.32, 131.65, 128.55, 128.18, 120.62, 120.59, 119.79, 43.34, 41.34, 40.06, 39.15, 24.54, 24.53. HRMS (ESI+) for $C_{12}H_{14}N^+$ (M + H)$^+$: calcd. 172.1135, found 172.1127.

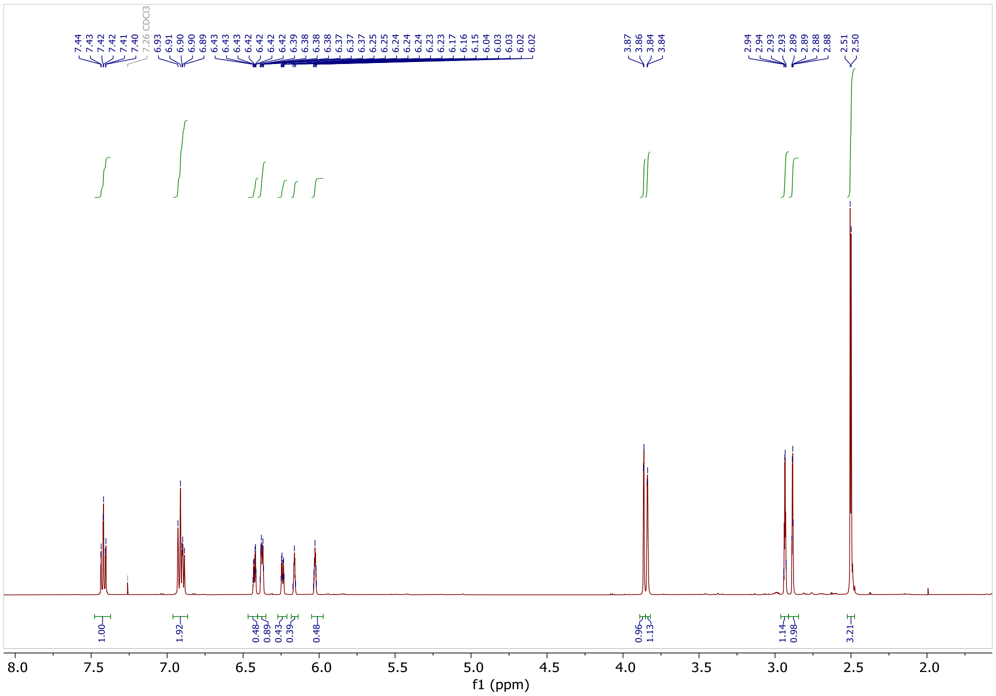

Figure S3. ^{1}H NMR spectrum (500 MHz, $CDCl_3$, 22 °C) of 2-(cyclopenta-1,3-dien-1-ylmethyl)-6-methylpyridine and 2-(cyclopenta-1,4-dien-1-ylmethyl)-2-methylpyridine isomers.

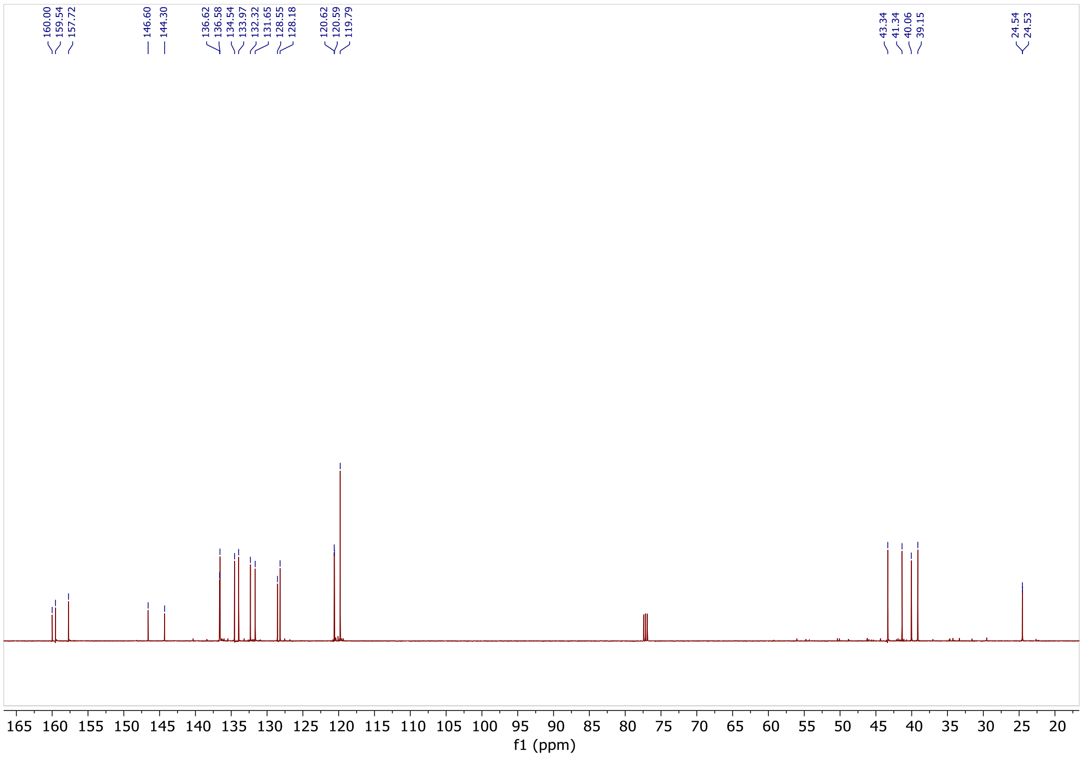

Figure S4. $^{13}C\{^{1}H\}$ NMR spectrum (125 MHz, $CDCl_3$, 22 °C) of 2-(cyclopenta-1,3-dien-1-ylmethyl)-6-methylpyridine and 2-(cyclopenta-1,4-dien-1-ylmethyl)-2-methylpyridine isomers.

3.1.3. Synthesis of 2-(cyclopenta-1,3-dien-1-ylmethyl)-4-methoxy-3,5-dimethylpyridine and 2-(cyclopenta-1,4-dien-1-ylmethyl)-4-methoxy-3,5-dimethylpyridine (1:1 mixture)

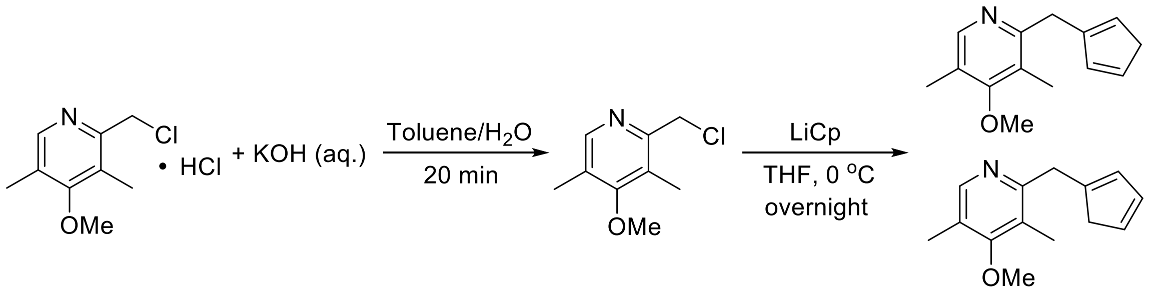

This new compound was synthesized by a slight modification of the previously reported procedure.[3] To a solution of KOH in water (2.30 g, 41.1 mmol, 1.37 equiv.), 2-chloromethyl-4-methoxy-3,5-dimethylpyridine hydrochloride (6.66 g, 30.0 mmol, 1.0 equiv.) was added. The resulting mixture was then extracted three times with toluene (20 mL) and the combined organic layers were dried with $MgSO_4$. This mixture was slowly added dropwise to a solution of LiCp (2.38 g, 33.0 mmol, 1.10 equiv.) in 10 mL THF at 0 °C. During the addition, an off-white precipitate formed and the reaction mixture turned brown. After allowing the mixture to stir overnight, an excess NH_4Cl (saturated in H_2O) was added and the layers were separated. The organic layer was then dried with $MgSO_4$ and the solvent was removed to obtain a dark, golden oil. Flash column chromatography was performed to purify the product (basic aluminum oxide, pentane/ethyl acetate = 1/4) to give clear light-yellow oil. The resulting product was a mixture of 2-(cyclopenta-1,3-dien-1-ylmethyl)-4-methoxy-3,5-dimethylpyridine and 2-(cyclopenta-1,4-dien-1-ylmethyl)-4-methoxy-3,5-dimethylpyridine (1:1 mixture). The product was highly sensitive to air, and the color of the pure product changed to pink within a few min of exposure to air at room temperature. To avoid the Diels-Alder dimerization reaction we immediately used the synthesized ligand for the next steps. The isolated yield was 46%. ^{1}H NMR (500 MHz, $CDCl_3$, 298 K): δ (ppm) 8.17 (d, 2H), 6.43–5.86 (m, 6H), 3.88, 3.71 (s, 6H), 2.91, 2.87 (2 × d, 4H), 2.20, 2.18 (2 × s, 12H). $^{13}C\{^1H\}$ NMR (125 MHz, $CDCl_3$, 298 K): δ (ppm) 171.09, 163.97, 163.92, 158.66, 158.33, 149.02, 146.09, 143.83, 134.41, 133.75, 132.24, 131.33, 127.82, 127.28, 124.58, 124.44, 124.09, 59.79, 43.38, 41.23, 37.87, 36.95, 14.17, 13.19, 11.20. HRMS (ESI+) for $C_{14}H_{18}NO^+$ $(M + H)^+$: calcd. 216.1388, found 216.1395.

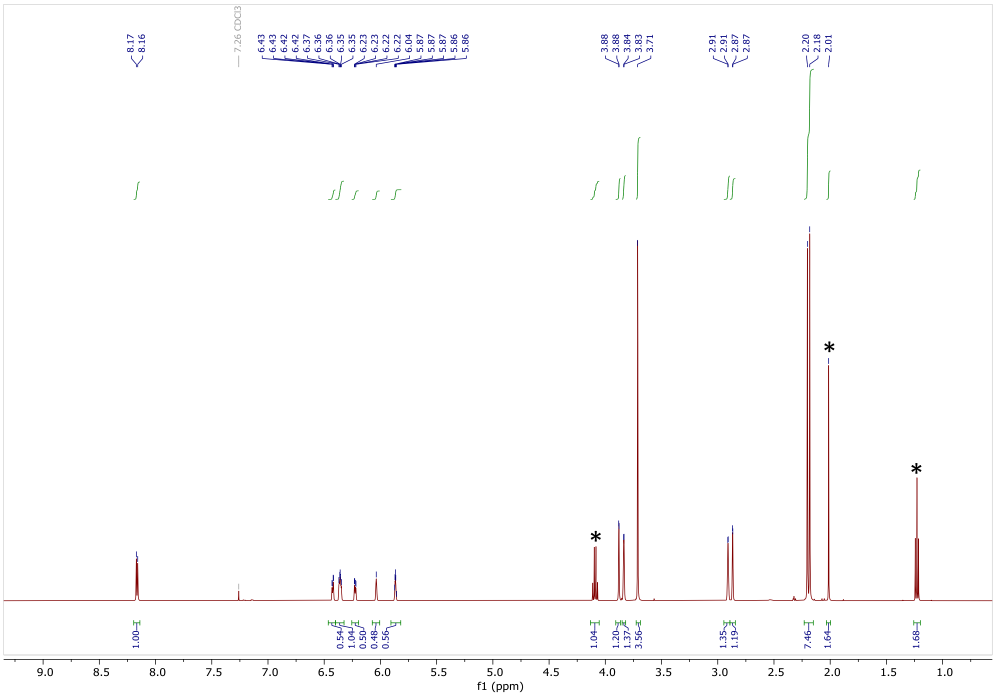

Figure S5. ^{1}H NMR spectrum (500 MHz, $CDCl_3$, 22 °C) of 2-(cyclopenta-1,3-dien-1-ylmethyl)-4-methoxy-3,5-dimethylpyridine and 2-(cyclopenta-1,4-dien-1-ylmethyl)-4-methoxy-3,5-dimethylpyridine isomers. (* Ethyl acetate).

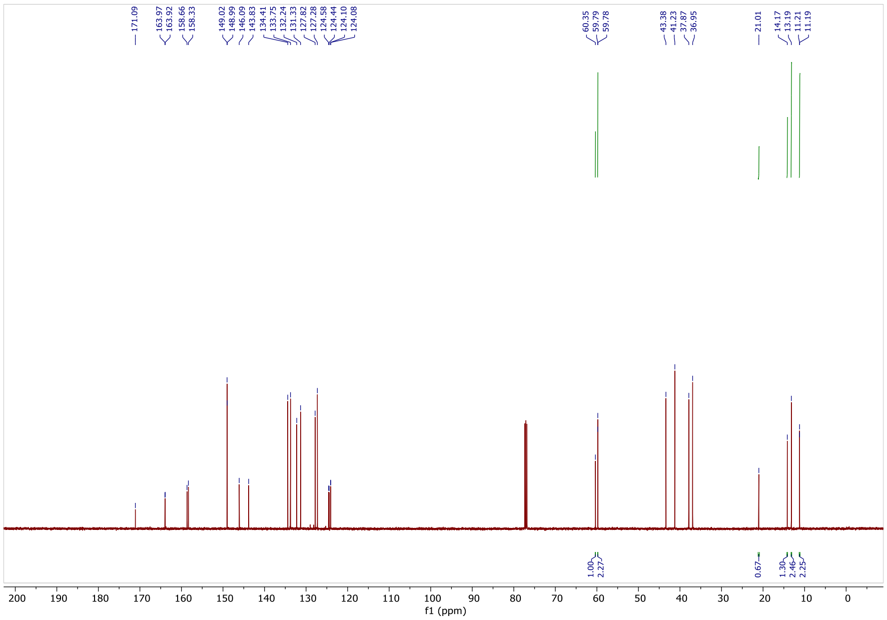

Figure S6. $^{13}C\{^{1}H\}$ NMR spectrum (125 MHz, $CDCl_3$, 22 °C) of 2-(cyclopenta-1,3-dien-1-ylmethyl)-4-methoxy-3,5-dimethylpyridine and 2-(cyclopenta-1,4-dien-1-ylmethyl)-4-methoxy-3,5-dimethylpyridine isomers.

3.2. Synthesis of Ferrocenes with Pendant Pyridine Arms

3.2.1. Synthesis of 1,1′-di(2-pyridylmethyl)ferrocene (1).

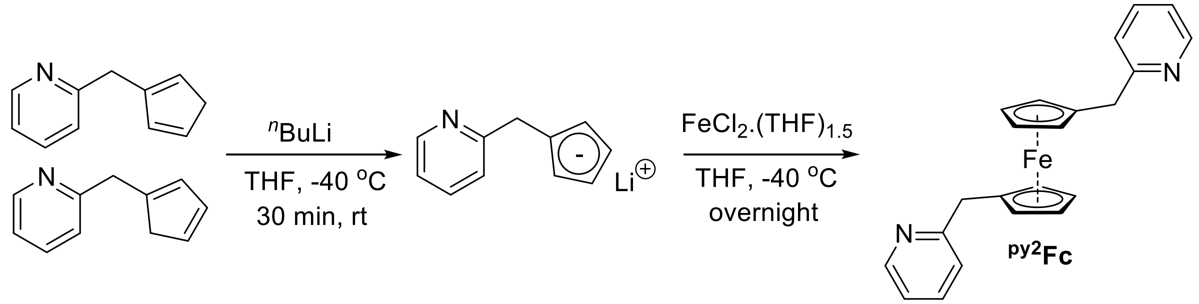

A mixture of 2-(cyclopenta-1,3-dien-1-ylmethyl)pyridine and 2-(cyclopenta-1,4-dien-1-ylmethyl)pyridine isomers (total 1.08 g, 6.87 mmol, 1.0 equiv.) was placed in a vial and dissolved in 15 mL THF in the glovebox. The solution was cooled to –40 °C in a freezer. Next, ^{n}BuLi (2.5 M in hexane, 1.1 equiv., 2.75 mL) was slowly added to the mixture at –40 °C and stirred at room temperature for 30 min. The color of the solution changed from yellow to brown. A slurry of $FeCl_2$•$(THF)_{1.5}$ (0.807 g, 0.5 equiv.) in 5 mL of THF was added to the reaction mixture at –40 °C. The reaction mixture was stirred overnight at room temperature, after which the brown-colored mixture was filtered through a syringe filter and all solvent was evaporated to dryness. The resulting product was purified by flash column chromatography using basic aluminum oxide and a DCM/MeOH mixture (20/1). This provided a yellow-colored solid with an isolated yield of 38%. ^{1}H NMR (500 MHz, $CDCl_3$, 298 K): δ (ppm) 8.50 (*o*-pyridine, s, 2H), 7.54 (*p*-pyridine, t, 2H), 7.08 (*m*-pyridine, d, 4H), 4.12, 4.05 (d, 8H), 3.85 (s, 4H). $^{13}C\{^1H\}$ NMR (125 MHz, $CDCl_3$, 298 K): δ (ppm) 161.26, 149.04, 136.37, 122.42, 121.13, 86.20, 69.61, 68.66, 38.69; Elemental analysis: calcd. for $C_{22}H_{20}FeN_2$ (**1**): C, 71.75; H, 5.47; N, 7.61. found: C, 71.84; H, 5.55; N, 7.57. HRMS (ESI+) for $C_{22}H_{21}FeN_2^+$ $(M + H)^+$: calcd. 369.1054, found 369.1060. The X-ray structure is shown in Figure S38.

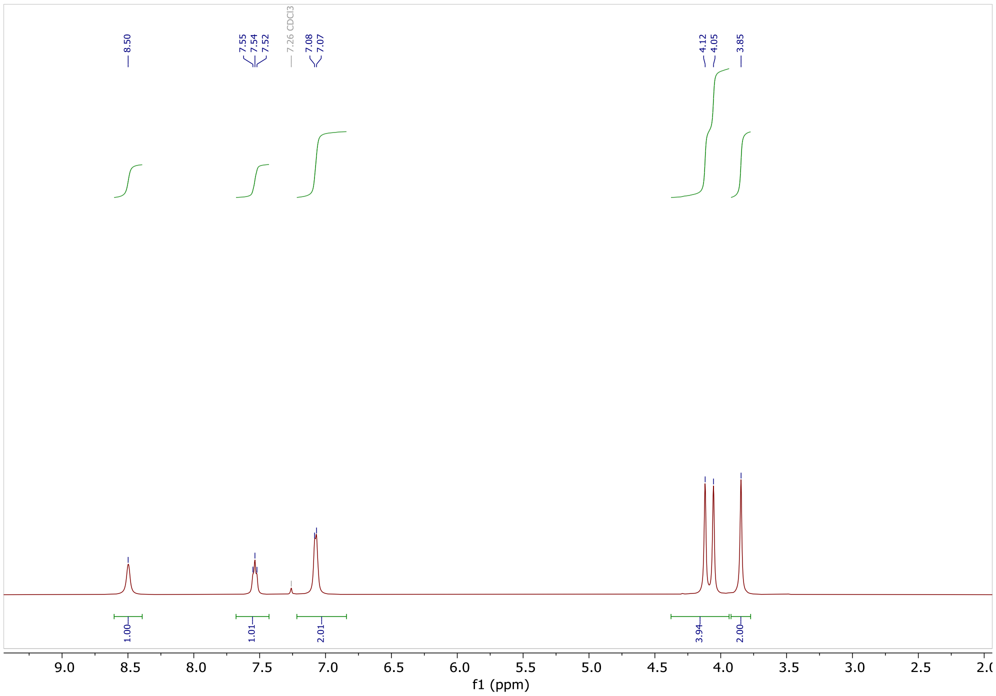

Figure S7. ^{1}H NMR spectrum (500 MHz, $CDCl_3$, 22 °C) of 1,1′-di(2-pyridylmethyl)ferrocene (**1**).

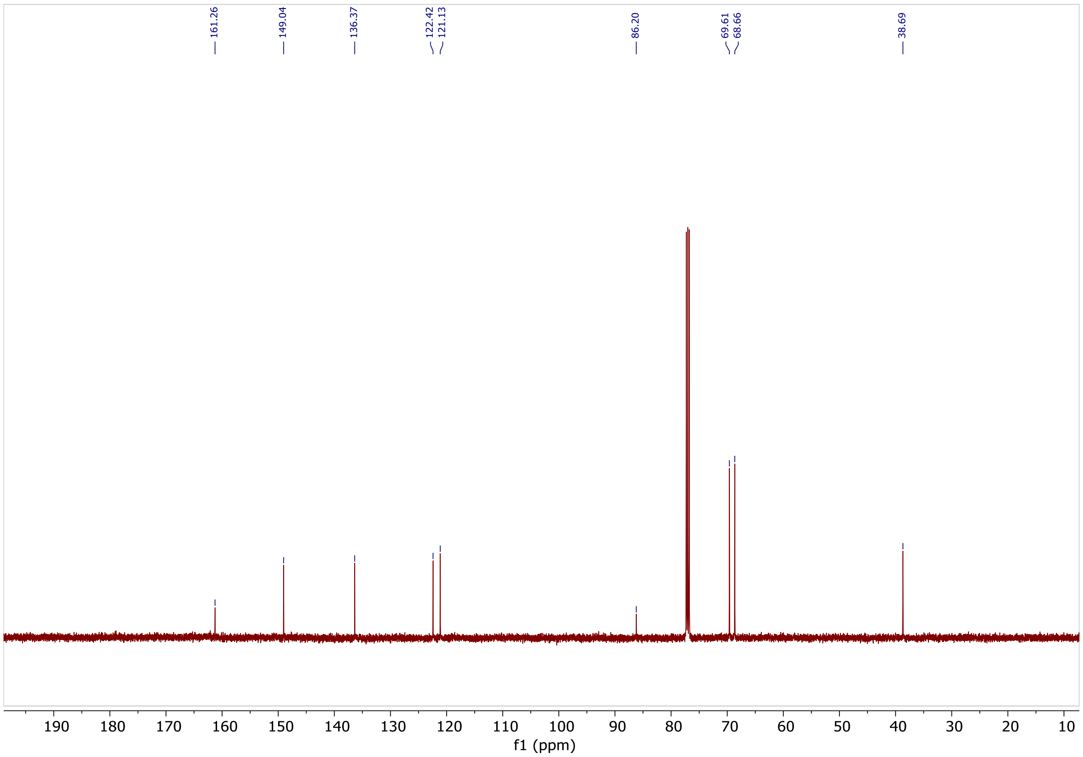

Figure S8. $^{13}C\{^{1}H\}$ NMR spectrum (125 MHz, $CDCl_3$, 22 °C) of 1,1′-di(2-pyridylmethyl)ferrocene (**1**).

3.2.2. Synthesis of 1,1′-di(6-methyl-2-pyridylmethyl)ferrocene (2).

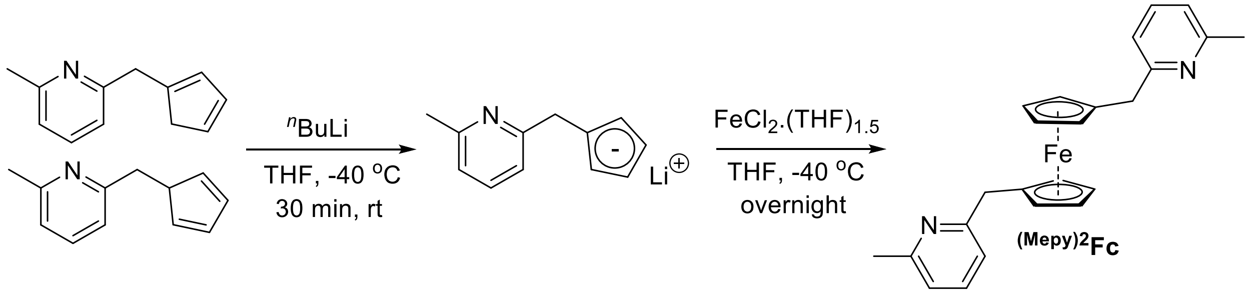

A mixture of 2-(cyclopenta-1,3-dien-1-ylmethyl)-2-methylpyridine and 2-(cyclopenta-1,4-dien-1-ylmethyl)-2-methylpyridine (total 1.08 g, 6.87 mmol, 1.0 equiv.) was placed in a 22 mL glass vial and dissolved in 15 mL THF in the glovebox. The solution was cooled to –40 °C in a freezer. Next, ^{n}BuLi (2.5 M in hexane, 1.1 equiv., 2.75 mL) was slowly added to the mixture at –40 °C and stirred at room temperature for 30 min. The color of the solution changed from yellow to brown. $FeCl_2$•$(THF)_{1.5}$ (0.807 g, 0.5 equiv.) was dissolved in 5 mL of THF and added to the reaction mixture at –40 °C. The reaction mixture was stirred overnight at room temperature, after which the brown-colored mixture was filtered through a syringe filter and all solvent was evaporated to dryness. The resulting product was purified by performing flash column chromatography using basic aluminum oxide and a DCM/MeOH mixture (20/1). This provided a yellow-colored solid with an isolated yield of 40%. ^{1}H NMR (500 MHz, $CDCl_3$, 298K): δ (ppm) 7.43 (t, 2H), 6.94 (d, 2H), 6.84 (d, 2H), 4.12, 4.06 (2 × d, 4H), 3.83 (s, 4H), 2.53 (s, 6H). ^{13}C{^{1}H} NMR (125 MHz, $CDCl_3$, 298K): δ (ppm) 160.68, 157.43, 136.56, 120.55, 119.15, 86.16, 69.71, 68.62, 38.71, 24.54; Elemental analysis: Calcd. for $C_{24}H_{24}FeN_2$ (**2**): C, 72.74; H, 6.10; N, 7.07. Found: C, 72.86; H, 6.16; N, 7.00. HRMS (ESI+) for $C_{24}H_{25}FeN_2^+$ $(M + H)^+$: calcd. 397.1367, found 397.1385. The X-ray structure is shown in Figure S39.

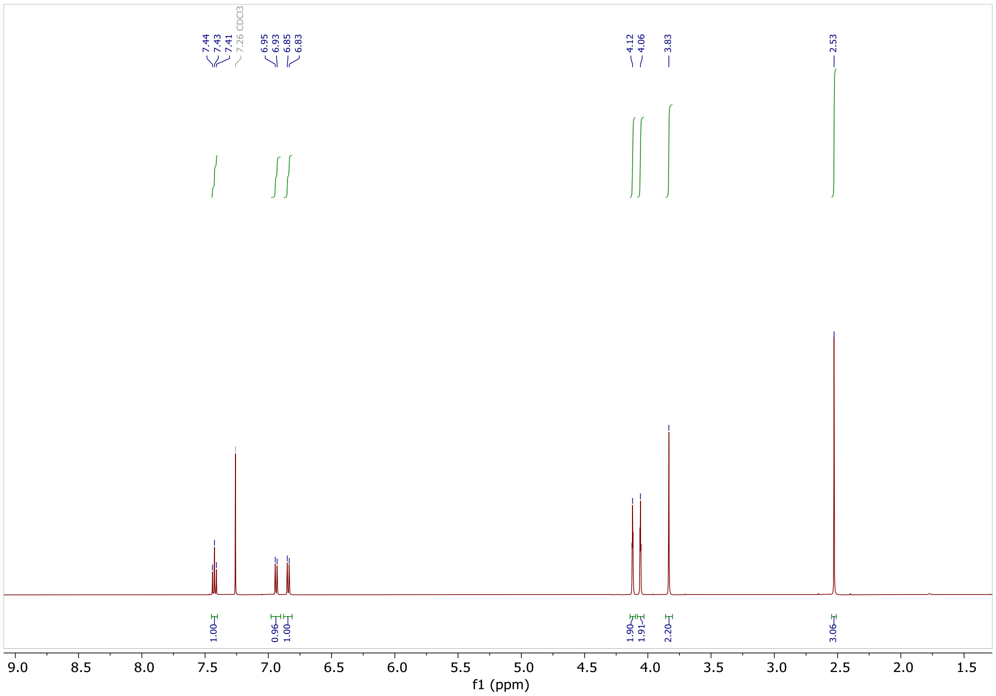

Figure S9. ^{1}H NMR spectrum (500 MHz, $CDCl_3$, 22 °C) of 1,1′-di(6-methyl-2-pyridylmethyl)ferrocene (**2**).

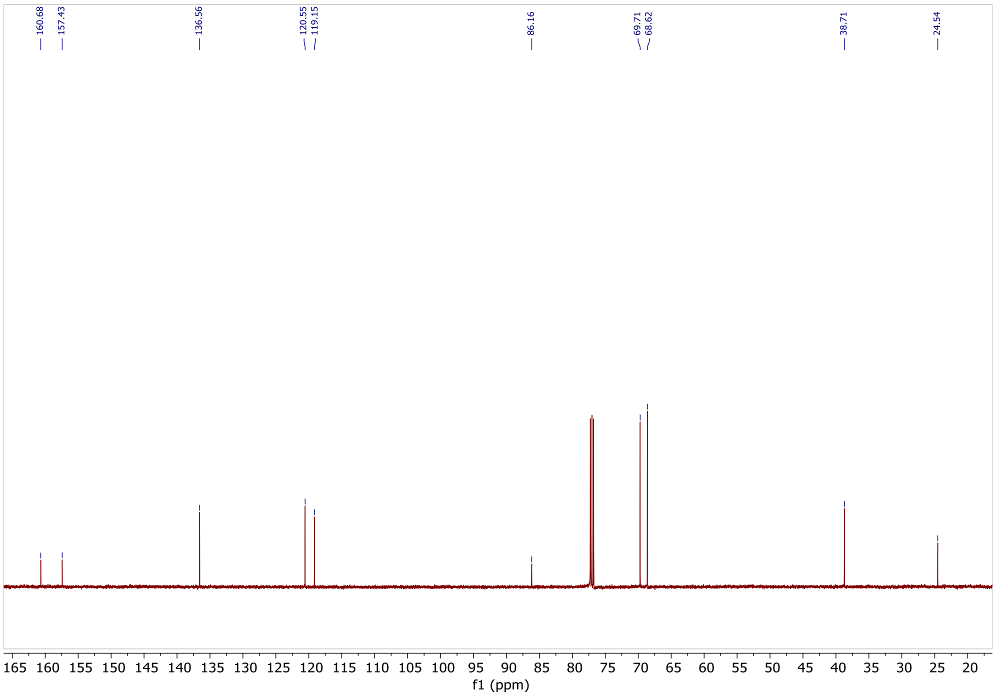

Figure S10. $^{13}C\{^{1}H\}$ NMR spectrum (125 MHz, $CDCl_3$, 22 °C) of 1,1′-di(6-methyl-2-pyridylmethyl)ferrocene (**2**).

3.2.3. Synthesis of 1,1′-di((4-methoxy-3,5-dimethyl)-2-pyridylmethyl)ferrocene (3).

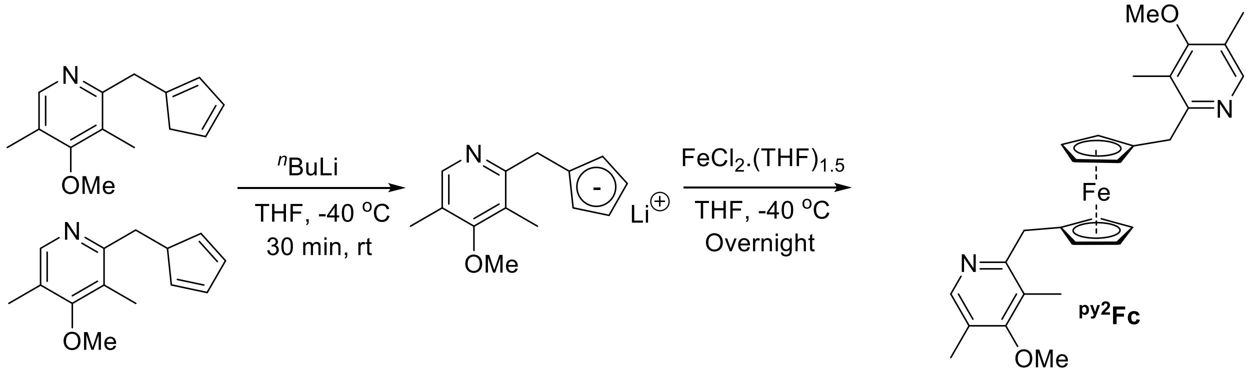

A mixture of 2-(cyclopenta-1,3-dien-1-ylmethyl)-4-methoxy-3,5-dimethylpyridine and 2-(cyclopenta-1,4-dien-1-ylmethyl)-4-methoxy-3,5-dimethylpyridine (total 1.00 g, 4.60 mmol, 1.0 equiv.) was placed in a 22 mL glass vial and dissolved in 15 mL THF in the glovebox. The solution was cooled to −40 °C in a freezer. Next, ^{n}BuLi (2.5 M in hexane, 1.0 equiv., 1.84 mL) was slowly added to the mixture at −40 °C and stirred at room temperature for 30 min. The color of the solution changed from yellow to brown. $FeCl_2$•$(THF)_{1.5}$ (0.540 g, 0.5 equiv.) was dissolved in 5 mL of THF and added to the reaction mixture at −40 °C. The reaction mixture was stirred overnight at room temperature, after which the brown-colored mixture was filtered through a syringe filter and all solvent was evaporated to dryness. The resulting product was purified by performing flash column chromatography using basic aluminum oxide and a DCM/MeOH mixture (20/1). This provided a yellow-colored solid with an isolated yield of 44%. ^{1}H NMR (500 MHz, $CDCl_3$, 298 K): δ (ppm) 8.15 (*o*-pyridine, s, 2H), 4.13 (s, 2H), 4.01 (t, 4H), 3.83 (s, 2H), 3.70 (s, 6H), 2.23, (s, 6H), 2.19 (s, 6H). $^{13}C\{^{1}H\}$ NMR (125 MHz, $CDCl_3$, 298 K): δ (ppm) 158.54, 155.19, 145.35, 142.89, 106.04, 86.55, 69.69, 68.35, 60.75, 55.58, 32.89; Elemental analysis: Calcd. for $C_{28}H_{32}FeN_2O_2$ (**3**): C, 69.42; H, 6.66; N, 5.78. Found: C, 69.47; H, 7.03; N, 5.91. HRMS (ESI+) for $C_{28}H_{33}FeN_2O_2^+$ $(M + H)^+$: calcd. 485.1892, found 485.1903. The X-ray structure is shown in Figure S40.

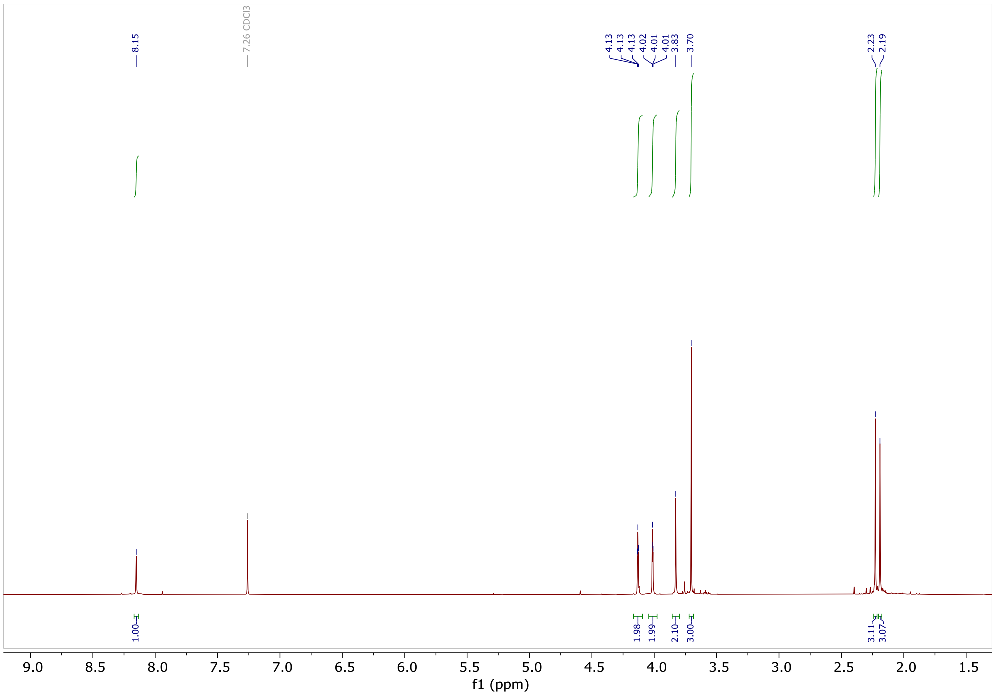

Figure S11. ^{1}H NMR spectrum (500 MHz, $CDCl_3$, 22 °C) of 1,1′-di((4-methoxy-3,5-dimethyl)-2-pyridylmethyl)ferrocene (**3**).

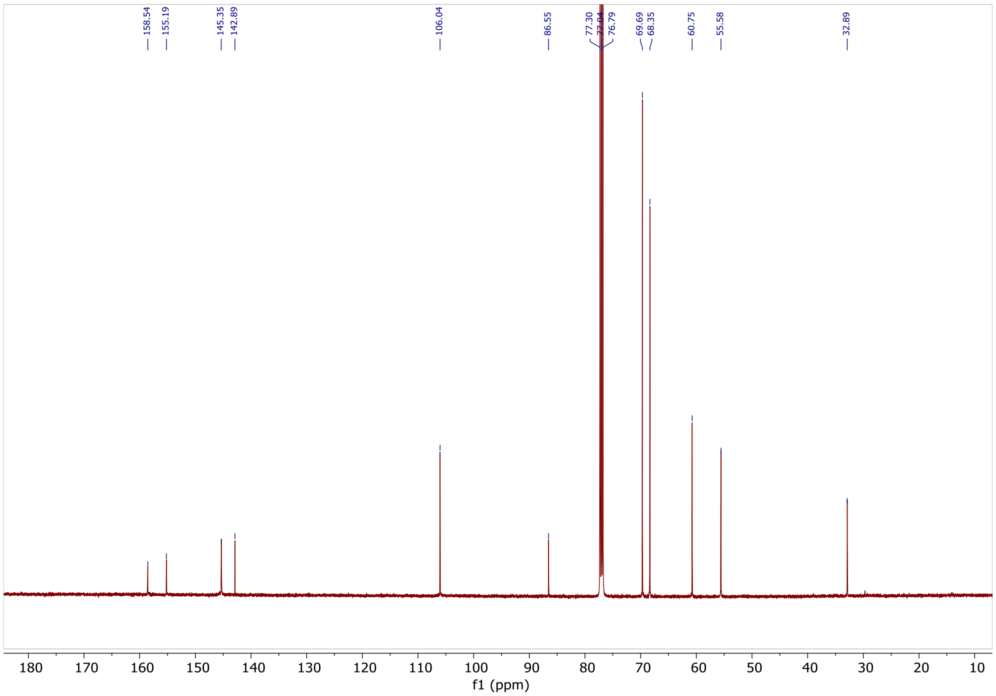

Figure S12. $^{13}C\{^{1}H\}$ NMR spectrum (125 MHz, $CDCl_3$, 22 °C) of 1,1′-di((4-methoxy-3,5-dimethyl)-2-pyridylmethyl)ferrocene (**3**).

3.2.4. Synthesis of 2-pyridylmethyl ferrocene, compound 4.

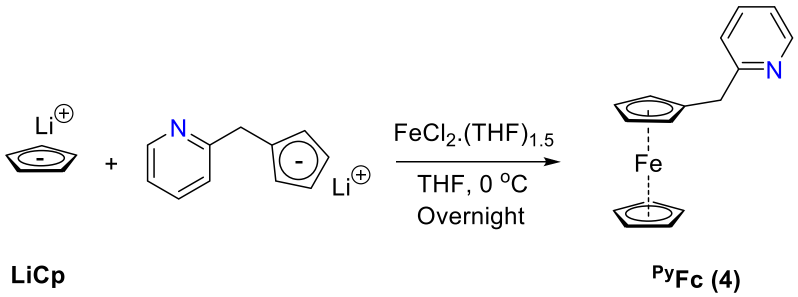

A mixture of 2-(cyclopenta-1,3-dien-1-ylmethyl)pyridine and 2-(cyclopenta-1,4-dien-1-ylmethyl)pyridine isomers (total 0.530 g, 3.37 mmol, 1.0 equiv.) was placed in a 22 mL glass vial and dissolved in 8 mL THF in the glovebox. The solution was cooled to −40 °C in a freezer. Next, nBuLi (2.5 M in hexane, 1.35 mL, 1.0 equiv.) was slowly added to the mixture at −40 °C and stirred at room temperature for 30 min. The color of the solution changed from yellow to brown.

In another vial, 0.243 g (3.37 mmol, 1.0 equiv.) of cyclopentadienyllithium was dissolved in 8 mL of THF in a glovebox. The resulting solution was then transferred to the previous reaction mixture vial and cooled to −40 °C. Next, a slurry of 0.792 g (1.0 equiv.) of $FeCl_2$•$(THF)_{1.5}$ in 5 mL of THF was added to the reaction mixture at −40 °C. The mixture was stirred overnight at room temperature. Afterwards, the brown-colored mixture was filtered through a syringe filter, and all solvent was evaporated to dryness.

The resulting product was purified by performing flash column chromatography using basic aluminum oxide. The yellow-colored compound was separated using a hexane/DCM mixture (1/4). This provided a yellow-colored solid with an isolated yield of 42%. ^{1}H NMR (500 MHz, $CDCl_3$, 298 K): δ (ppm) 8.51 (d, 1H), 7.56 (t, 1H), 7.09 (t, 2H), 4.18 - 4.10 (Cp-*H*, m, 9H), 3.88 (s, 2H). $^{13}C\{^1H\}$ NMR (125 MHz, $CDCl_3$, 298 K): δ (ppm) 161.25, 149.00, 136.39, 122.45, 121.15, 86.01, 68.86, 68.71, 67.75, 38.87. HRMS (ESI) for $C_{16}H_{16}FeN^+$ $(M + H)^+$: calcd. 278.0674, found 278.0698. The X-ray structure is shown in Figure S41.

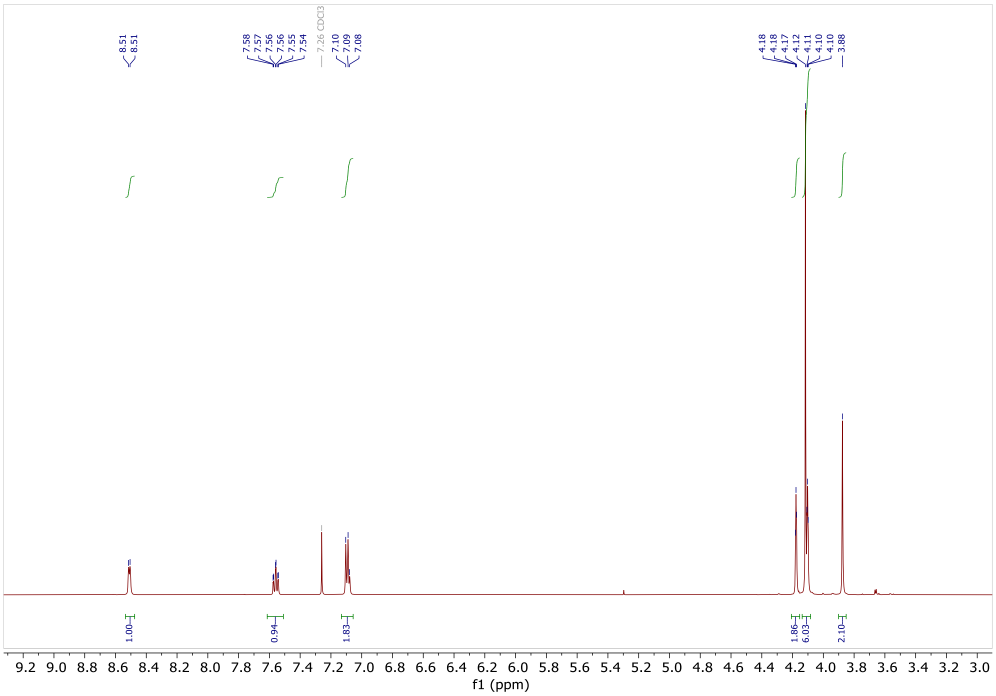

Figure S13. ^{1}H NMR spectrum (500 MHz, $CDCl_3$, 22 °C) of 2-pyridylmethyl ferrocene (**4**).

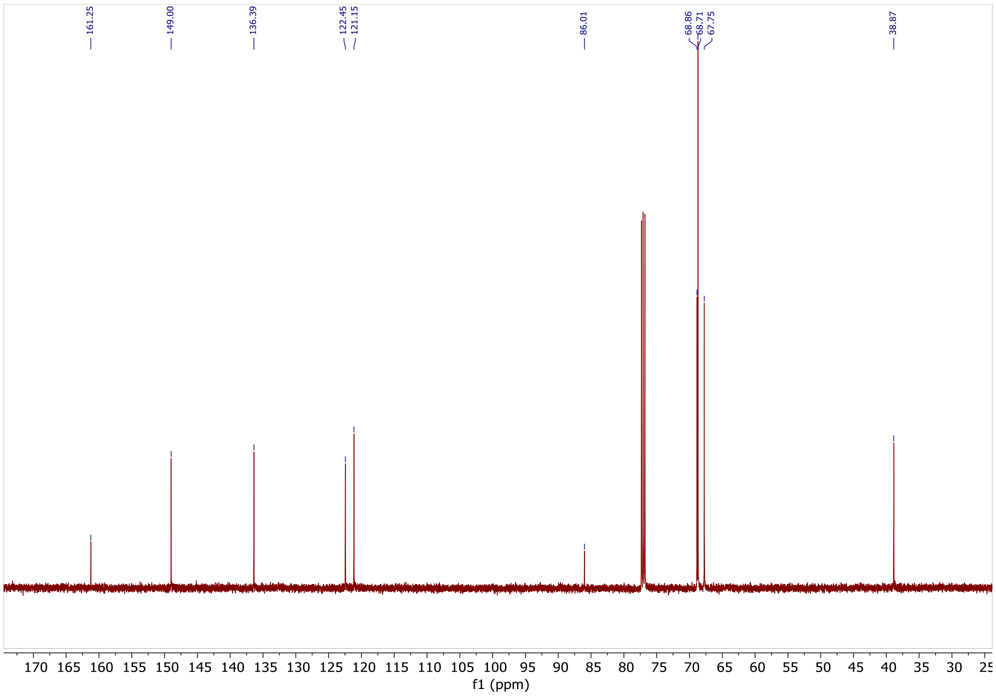

Figure S14. $^{13}C\{^{1}H\}$ NMR spectrum (125 MHz, $CDCl_3$, 22 °C) of 2-pyridylmethyl ferrocene (**4**).

4. Reaction of Gomberg's Dimer with •NH_2 Intermediate

4.1. Reaction of Gomberg's Dimer with Fc[BF_4] in Presence of Excess NH_3

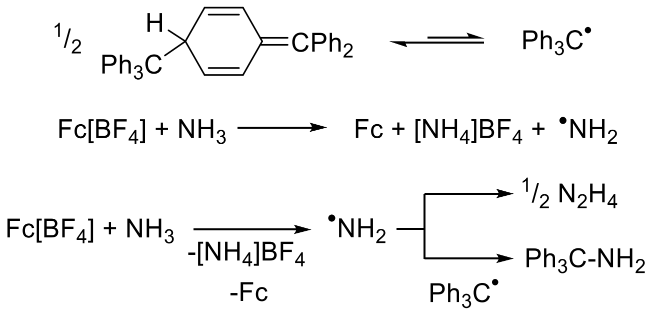

A solution of Gomberg's dimer solution with saturated NH_3 was prepared in the following way: A stock solution of Gomberg's dimer with an internal standard was prepared by dissolving $(Ph_3C)_2$•pentane (0.0082 g, 0.015 mmol) and 1,2,4,5-tetrachlorobenzene (0.0065 g, 0.030 mmol) in 2.00 mL acetone-d_6. The 1.2 mL stock solution was transferred to another vial and sealed with a rubber septum. The vial was taken outside the glove box and sparged the solution with anhydrous NH_3 for couple of min (3 min) to make the NH_3 saturated acetone solution. After that the vial was taken into the glove box. The volume of the solution was maintained to its initial volume by adding acetone-d_6 (as due to NH_3 sparging volume reduced by a few microliters).

300 µL of this stock solution containing Gomberg's dimer (0.0012 mmol), 1,2,4,5-tetrachlorobenzene as internal standard (0.0023 mmol) and NH_3 (0.450 mmol) was transferred to a vial to which 300 µL of the Fc[BF_4] stock solution (0.0045 mmol) was added at ambient temperature. The purple color solution turned colorless immediately; the vial was capped, and the contents stirred for 10 min. 500 µL of the reaction mixture was transferred to an NMR tube for ^{1}H NMR analysis. NMR yield of Fc (52%) was calculated based on 1,2,4,5-tetrachlorobenzene (s, δ 7.87 ppm). The formation of $Ph_3C–NH_2$ was confirmed by GC-MS. The GC-MS spectrum of the reaction mixture showed a molecular peak at 259 (*m/z*) that corresponds to the formation of trityl amine ($Ph_3C–NH_2$, *m/z* = 259.14) with matching isotopic distribution pattern. The yield of $Ph_3C–NH_2$ in this experiment was determined to be 32% based on quantitative GC/MS analysis derived from a calibration curve produced by using various ratios of $Ph_3C–NH_2$ and 1,2,4,5-tetrachlorobenzene. As part of the control experiment, we conducted a UV-vis study, during which it was observed that there is no reaction between Fc[BF_4] and Gomberg's dimer. Gomberg's dimer itself does not react with NH_3.

Note: One-half equivalent Gomberg's dimer was added with respect to Fc[BF_4].

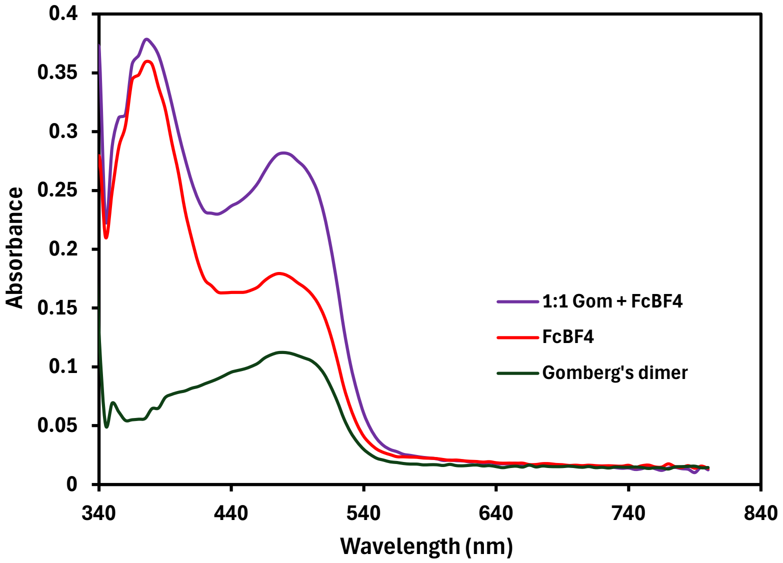

Figure S15: UV-vis study of (red) Fc[BF_4], (black) Gomberg's dimer and (purple) 1:1 mixture of Fc[BF_4] and Gomberg's dimer in acetone at room temperature. The purple spectrum is the additive of the other two spectra. The pathlength of the cuvette is 1 cm.

5. Determination of the Equilibrium Binding Constant Between Ammonia and 2-Me-pyridine (2py) and Pyridine-substituted Ferrocene

5.1. Determination of Equilibrium Constant Between Ammonia and 1,1′-di(2-pyridylmethyl)ferrocene (1)

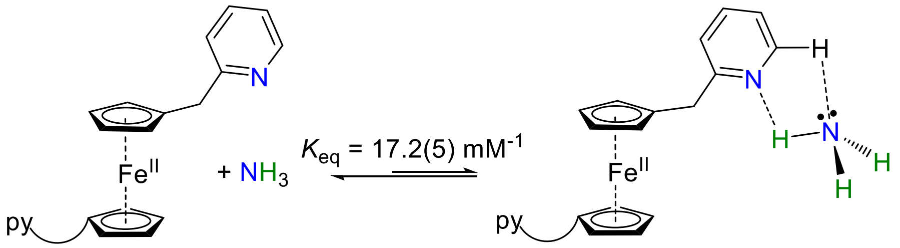

From the above chemical equilibrium, $K_{eq} = \frac{[Fc---NH_3]}{[Fc][NH_3]}$ in $CDCl_3$.

$$\text{or, } \frac{[Fc---NH_3]}{[Fc]} = K_{eq}[NH_3] \qquad \text{(eq. 1)}$$

A solution of **^{py2}Fc** with saturated NH_3 was prepared following the above procedure described for 2-Me-pyridine. A stock solution of **^{py2}Fc** (0.74 mg, 0.001 mmol) was prepared by dissolving it in 2.00 mL $CDCl_3$. The 1.0 mL stock solution was then transferred to a Schlenk flask and degassed using the freeze-pump-

thaw technique. Subsequently, the Schlenk was filled with anhydrous NH_3 and sparged the solution for 1 minute, resulting in an NH_3-saturated $CDCl_3$ solution. 0.5 mL of this solution was transferred to an NMR tube, and a ^{1}H NMR was recorded.

In another NMR tube, 0.5 mL of the stock solution of **^{py2}Fc** was taken, and a ^{1}H NMR was recorded. Ammonia-saturated **^{py2}Fc** solution from the previous NMR tube was then added to this solution, and NMR spectra were recorded at intervals until 129 equivalents of NH_3 were reached.

The amount of ammonia was calculated from the integration of ammonia and *ortho*-proton signals of two pyridines in **^{py2}Fc**. The NMR spectrum showed a shift of the *ortho*-proton towards the upfield region (Figure S16) with increasing ammonia concentration. Assuming 100% **^{py2}Fc** interacting with ammonia at saturated ammonia concentration, the equilibrium constant was determined using the equation 1 to be 17.2(5) mM^{-1}.

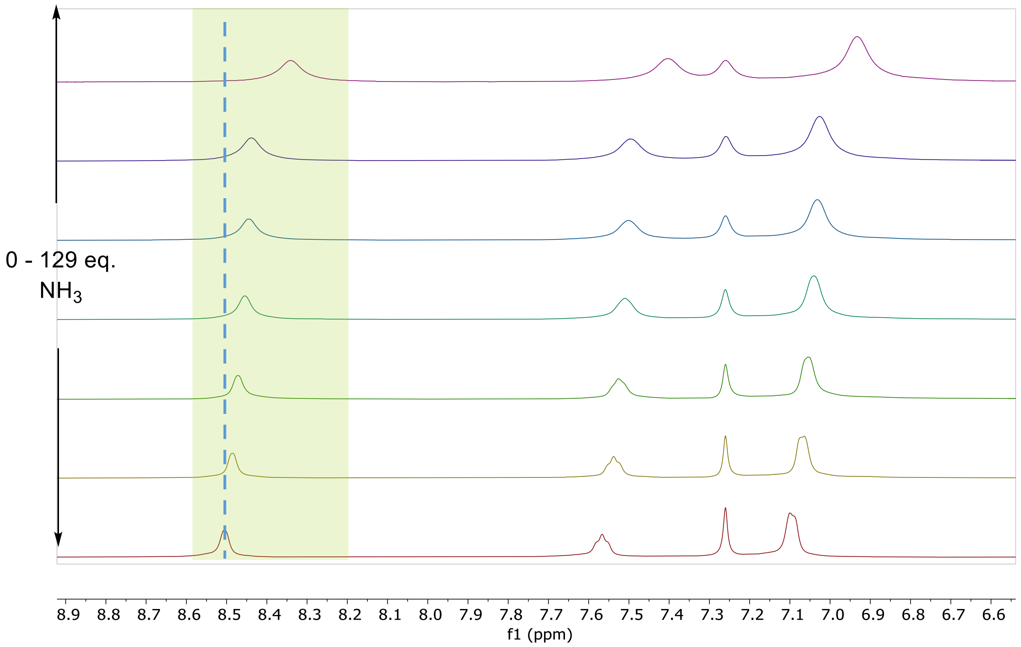

Figure S16. Stacked ^{1}H NMR spectra (500 MHz, $CDCl_3$, 298 K) of 2-Me-pyridine in presence of various ammonia concentrations.

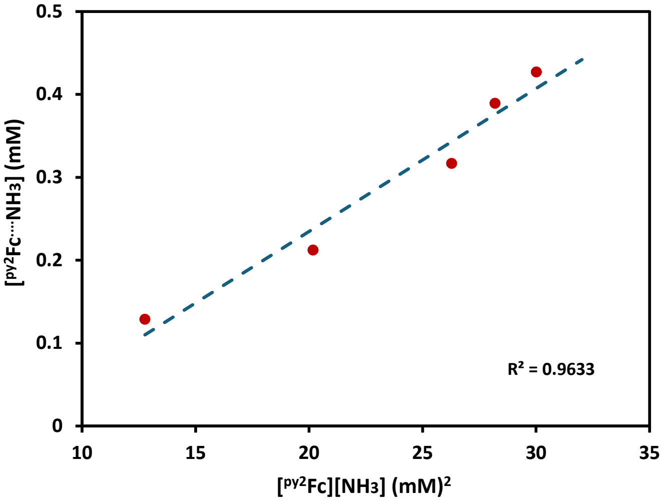

Figure S17. Determination of equilibrium constant between **^{py2}Fc** (**1**) and NH_3 in $CDCl_3$. The data points are extracted from the ^{1}H NMR shown in Figure S16.

5.2. Determination of Equilibrium Constant Between Ammonia and 2-Me-pyridine (2py)

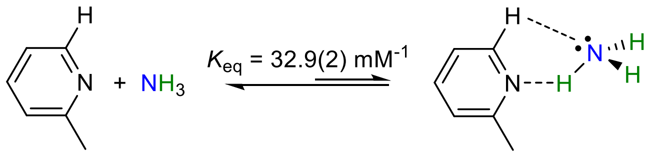

From the above chemical equilibrium, $K_{eq} = \frac{[2-Me-py---NH_3]}{[2-Me-py][NH_3]}$ in $CDCl_3$.

$$\text{or, } \frac{[2-Me-py---NH_3]}{[2-Me-py]} = K_{eq}[NH_3] \qquad \text{(eq. 2)}$$

A solution of 2-Me-pyridine (**2py**)with saturated NH_3 was prepared as follows: A stock solution of **2py** (0.93 mg, 0.001 mmol) was prepared by dissolving it in 5.00 mL $CDCl_3$. The 1.0 mL stock solution was then transferred to a Schlenk flask and degassed using the freeze-pump-thaw technique. Subsequently, the Schlenk was filled with anhydrous NH_3 and sparged the solution for 1 minute, resulting in an NH_3-saturated $CDCl_3$ solution. 0.5 mL of this solution was transferred to an NMR tube, and a ^{1}H NMR was recorded with a 25-sec delay time.

In another NMR tube, 0.5 mL of the stock solution of **2py** was taken, and a ^{1}H NMR was recorded with a 25-second delay time. Ammonia-saturated **2py** solution from the previous NMR tube was then added to this solution, and NMR spectra were recorded at intervals until 114 equivalents of NH_3 were reached.

The amount of ammonia was calculated from the integration of ammonia and *ortho*-proton signals of **2py**. The NMR spectrum showed a shift of the *ortho*-proton towards the upfield region (Figure S18) with

increasing ammonia concentration. Assuming 100% 2-Me-pyridine interacting with ammonia at saturated ammonia concentration, the equilibrium constant was determined using the equation 1 to be 32.9(2) mM^{-1}.

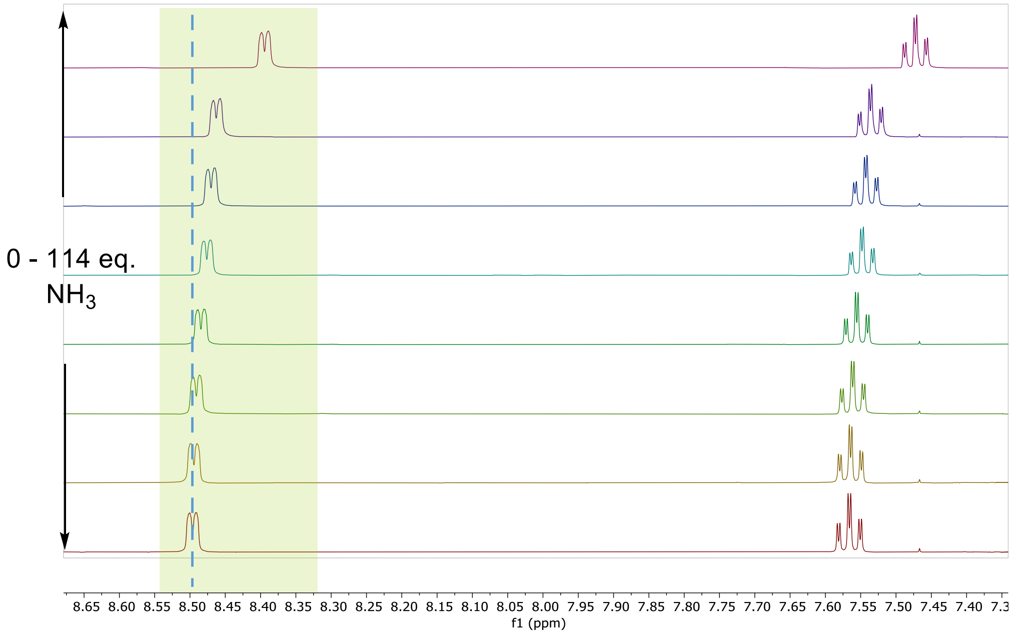

Figure S18. Stacked ^{1}H NMR spectra (500 MHz, $CDCl_3$, 298 K) of 2-Me-pyridine (**2py**) in presence of various concentrations of NH_3.

6. Electrochemistry

Cyclic voltammetry and chronoamperometry experiments were carried out at room temperature outside the glovebox at atmosphere pressure using a BASi Epsilon potentiostat with three electrodes setup. Glassy carbon (disk: A = 0.07 cm^2, plate: A = 1.2 cm^2), a custom silver/silver nitrate/acetonitrile and a platinum wire/mesh were used as the working, reference and auxiliary electrodes, respectively. Dry acetonitrile and DMSO stored over activated molecular sieves were used containing 100 mM $[Bu_4N]PF_6$ as the supporting electrolyte. The potential of the reference electrode was measured vs ferrocene/ferrocenium couple in MeCN and DMSO as required.

A saturated solution of NH_3 in DMSO containing 100 mM $[Bu_4N]PF_6$ was prepared by sparging anhydrous NH_3 gas into 20 mL DMSO for 30 min. The concentration of NH_3 in NH_3 saturated DMSO was measured by ^{1}H NMR in DMSO-*d₆* containing 100 mM $[Bu_4N]PF_6$. Anhydrous NH_3 was sparged for 3 min into 2 mL DMSO-d_6 containing 100 mM $[Bu_4N]PF_6$ and then ^{1}H NMR was taken with 500 µL solution. Comparing the integration values of NH_3 and $[Bu_4N]PF_6$ we have determined the concentration of NH_3. This experiment was done 3 times and the average concentration of NH_3 was measured to be 2.40(1) M in

DMSO. The concentration of NH_3 in NH_3 saturated MeCN with 100 mM $[Bu_4N]PF_6$ was measured in previous work by our research lab to be 1.30 M.[4]

DMSO/electrolyte (100 mM $[Bu_4N]PF_6$) solutions of lower NH_3 concentration than 2.4 M were prepared by dilution by adding measured amounts of DMSO (containing 100 mM $[Bu_4N]PF_6$) to the NH_3 saturated solution. NH_3 saturated 1.0 mM catalyst solution was prepared by sparging anhydrous NH_3 gas into 20 mL DMSO containing 1 mM catalyst for 30 min. NH_3 / $[Bu_4N]PF_6$ solutions with lower NH_3 concentrations were prepared by adding measured amounts of DMSO containing 1.0 mM catalyst and 100 mM $[Bu_4N]PF_6$ to the NH_3 saturated catalyst solution.

The effect of added 2-methylpyridine to the electrocatalytic response of ferrocene (2.4 mM) in NH_3 saturated DMSO was examined by increasing the [2-Mepy] concentration from 10.6 mM to 20.0 mM. This data is illustrated in Figure 4b.

7. Determination of Overpotential of Ammonia Oxidation in MeCN Solvent

While most of our electrochemical experiments have been conducted in DMSO, MeCN is widely recognized as a suitable solvent for homogeneous electrochemistry. Therefore, we have chosen to assess the overpotential in MeCN, where the thermodynamic potential for ammonia oxidation has been scaled to 0.939 V.

$$8\ NH_3 \longrightarrow N_2 + 6\ NH_4^{\oplus} + 6\ e^- \qquad E = -0.94\ V\ vs\ Fc^{+/0}$$

Cyclic Voltammograms of 1, 2, and 3 in MeCN in presence and absence of 1.3 M NH_3.

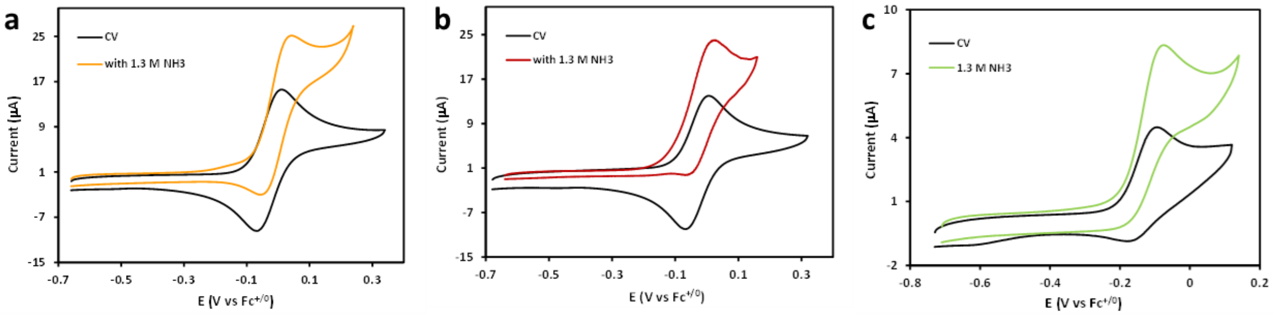

Figure S19. Overlay of cyclic voltammograms of (a) **1**, (b) **2**, and (c) **3** in MeCN in presence and absence of 1.3 M NH_3 Stacked.

The onset potential of ammonia oxidation of the $^{\mathbf{py2}}$**Fc** (**1**) catalyst is −120 mV vs. $Fc^{+/0}$ (Figure S19a) in MeCN. Hence, the onset overpotential is 820 mV.

The onset potentials of catalysts **2** and **3** are -140 and -170 mV vs. $Fc^{+/0}$ (Figures S19b and S19c) in MeCN corresponding to onset overpotentials of 800 and 770 mV, respectively.

8. Determination of Diffusion Coefficient of Substituted Ferrocene Complexes by Cyclic Voltammetry in DMSO

8.1. Determination of Diffusion Coefficient of ^{py2}Fc (1) in DMSO

The diffusion coefficient of ^{py2}**Fc** was determined using the Randles-Sevcik equation:

$$I_p = 0.4463\sqrt{\frac{F}{RT}}.\sqrt[3]{n_p}.FA[C_0]\sqrt{D\,v} \qquad \text{(eq. 3)}$$

Where, I_p is the peak current, F is Faraday's constant (F = 96485 C mol^{-1}), R is the universal gas constant (R = 8.314 J K^{-1} mol^{-1}), T is temperature (T = 300 K), n_p is the number of electrons transferred, A is the active surface area of the electrode, D is the diffusion coefficient of the complex, [C_0] is the concentration of the catalyst, and v is the scan rate (V/s). Using this equation, the electrochemical diffusion coefficient of **1** was determined to be 1.54 × 10^{-6} cm^{2}s^{-1}.

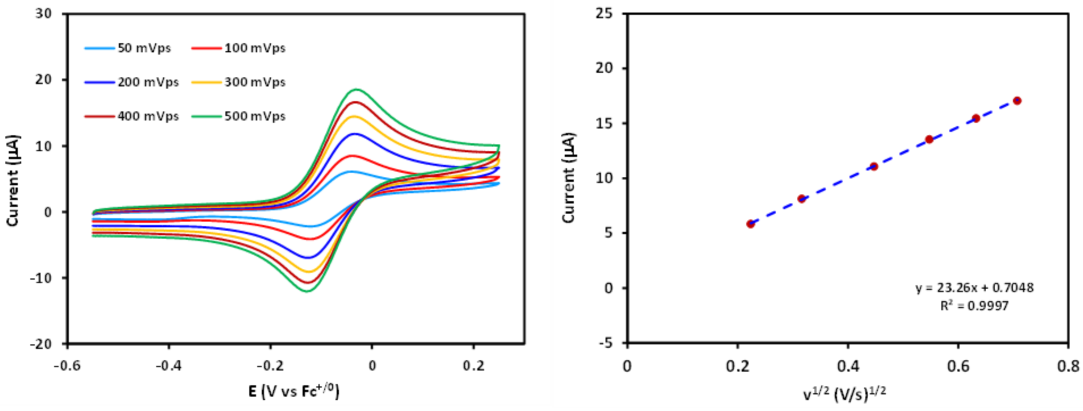

Figure S20. Cyclic voltammograms of 1.0 mM ^{py2}**Fc** (**1**) in DMSO at various scan rates (left). Peak current (I_p) vs square root of scan rates of the $Fe^{III/II}$ couple showing linearity (right). Conditions: 100 mM $[Bu_4N]PF_6$ supporting electrolyte; GC working, Pt counter and $Ag/AgNO_3$ reference electrodes are used.

8.2. Determination of Diffusion Coefficient of $^{(Mepy)2}$Fc (2) in DMSO

Using equation 3, the electrochemical diffusion coefficient of **2** was determined to be 2.11 × 10^{-6} cm^{2}s^{-1}.

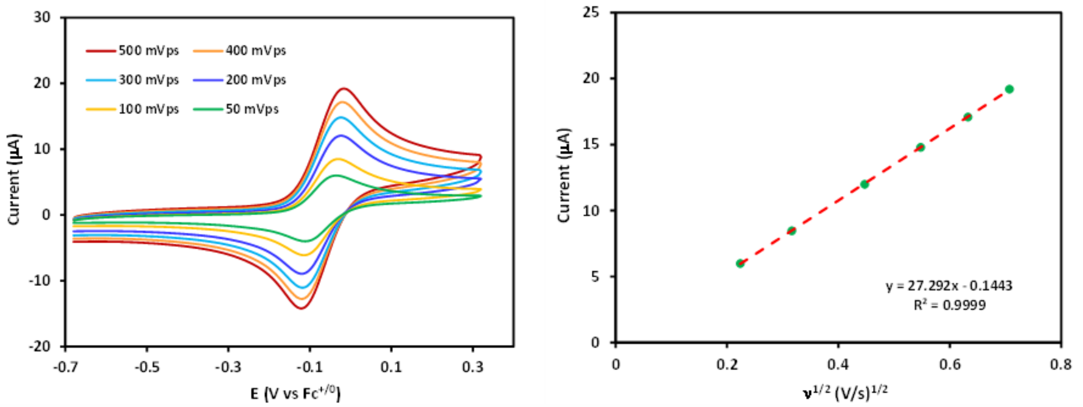

Figure S21. (left) Cyclic voltammograms of 1.0 mM **$^{(Mepy)2}$Fc** (**2**) in DMSO at various scan rates. (right) Peak current (I_p) vs square root of scan rates of the $Fe^{III/II}$ couple showing linearity. Conditions: 100 mM $[Bu_4N]PF_6$ supporting electrolyte; GC working, Pt counter and $Ag/AgNO_3$ reference electrodes are used.

8.3. Determination of Diffusion Coefficient of $^{(Me2OMe)2}$Fc (3) in DMSO

Using equation 3, the electrochemical diffusion coefficient of **3** was determined to be 0.77×10^{-6} cm^2s^{-1}.

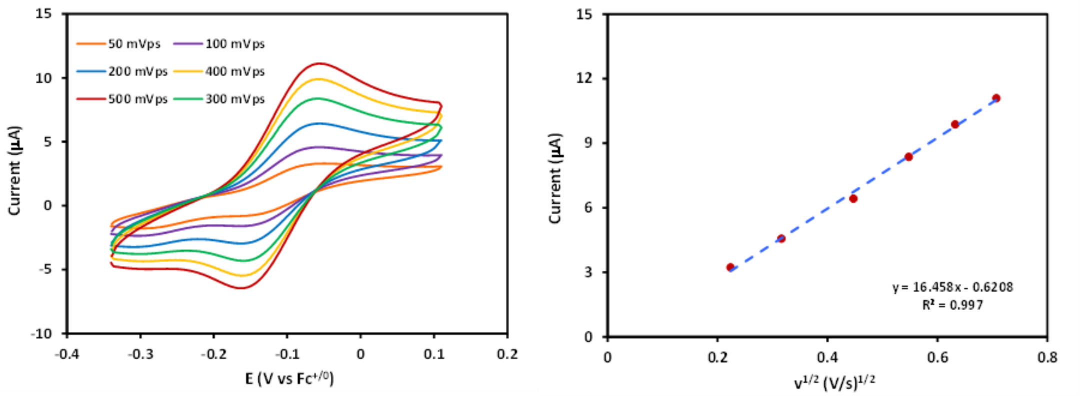

Figure S22. (left) Cyclic voltammograms of 1.0 mM **$^{(Me2OMepy)2}$Fc** (**3**) in DMSO at different scan rates. (right) Peak current (I_p) vs square root of scan rates of the $Fe^{III/II}$ couple showing linearity. Conditions: 100 mM $[Bu_4N]PF_6$ supporting electrolyte; GC working, Pt counter and $Ag/AgNO_3$ reference electrodes are used.

8.4. Determination of Diffusion Coefficient of ^{py}Fc (4) in DMSO

Using equation 3, the electrochemical diffusion coefficient of **4** was determined to be 2.44×10^{-6} cm^2s^{-1}.

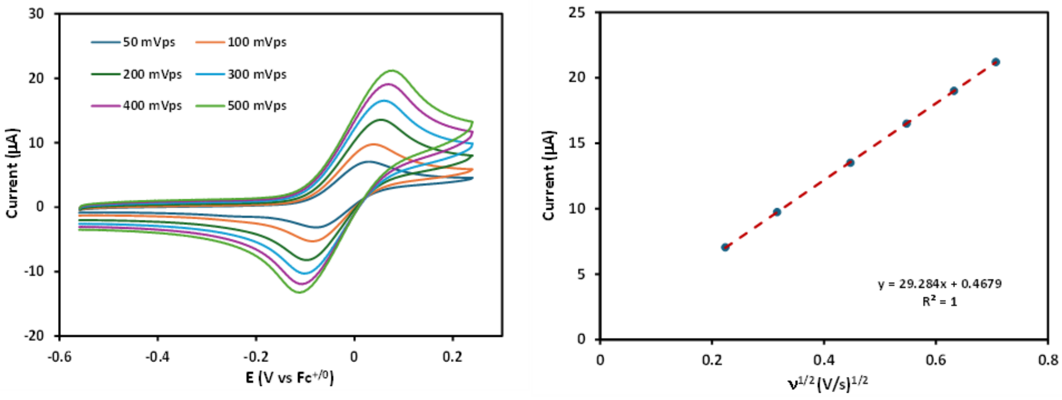

Figure S23. (left) Cyclic voltammograms of 1.0 mM **^{py}Fc** (**4**) in DMSO at different scan rates. (right) Peak current (I_p) vs square root of scan rates of the $Fe^{III/II}$ couple showing linearity. Conditions: 100 mM $[Bu_4N]PF_6$ supporting electrolyte; GC working, Pt counter and $Ag/AgNO_3$ reference electrodes are used.

9. Rate and Mechanistic Information of Ferrocene Complexes from Cyclic Voltammetry Experiments

9.1. Order in [catalyst]

From the CV data shown in Figures 6b and S24a, we have observed that the catalytic peak current (I_{cat}) for **^{py2}Fc** varies linearly with the [catalyst]. Following the equation $I_{cat} = n_{cat}FA[cat](Dk_{obs}[NH_3])^{1/2}$, the electrocatalytic process is first order in [catalyst].

Likewise, based on the data presented in Figures S25a and S26a, the electrocatalytic process exhibits a first-order dependency on [catalyst] for complexes **2** and **3**.

9.2. Order in [NH_3]

From the CV data shown in Figures 6c and S24b, we have observed that the catalytic peak current (I_{cat}) varies linearly with the $[NH_3]^{1/2}$. Following the equation $I_{cat} = n_{cat}FA[cat](Dk_{obs}[NH_3])^{1/2}$ the electrocatalytic process is first order in [NH_3]. This is true for all the three complexes (Figures S25b & 26b).

9.3. TOF_{max} Calculation from Cyclic Voltammetry Experiments

The TOF value for **1** was derived using the equation[5] below from the catalytic cyclic voltammograms recorded in NH_3 saturated DMSO solutions in presence of 1.0 mM catalyst.

$$\frac{I_{cat}}{I_p} = \frac{n_{cat}}{0.4463}.\sqrt{\frac{RT}{F}}.\sqrt{TOF_{max}}\,.\,\nu^{-\frac{1}{2}}$$

$$\frac{I_{cat}}{I_p} = 13.44.\sqrt{\frac{RT}{F}}.\sqrt{TOF_{max}}\,.\,\nu^{-\frac{1}{2}}$$

Where, I_p is peak current obtained from $Fe^{III/II}$ redox process, I_{cat} is the catalytic current, n_{cat} is the number of electrons required for the catalytic reaction (n_{cat} = 6 for the oxidation of NH_3 to N_2), F is Faraday's constant (F = 96500 C mol^{-1}), R is the universal gas constant (R = 8.314 $J.K^{-1}.mol^{-1}$), T is temperature (T = 298 K) and v is the scan rate. The TOF_{max} was calculated from the slope of I_{cat}/I_p vs $v^{-1/2}$ plot.

From I_{cat}/I_p vs $v^{-1/2}$ plot we determined the slope = 0.396. Therefore, TOF_{max} = 0.034 ± 0.002 s^{-1} = 125 h^{-1}.

Likewise, we have determined the maximum turnover frequency (TOF_{max}) for complexes **2** (Figure S25c) and **3** (Figure S26c) to be 223 and 560 h^{-1}, respectively.

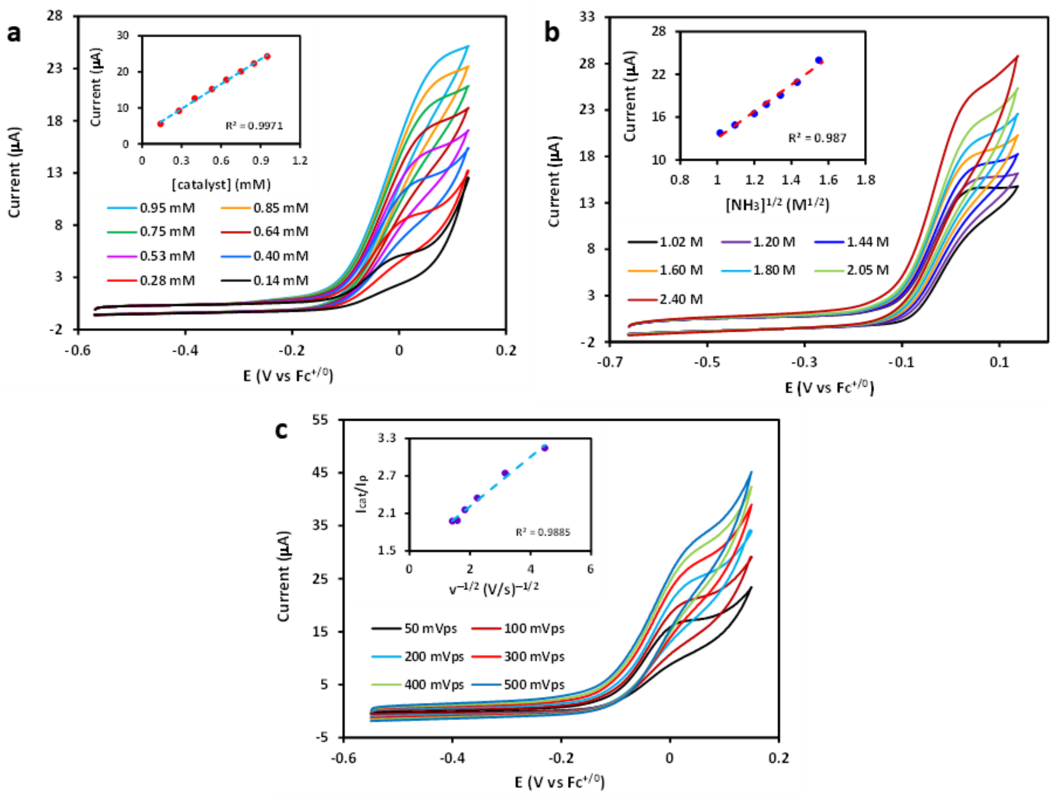

Figure S24. (a) Cyclic voltammograms of 0.14 – 0.95 mM **^{py2}Fc** (**1**) in 2.4 M NH_3 in DMSO; scan rate: 100 mVs^{-1}. (**b**) Cyclic voltammograms of 1.0 mM **^{py2}Fc** (**1**) in DMSO containing 1.02 – 2.40 M NH_3; scan rate: 100 mVs^{-1}. (**c**) Cyclic voltammograms of 1.0 mM **^{py2}Fc** (**1**) in DMSO containing 2.4 M NH_3 at scan rates of 50 – 500 mVs^{-1}. (inset) I_{cat}/I_p vs $v^{-1/2}$ plot. Conditions: 100 mM $[Bu_4N]PF_6$ supporting electrolyte with GC working, Pt counter and $Ag/AgNO_3$ reference electrodes.

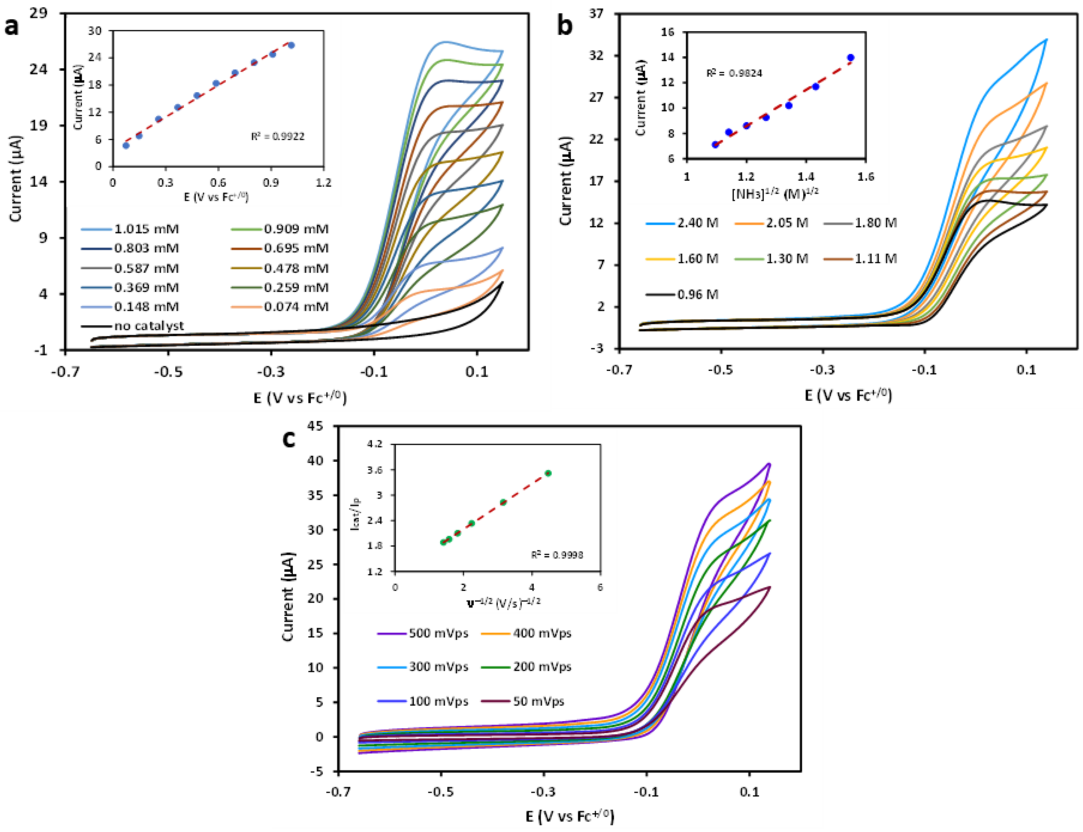

Figure S25. (a) Cyclic voltammograms of 0.07 – 1.01 mM **$^{(Mepy)2}$Fc** (**2**) in 2.4 M NH_3 in DMSO; scan rate: 100 mVs^{-1}. (**b**) Cyclic voltammograms of 1.0 mM **$^{(Mepy)2}$Fc** (**2**) in DMSO containing 0.96 – 2.40 M NH_3; scan rate: 100 mVs^{-1}. (**c**) Cyclic voltammograms of 1.0 mM **$^{(Mepy)2}$Fc** (**2**) in DMSO containing 2.4 M NH_3 at scan rates of 50 – 500 mVs^{-1}. (inset) I_{cat}/I_p vs $\nu^{-1/2}$ plot. Conditions: 100 mM $[Bu_4N]PF_6$ supporting electrolyte with GC working, Pt counter and Ag/$AgNO_3$ reference electrodes.

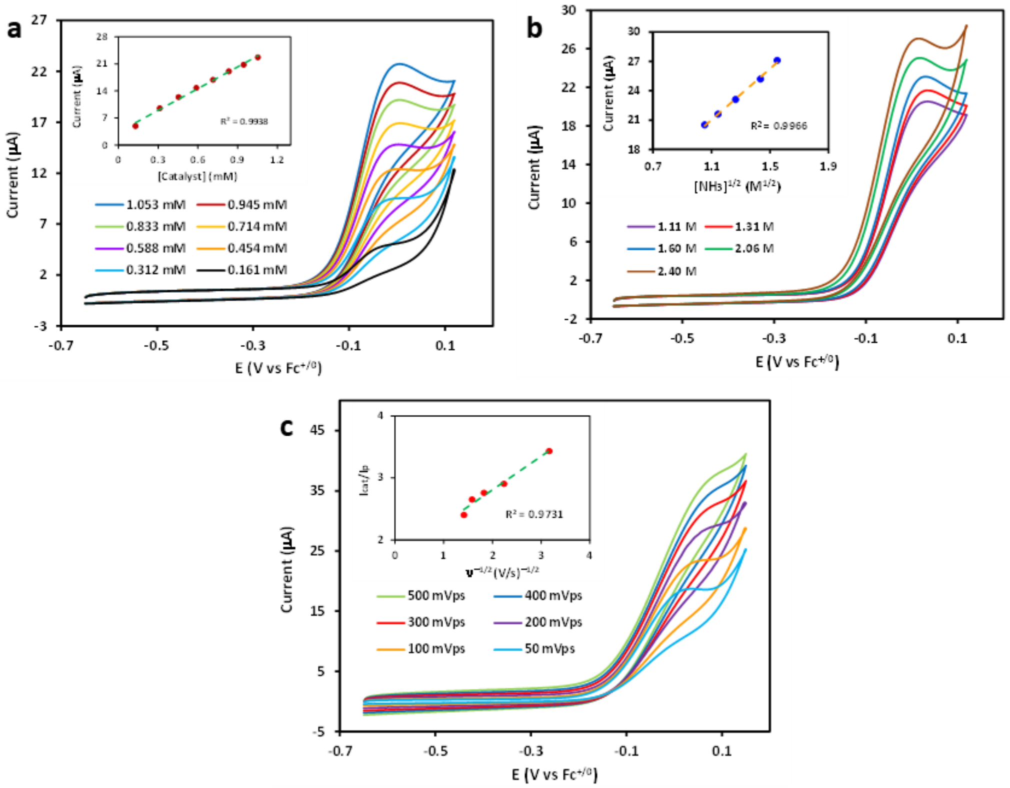

Figure S26. (**a**) Cyclic voltammograms of 0.16 – 1.05 mM $^{(Me2OMepy)2}$**Fc** (**3**) in 2.4 M NH_3 in DMSO; scan rate: 100 mVs^{-1}. (**b**) Cyclic voltammograms of 1.0 mM $^{(Me2OMepy)2}$**Fc** (**3**) in DMSO containing 1.11 – 2.40 M NH_3; scan rate: 100 mVs^{-1}. (**c**) Cyclic voltammograms of 1.0 mM $^{(Me2OMepy)2}$**Fc** (**3**) in DMSO containing 2.4 M NH_3 at scan rates of 50 – 500 mVs^{-1}. (inset) I_{cat}/I_p vs $v^{-1/2}$ plot. Conditions: 100 mM $[Bu_4N]PF_6$ supporting electrolyte with GC working, Pt counter and $Ag/AgNO_3$ reference electrodes.

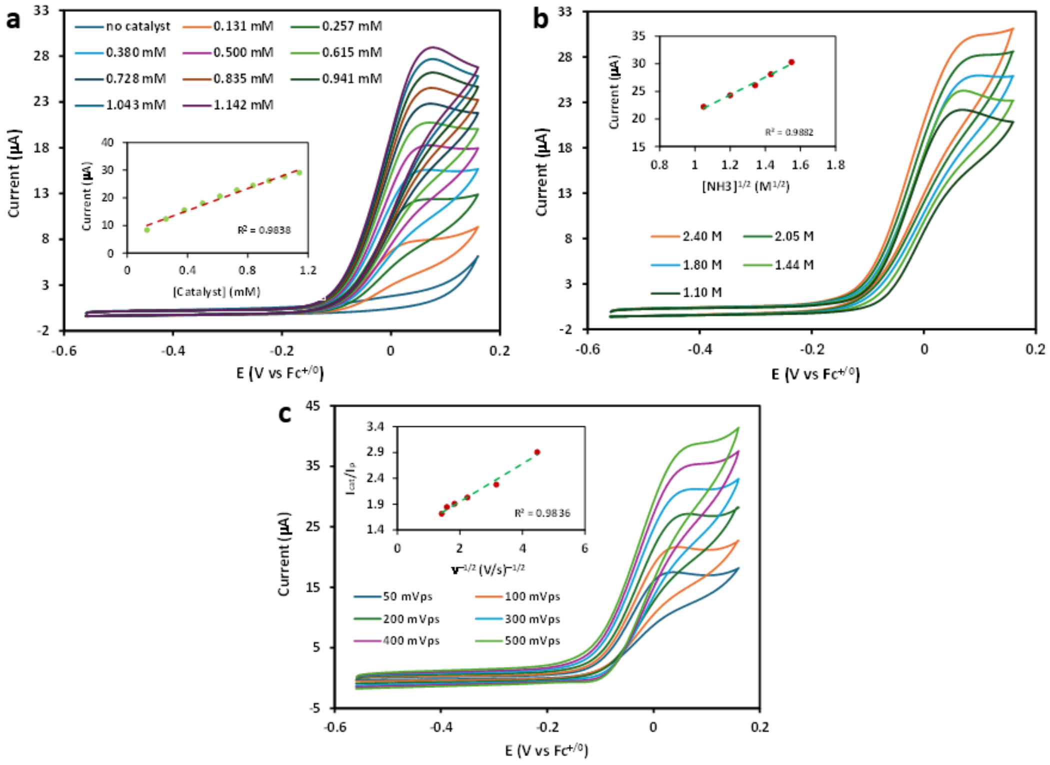

Figure S27. (**a**) Cyclic voltammograms of 0.131 – 1.142 mM **^{Py}Fc** (**4**) in 2.4 M NH_3 in DMSO; scan rate: 100 mVs^{-1}. (**b**) Cyclic voltammograms of 1.0 mM **^{Py}Fc** (**4**) in DMSO containing 1.10 – 2.40 M NH_3; scan rate: 100 mVs^{-1}. (**c**) Cyclic voltammograms of 1.0 mM **^{Py}Fc** (**4**) in DMSO containing 2.4 M NH_3 at scan rates of 50 – 500 mVs^{-1}. (inset) I_{cat}/I_p vs $v^{-1/2}$ plot. Conditions: 100 mM $[Bu_4N]PF_6$ supporting electrolyte with GC working, Pt counter and $Ag/AgNO_3$ reference electrodes.

9.4. Determination of NH_3/ND_3 Kinetic Isotope Effect for Ferrocene Complexes from Cyclic Voltammetry Experiments

9.4.1. Determination of NH_3/ND_3 Kinetic Isotope Effect for ^{Py2}Fc (1)

To determine the kinetic isotope effect k_H/k_D corresponding to the oxidation of NH_3 vs. ND_3, we carried out electrochemical measurements in presence of NH_3 and ND_3 with 1 mM **^{Py2}Fc** (**1**) solution in DMSO with 100 mM $[Bu_4N]PF_6$. From the CV data we have observed that the ammonia oxidation is first order in both $[NH_3]$ and $[ND_3]$ but the rate is much slower in case of ND_3. Comparing the slopes of I_{cat} vs [substrate] (NH_3 or ND_3) plot shown in Figure 28 we can determine the KIE for ammonia oxidation.

As I_{cat} variation is analogous to plotting $(k_{substrate})^{1/2}$ we can write that

$$KIE = \frac{k_{NH3}}{k_{ND3}} = \frac{slope_{NH3}}{slope_{ND3}}$$

$$= \frac{18.81(8)}{9.16(1)} = 2.05(1)$$

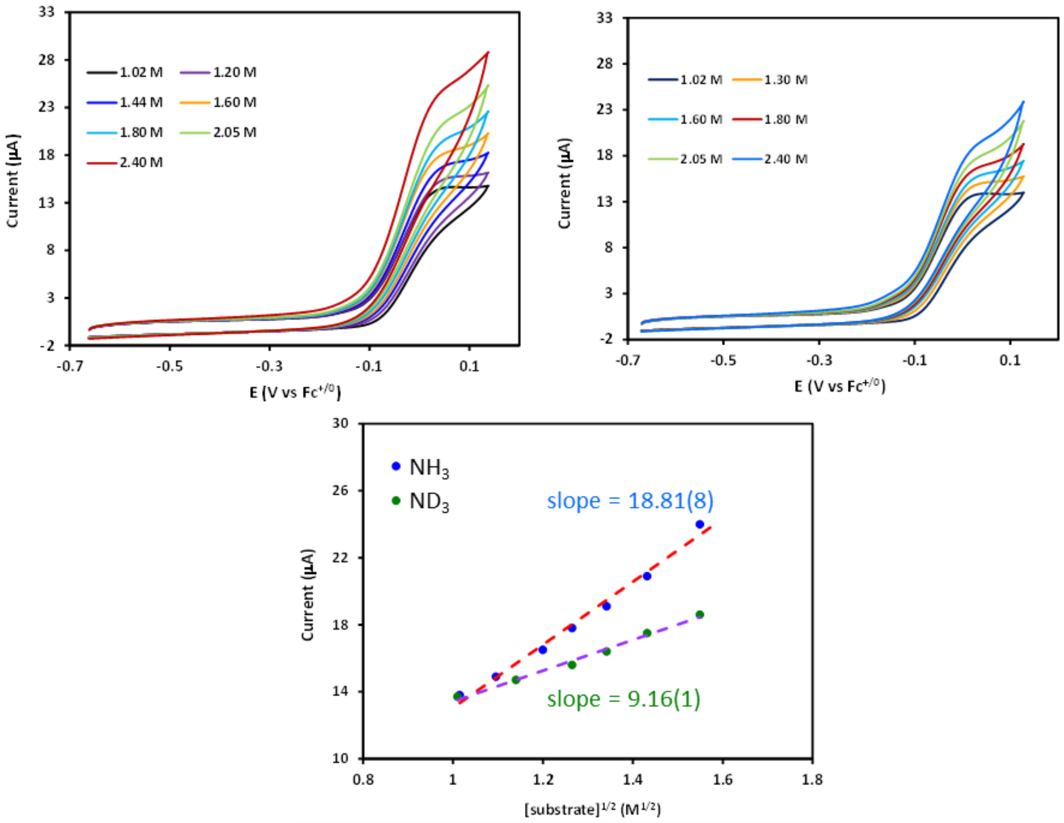

Figure S28. Cyclic voltammograms of 1.0 mM ^{py2}**Fc** (**1**) in DMSO in presence of various concentration of (a) NH_3 and (b) ND_3. (c) Concentration of NH_3 or ND_3 vs catalytic current plot to determine the k_H/k_D value. Conditions: 100 mM $[Bu_4N]PF_6$ supporting electrolyte; GC working, Pt counter and $Ag/AgNO_3$ reference electrodes are used, scan rate: 100 mVs^{-1}.

9.4.2. Determination of NH_3/ND_3 Kinetic Isotope Effect for $^{(Mepy)2}$Fc (2)

We can use KIE equation to find the k_H/k_D value for $^{(Mepy)2}$**Fc** (**2**), similar to the previous experiment.

$$KIE = \frac{k_{NH3}}{k_{ND3}} = \frac{slope_{NH3}}{slope_{ND3}}$$

$$= \frac{32.22(5)}{18.76(6)} = 1.72(1)$$

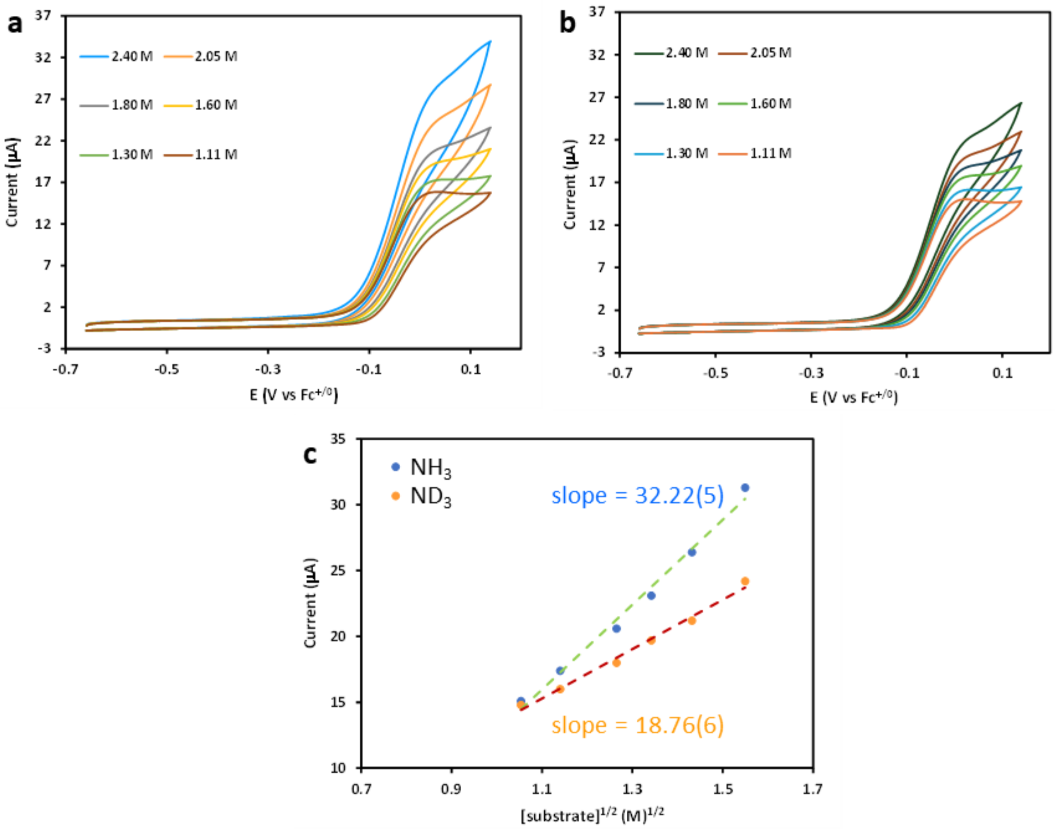

Figure S29. Cyclic voltammograms of 1.0 mM $^{\mathbf{(Mepy)2}}$**Fc** (**2**) in DMSO in presence of various concentration of (a) NH_3 and (b) ND_3. (c) Concentration of NH_3 or ND_3 vs catalytic current plot to determine the k_H/k_D value. Conditions: 100 mM $[Bu_4N]PF_6$ supporting electrolyte; GC working, Pt counter and $Ag/AgNO_3$ reference electrodes are used, scan rate: 100 mVs^{-1}.

9.4.3. Determination of NH_3/ND_3 Kinetic Isotope Effect for $^{\mathbf{(Me2OMepy)2}}$Fc (3)

Likewise, the k_H/k_D value for $^{\mathbf{(Me2OMepy)2}}$**Fc** (**3**) can be determined using KIE equation.

$$KIE = \frac{k_{NH3}}{k_{ND3}} = \frac{slope_{NH3}}{slope_{ND3}}$$

$$= \frac{13.81(6)}{11.68(2)} = 1.19(1)$$

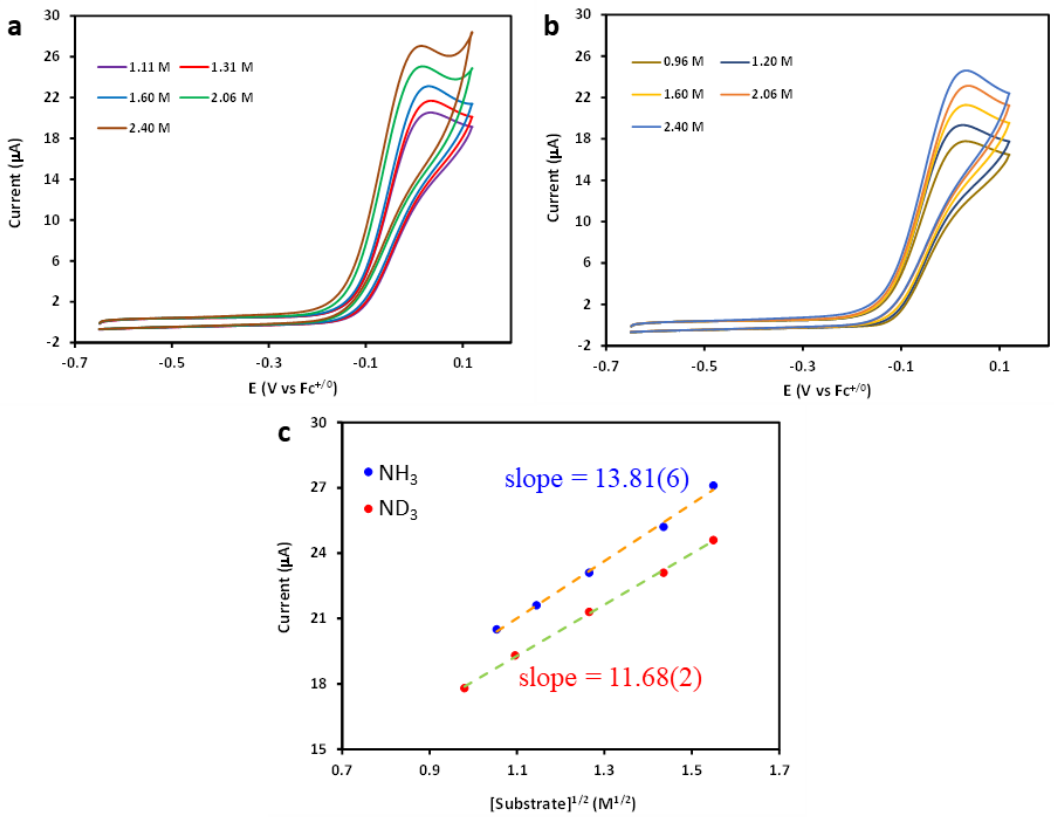

Figure S30. Cyclic voltammograms of 1.0 mM **$^{(Me2OMepy)2}$Fc** (**3**) in DMSO in presence of various concentration of (a) NH_3 and (b) ND_3. (c) Concentration of NH_3 or ND_3 vs catalytic current plot to determine the k_H/k_D value. Conditions: 100 mM $[Bu_4N]PF_6$ supporting electrolyte; GC working, Pt counter and Ag/$AgNO_3$ reference electrodes are used, scan rate: 100 mVs^{-1}.

Table S1. Comparison of electrochemistry data obtained in DMSO for complexes **1-4**.

**Complex**	**$E_{1/2}$ of $^{py2'}$Fc$^{+/0}$ redox couple (mV)**	**Diffusion coefficient (cm^2s^{-1})**	**Onset potential (mV vs. Fc$^{+/0}$)**	**TOF$_{max}$ (h^{-1})**	**Observed k_H/k_D**
**^{py2}Fc (1)**	–40	1.54×10^{-6}	–120	125	2.05(1)
**^{Mepy}Fc (2)**	–68	2.11×10^{-6}	–140	223	1.72(1)
**$^{(Me2OMepy)2}$Fc (3)**	–100	0.77×10^{-6}	–170	560	1.19(1)
**^{Py}Fc (4)**	–25	2.44×10^{-6}	–100	117	–

10. Controlled Potential Electrolysis (CPE)

Controlled potential electrolysis of ammonia in DMSO in the presence of 1.0 mM **^{py2}Fc** (**1**), **$^{(Mepy)2}$Fc** (**2**), and **$^{(Me2OMpy)2}$Fc** (**3**) with 100 mM $[Bu_4N]PF_6$ were performed using a custom-made gas tight electrolysis cell equipped with four necks to accommodate three detachable electrodes and one sampling port (Figure S31).

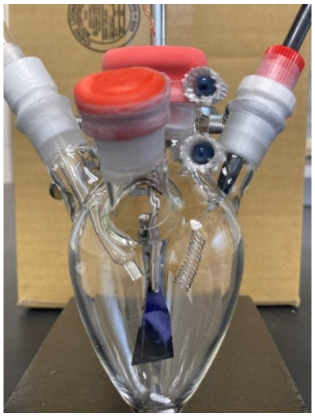

Figure S31. Pictorial representation of an electrolysis cell used for CPE experiments. The empty cell is presented here for better visibility. Glassy carbon plate working, Pt mesh counter and, custom made $Ag/AgNO_3$ reference electrodes are used. For smaller volumes, longer electrodes were used.

For CPE experiments, the 4-neck electrolysis cell equipped with three electrodes (GC plate, A = 1.0 cm^2; Pt mess; and custom $Ag/AgNO_3$) was sealed carefully and sparged under a flow of argon for 2 h. The cell was disconnected from the argon line and was kept closed for 2 h, during which 100 μL samples of the cell headspace were injected to the GC every 1 h to check for leaks. After that 1.0 mM **^{py2}Fc** (**1**) solution containing 2.4 M NH_3 (30 mL) was transferred to the cell by injecting through the sampling port. Then CPE was performed at a constant potential of 100 mV vs $Fc^{+/0}$ for 3 h (Figure S35). After CPE experiment, 100 μL of the headspace was injected to the GC.

To evaluate the stability of the catalyst and analyze the liquid phase, we conducted a CPE experiment for 2 h using complex **1** (2.0 mM) in NH_3 saturated DMSO-d_6, with 100 mM $[Bu_4N]PF_6$ as the supporting electrolyte and 1 equivalent of 1,2,4,5-tetrachlorobenzene as an internal standard. The reaction mixture was analyzed by ^{1}H NMR before and after electrolysis. The ^{1}H NMR spectra revealed that only 6% of the complex decomposed after 2 h, with no additional peaks corresponding to other species observed.

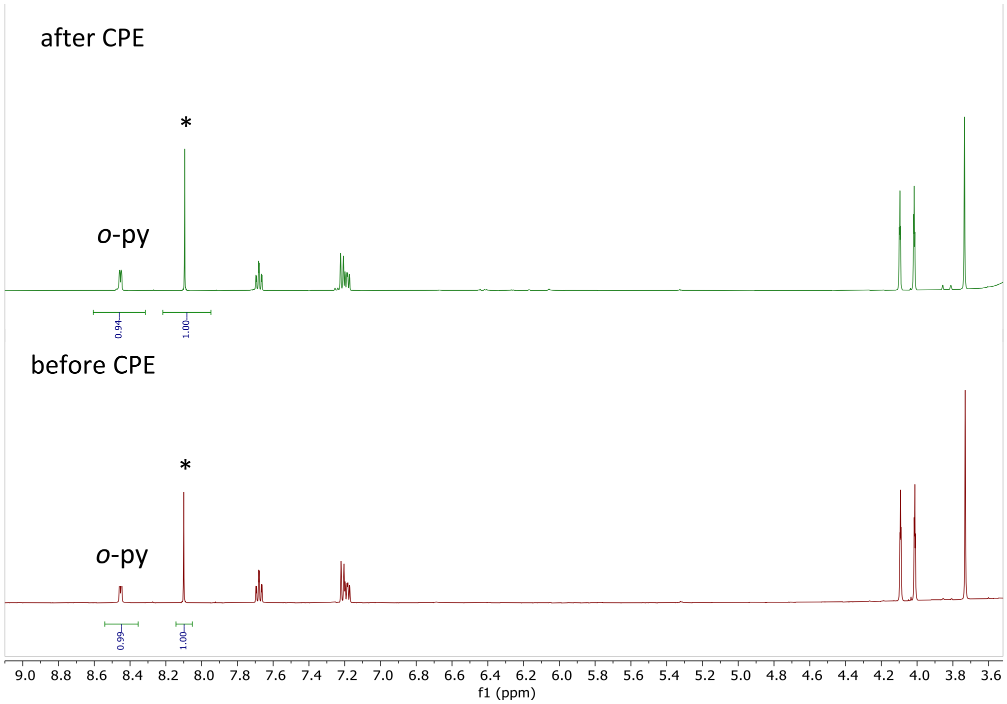

Figure S32. Overlay of ^{1}H NMR spectra (500 MHz, DMSO-d_6, 22 °C) of complex **1** before (maroon, below) and after (green, above) CPE for 2 h in NH_3 saturated DMSO-d_6. (* denotes 1,2,4,5-tetrachlorobenzene resonance). The region below 3.5 ppm is not shown due to the overwhelming concentration of the electrolyte $[Bu_4N]PF_6$ (100 mM) relative to the catalyst (2.0 mM).

11. Rinse Test

To determine if the observed electrocatalysis is due to a decay product of the catalyst deposited on the electrode surface, we performed a rinse test after constant potential electrolysis (CPE). Following the CPE experiment with a glassy carbon electrode (A = 0.07 cm²) for 2 hours, the GC electrode was rinsed with DMSO and then placed in a new cell containing 1.2 M NH_3 and 100 mM $[Bu_4N]PF_6$ in DMSO without the $^{\mathbf{py2}}$**Fc** catalyst. No electrocatalytic current was observed at 0.1 V vs $Fc^{+/0}$, indicating that the electrocatalytic activity in the CPE experiment originates from the soluble complex **1** (Figure S33).

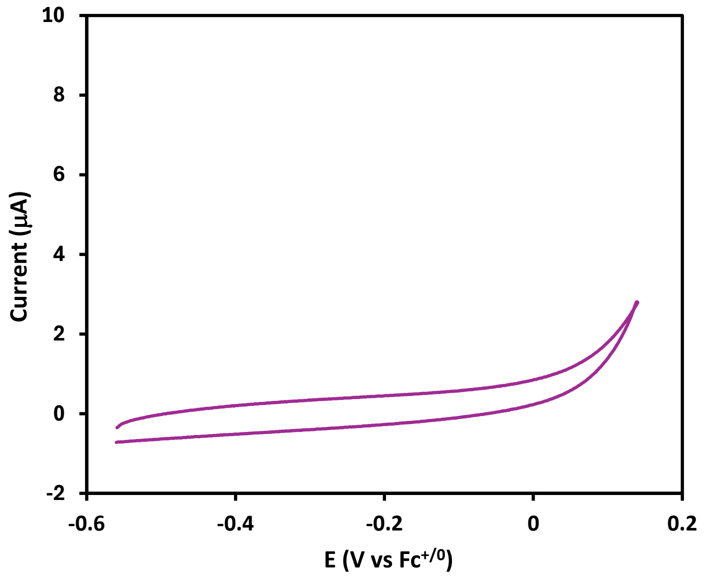

Figure S33. (Rinse Test) Cyclic voltammetry was performed after 2 hours of constant potential electrolysis (CPE) using the same glassy carbon electrode (A = 0.07 cm^2). The electrode was rinsed with DMSO and placed in a new cell containing 1.2 M NH_3 and 100 mM $[Bu_4N]PF_6$ in DMSO. Conditions: 100 mV/s scan rate, with a platinum counter electrode and Ag/$AgNO_3$ reference electrode.

12. Gas Chromatography (GC) to Detect N_2 and H_2

Gas quantification was conducted with a gas chromatograph (Agilent 7820A) equipped with an Agilent PLOT/U capillary column (Agilent 19095P-UO4PT 30 m × 350 µm × 20 µm), a CP-molsieve 5Å capillary column (Agilent CP7539 50 m × 530 µm × 50 µm) and a thermal conductivity detector (TCD). Argon was used as the carrier gas. The PLOT/U capillary column is connected to the injector and exits to a pneumatic valve that switches between the CP-molsieve 5Å capillary column and the detector. Ten min after injection, the pneumatic valve switches to isolate the faster eluting, volatile diatomic gases in the molsieve column, allowing the slower eluting volatiles DMSO and NH_3 to pass from the PLOT/U column directly to the detector. At ca. 17 min, the valve resets and the diatomics in the molsieve column then elute to the detector. 100 µL volumes of headspace mixture were manually injected to the GC using a gastight syringe (Hamilton, USA). Evolution of N_2 and H_2 was quantified based on an independent calibration obtained separately for each gas by direct injection of known volumes of hydrogen and nitrogen (Airgas, USA) to the GC as shown in Figure S34. The moles of N_2 and H_2 are calculated using the ideal gas law with T = 293 K and P = 1 atm. The data points for 0 mol N_2 and H_2 in Figure S34 correspond to 100 µL injections of argon gas, corrected for residual N_2 in the argon gas used during CPE. The gas chromatography measurement of N_2 and H_2 was done nearly analogously as reported by Hamann, Smith, and colleagues in a 2019 report in PNAS.[6]

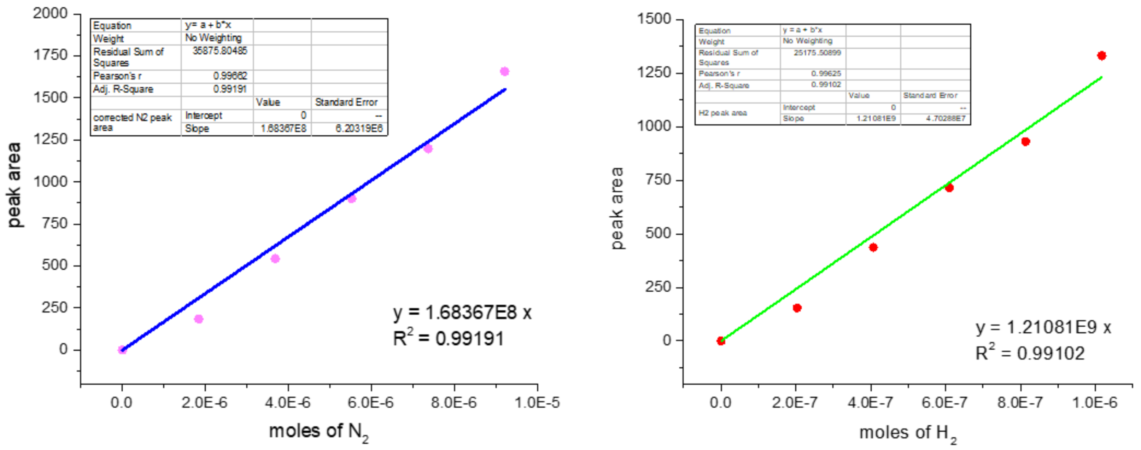

Figure S34. Gas chromatography calibration lines obtained for (left) N_2 and (right) H_2.

13. Faradaic Yield (FY) Calculation from CPE Experiments

FY is determined by the equation:

FY (%) = 100 × 6 × amount of N_2 produced (moles)/ (amount of charge consumed during CPE/F)

= 100 × 6 × F × amount of N_2 produced (moles)/ amount of charge consumed during CPE

During constant potential electrolysis (CPE) at 0.1 V vs $Fc^{+/0}$ total charge consumed 2.53 C in 182 min and amount of N_2 produced 3.32×10^{-6} moles.

Therefore, the Faradaic yield for N_2 production is 75.9%.

Similarly, amount of H_2 produced at the same time is 9.04×10^{-6} moles.

FY (%) = 100 × 2 × F × amount of H_2 produced (moles)/ amount of charge consumed during CPE

= 69% faradaic yield for H_2 production.

The lower observed faradaic yield measured for H_2 vs N_2 likely results from the greater effusivity of H_2 vs N_2 that may result in a lower measured amount of H_2 gas vs N_2 gas produced in the CPE experiment.

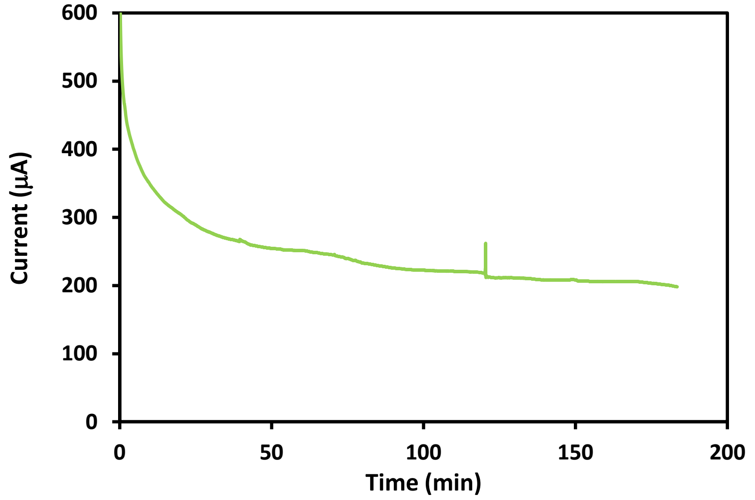

Figure S35. Current passed during controlled potential electrolysis. ^{py2}**Fc** = 1.0 mM, [NH_3] = 2.4 M, 100 mM [Bu_4N]PF_6 in dry DMSO. The amount of charge (*q*, in coulombs) passed 3.53 C in 182 min calculated from the equation ($q = i \times t$).

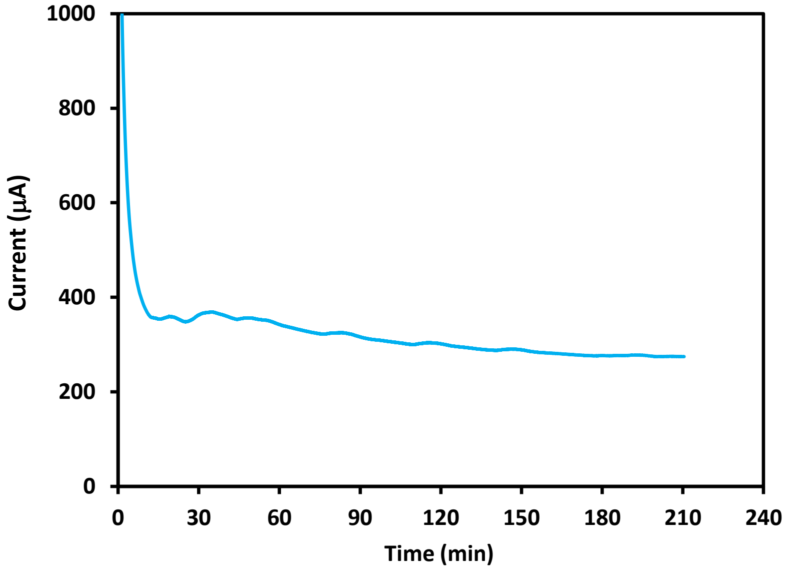

Figure S36. Current passed during controlled potential electrolysis. $^{(Mepy)2}$**Fc** = 1.0 mM, [NH_3] = 2.4 M, 100 mM [Bu_4N]PF_6 in dry DMSO. The amount of charge (*q*, in coulombs) passed 3.78 C in 210 min calculated from the equation ($q = i \times t$).

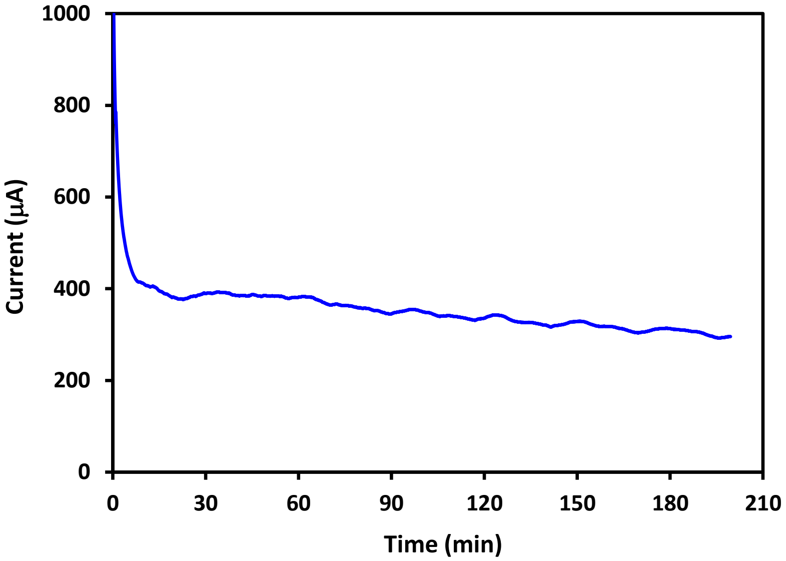

Figure S37. Current passed during controlled potential electrolysis. **$^{(Me2OMepy)2}$Fc** = 1.0 mM, [NH_3] = 2.4 M, 100 mM [Bu_4N]PF_6 in dry DMSO. The amount of charge (*q*, in coulombs) passed 3.96 C in 200 min calculated from the equation ($q = i \times t$).

Table S2. Comparison of Faradaic Yields for N_2 and H_2 from Complexes **1-3**.

Catalyst	CPE time (min)	Charge consumed (Coulomb)	FY of N_2 (%)	FY of H_2 (%)
**^{Py2}Fc (1)**	182	2.53	75.9	69.0
**$^{(Mepy)2}$Fc (2)**	210	3.78	79.6	72.9
**$^{(Me2OMe)2}$Fc (3)**	200	3.96	75.1	70.2

14. Determination of Turnover Number (TON) for Complex 1

$$TON = \frac{moles\ of\ N_2\ produced}{moles\ of\ catalyst\ used}$$

A constant potential electrolysis (CPE) experiment was conducted in a two-compartment electrochemical cell, where the counter electrode was separated by a frit from the glassy carbon plate working electrode (A = 2.2 cm^2). For the CPE experiment, 3.2 mg of the catalyst (8.68×10^{-6} moles) was used, and the total charge consumed was 34.56 C.

After 19.2 hours of CPE experiment, the amounts of N_2 produced were 4.32×10^{-5} moles. The overall Faradaic yield was determined to be 72.3%.

Therefore, TON after 19.2 h of CFE experiment = (4.32×10^{-5}) / (8.68×10^{-6}) = 4.97.

15. Crystallographic Details

X-ray data for single crystal of compounds ^{py2}**Fc** (**1**) (CCDC 2335846), $^{(Mepy)2}$**Fc** (**2**) (CCDC 2352837), $^{(Me2OMepy)2}$**Fc** (**3**) (CCDC 2352507),), and ^{py}**Fc** (**4**) (CCDC 2340164) were collected in *Michigan State University*. A single crystal was selected and mounted on a nylon loop with paratone oil on a XtaLAB Synergy, Dualflex, HyPix diffractometer. The crystal was kept at a steady T = 99.99(10) K during data collection. MSU Data were measured using ω scans using Cu K_a radiation (micro-focus sealed X-ray tube, 50 kV, 1 mA). The total number of runs and images was based on the strategy calculation from the program CrysAlisPro 1.171.41.122a (Rigaku OD, 2021). The achieved resolution was Q = 79.410. The structure was solved with the **ShelXS**[7] (Sheldrick, 2008) solution program using direct methods and by using **Olex2** 1.5 (Dolomanov et al., 2009) as the graphical interface. The model was refined with **ShelXL** 2018/3 (Sheldrick, 2015)[7] using full matrix least squares minimization on $\boldsymbol{F^2}$. Further comments on disorder models:

$^{(Me2OMepy)2}$**Fc** (**3**) (CCDC 2352507): This crystal showed to be a whole molecule disorder in the modeling of the structure's Fe iron was allowed to remain on the symmetry site since attempts to build a disordered model with it in two spots failed to yield a reasonable model. The rest of the molecule was modelled in two parts using the SAME command in SHEXL and restraining the carbon thermals all together to prevent correlation to cause refinement problems with the models. No other constraints or restraints were used. The two molecules were allowed to refine joint occupancy and the refined occupancy ratio was refined to 0.73:0.27. All hydrogen atoms were located in their calculated positions.

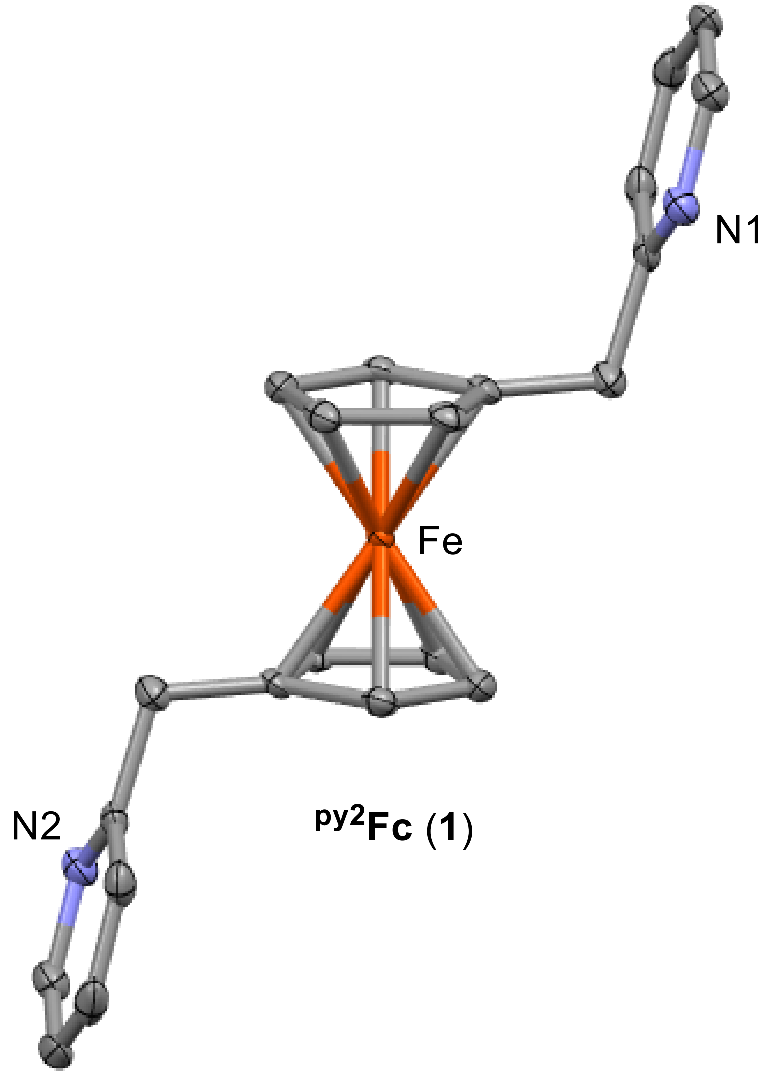

^{py2}Fc (1)

Figure S38. X-ray structure of **^{py2}Fc** (**1**) (CCDC 2335846). The thermal ellipsoid plots are drawn at 50% probability level. Hydrogen atoms are omitted for clarity. Fe-centroid distance 1.644 Å.

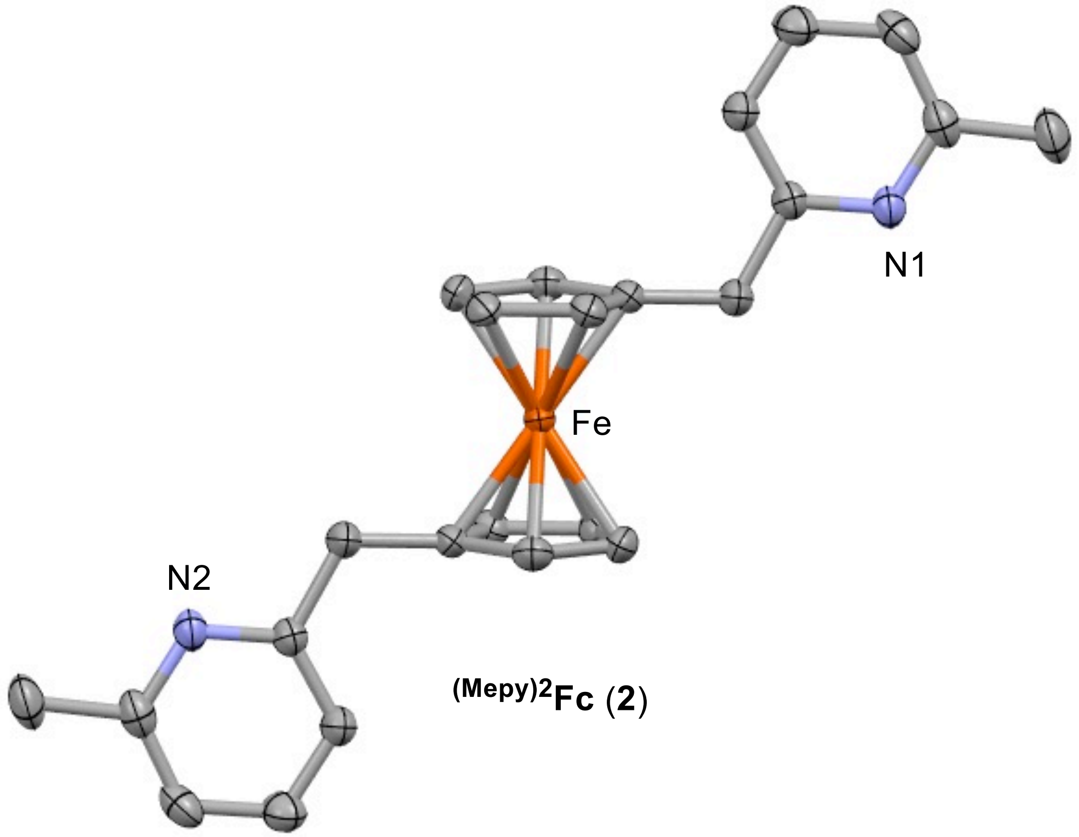

$^{(Mepy)2}$**Fc** (**2**)

Figure S39. X-ray structure of $^{(Mepy)2}$**Fc** (**2**) (CCDC 2352837). The thermal ellipsoid plots are drawn at 50% probability level. Hydrogen atoms are omitted for clarity. Fe-centroid distance 1.651 Å.

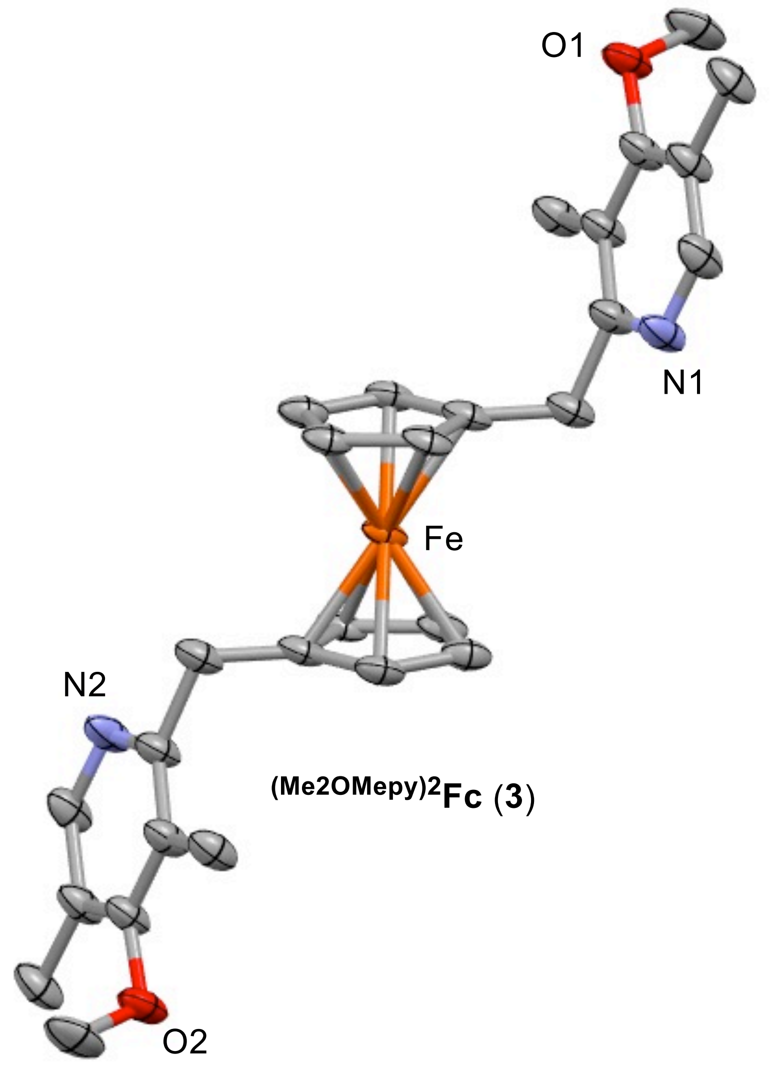

Figure S40. X-ray structure of $^{(Me2OMepy)2}$**Fc** (CCDC 2352507). The thermal ellipsoid plots are drawn at 50% probability level. Hydrogen atoms are omitted for clarity. Fe-centroid distance 1.662 Å.

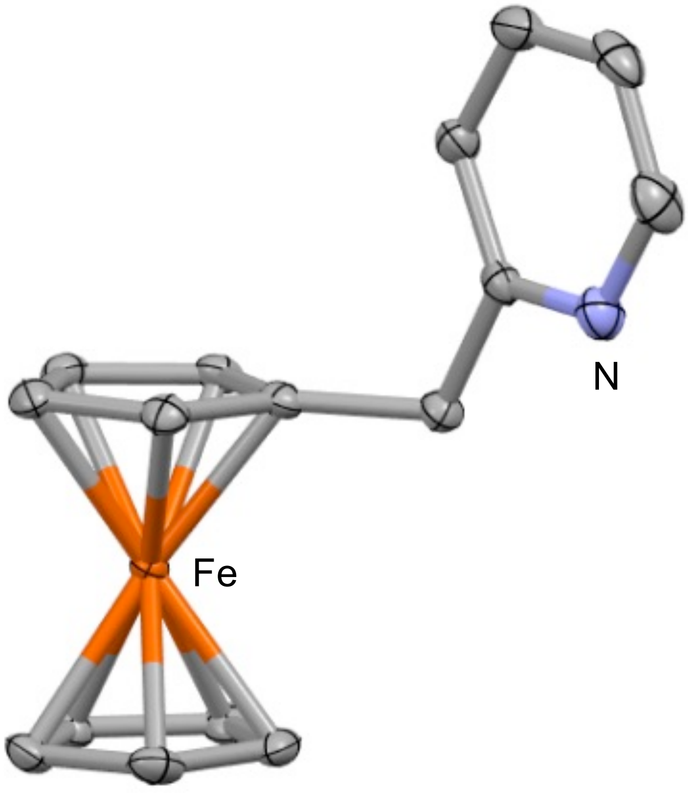

^{py}Fc (6)

Figure S41. X-ray structure of **^{py}Fc** (CCDC 2340164). The thermal ellipsoid plots are drawn at 50% probability level. Hydrogen atoms are omitted for clarity. Fe-Cp centroid distance 1.645 Å and Fe-^{py}Cp centroid distance 1.639 Å.

16.1. Computational Results

Gaussian 16[7] was used to optimize the structures, calculate single point geometries, and determine vibrational frequencies as well as reaction free energies. Visualization and structural analyses were performed using Chemcraft 1.8[8] and CCDC-Mercury.[8] Nine complexes were studied: ^{py2}**Fc(II)** (**1**), NH_3···^{py2}**Fc**, {^{py2}**Fc**}$^+$, {NH_3···^{py2}**Fc**}$^+$, {NH_2···H^{py2}**Fc**}$^+$, {H^{py2}**Fc**}$^+$, $^{(Mepy)2}$**Fc(II)** (**2**), NH_3···$^{(Mepy)2}$**Fc**, {$^{(Mepy)2}$**Fc**}$^+$, {NH_3···$^{(Mepy)2}$**Fc**}$^+$, {NH_2···H$^{(Mepy)2}$**Fc**}$^+$, {H$^{(Mepy)2}$**Fc**}$^+$, $^{(Me2OMepy)2}$**Fc(II)** (**3**), NH_3···$^{(Me2OMepy)2}$**Fc**, {$^{(Me2OMepy)2}$**Fc**}$^+$, {NH_3···$^{(Me2OMepy)2}$**Fc**}$^+$, {NH_2···H$^{(Me2OMepy)2}$**Fc**}$^+$, {H$^{(Me2OMepy)2}$**Fc**}$^+$. Sixteen small molecules (pyridine (py), py…NH_3, {pyH…H_2N}$^+$, pyH$^+$, 2-methylpyridine (2-Me-py), 2-Me-py…NH_3, {2-Me-pyH…H_2N}$^+$, {2-Me-pyH}$^+$, 4-methoxy-3,5-dimethylpyridine (OMeMe$_2$py), OMeMe$_2$py…NH_3, {OMeMe$_2$pyH…H_2N}$^+$, {OMeMe$_2$pyH}$^+$, NH_3, N_2H_4, NH_4^+, and [NH_4•NH_3]$^+$) were also calculated to analyze the thermodynamics of the reactions between the complexes. The B3LYP[9] functional in conjunction with the 6-311+G(d) basis set (5d 7f spherical harmonics)[10-13] was used for optimization of all geometries. At the B3LYP/6-311+G(d,p) stationary points, single point energies were calculated using B3LYP with the 6-311++G(d,p) basis set, in DMSO using the implicit SMD solvent model[10-16] and adding dispersion corrections with the keyword, empirical dispersion = GD3BJ in Gaussian.[16-23]

Table S3. Gibbs free energies (UB3LYP+GD3BJ/6-311++G(d,p)/SMD-DMSO//B3LYP/6-311+G(d,p)/gas) at 298.15 K of the computed molecules. Fc = ferrocene.

Compound	Charge	Multiplicity	Gibbs Free Energy (Hartree)
Py (**1py**)	0	1	-155820.3253
**1py**…NH_3	0	1	-191314.5154
{**1py**H…H_2N}1^+	1	2	-191178.8554
**1py**H^+	1	1	-156099.7182
2-Me-py (**2py**)	0	1	-180488.6546
**2py**…NH_3	0	1	-215982.9744
{**2py**H…H_2N}$^+$	1	2	-215849.2011
{**2py**H}$^+$	1	1	-180771.0366
$OMeMe_2py$ (**3py**)	0	1	-277021.3853
**3py**…NH_3	0	1	-312516.0006
{**3py**H…H_2N}$^+$	1	2	-312384.3934
{**3py**H}$^+$	1	1	-277305.75
NH_3	0	1	-35501.18989
N_2H_4	0	1	-70212.71241
NH_4^+	1	1	-35782.94242
$[NH_4•NH_3]^+$	1	1	-71283.7719
$^{\mathbf{py2}}$**Fc(II)**	0	1	-1395375.551
$NH_3^{\cdots \mathbf{py2}}$**Fc**	0	1	-1430870.942
{$NH_3^{\cdots \mathbf{py2}}$**Fc**}$^+$	1	2	-1430751.022
{$NH_2{\cdots}H^{\mathbf{py2}}$**Fc**}$^+$	1	2	-1430732.741
{$H^{\mathbf{py2}}$**Fc**}$^+$	1	1	-1395656.052
{$NH_3^{\cdots \mathbf{(Mepy)2}}$**Fc**}$^+$	1	2	-1480088.357
{$NH_2{\cdots}H^{\mathbf{(Mepy)2}}$**Fc**}$^+$	1	2	-1480071.893
{$NH_3^{\cdots \mathbf{(Me2OMepy)2}}$**Fc**}$^+$	1	2	-1673151.767
{$NH_2{\cdots}H^{\mathbf{(Me2OMepy)2}}$**Fc**}$^+$	1	2	-1673137.829

Table S4. Calculated Enthalpies of hydrogen bonding interactions between NH_3 and pyridine substituted ferrocenes **1** – **3**.

**Reaction**	**ΔH kcal/mol**
^{py2}Fc (**1**) + NH_3 → $H_2NH^{\cdots py2}Fc$	−1.94
$^{(Mepy)2}Fc$ (**2**) + NH_3 → $H_2NH^{\cdots (Mepy)2}Fc$	−1.97
$^{(Me2OMepy)2}Fc$ (**3**) + NH_3 → $H_2NH^{\cdots (Me2OMepy)2}Fc$	−2.07

Table S5. Calculated Gibbs free energies of hydrogen bonding interactions between NH_3 and pyridine substituted ferrocenes **1** – **3**.

**Reaction**	**ΔG kcal/mol**
^{py2}Fc (**1**) + NH_3 → $H_2NH^{\cdots py2}Fc$	6.3
$^{(Mepy)2}Fc$ (**2**) + NH_3 → $H_2NH^{\cdots (Mepy)2}Fc$	6.0
$^{(Me2OMepy)2}Fc$ (**3**) + NH_3 → $H_2NH^{\cdots (Me2OMepy)2}Fc$	3.8

Table S6. Calculated Gibbs free energies of hydrogen bonding interactions between NH_3 and pyridine substituted ferrocenes in oxidized state.

**Reaction**	**ΔG kcal/mol**
$^{py2}Fc^+$ (**1**$^+$) + NH_3 → $\{H_2NH^{\cdots py2}Fc\}^+$	7.8
$^{(Mepy)2}Fc^+$ (**2**$^+$) + NH_3 → $\{H_2NH^{\cdots (Mepy)2}Fc\}^+$	7.6
$^{(Me2OMepy)2}Fc^+$ (**3**$^+$) + NH_3 → $\{H_2NH^{\cdots (Me2OMepy)2}Fc\}^+$	6.4

Table S7. Calculated Gibbs free energies of PCET in pyridine substituted ferrocenes in oxidized state.

**Reaction**	**ΔG kcal/mol**
$\{H_2NH^{\cdots py2}Fc\}^+$ → $\{H_2N^{\cdots}H^{py2}Fc\}^+$	18.2
$\{H_2NH^{\cdots (Mepy)2}Fc\}^+$ → $\{H_2N^{\cdots}H^{(Mepy)2}Fc\}^+$	16.5
$\{H_2NH^{\cdots (Me2OMepy)2}Fc\}^+$ → $\{H_2N^{\cdots}H^{(Me2OMepy)2}Fc\}^+$	13.9

Table S8. Calculated Gibbs free energies for N–N coupling to hydrazine formation.

Reaction	**Δ*G* kcal/mol**
$\{H_2N{\cdots}H^{py2}Fc\}^+ \rightarrow \{H^{py2}Fc\}^+ + ½\ N_2H_4$	–29.8
$\{H_2N{\cdots}H^{(Mepy)2}Fc\}^+ \rightarrow \{H^{(Mepy)2}Fc\}^+ + ½\ N_2H_4$	–30.2
$\{H_2N{\cdots}H^{(Me2OMepy)2}Fc\}^+ \rightarrow \{H^{(Me2OMepy)2}Fc\}^+ + ½\ N_2H_4$	–32.2

Table S9. Calculated Gibbs free energies for deprotonation of pyridinium to respective pyridine.

Reaction	**Δ*G* kcal/mol**
$\{H^{py2}Fc\}^+ + NH_3 \rightarrow {}^{py2}Fc + NH_4^+$	-1.14
$\{H^{(Mepy)2}Fc\}^+ + NH_3 \rightarrow {}^{(Mepy)2}Fc + NH_4^+$	2.31
$\{H^{(Me2OMepy)2}Fc\}^+ + NH_3 \rightarrow {}^{(Me2OMepy)2}Fc + NH_4^+$	3.45

Table S10. Calculated Gibbs free energies of hydrogen bonding interactions between NH_3 or ND_3 with substituted pyridines **1py** – **3py** along with equilibrium isotope effects EIE (K_H/K_D).

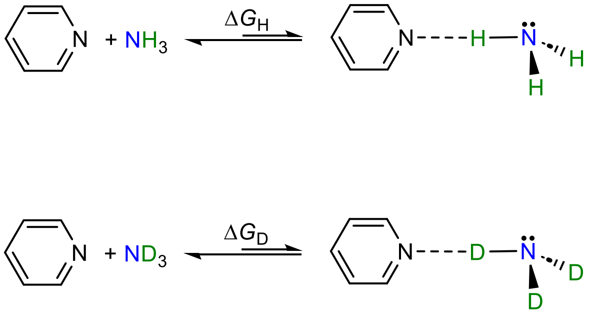

Molecule	ΔG_H (kcal/mol)	ΔG_D (kcal/mol)	EIE (K_H/K_D)
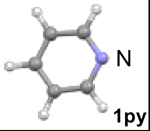 **1py**	6.999	6.967	0.94
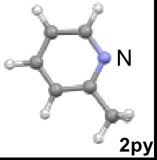 **2py**	6.870	6.832	0.94
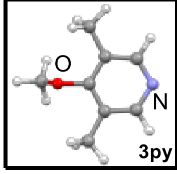 **3py**	6.574	6.518	0.91

Table S11. Gibbs free energies (UB3LYP+GD3BJ/6-311++G(d,p)/SMD-DMSO//B3LYP/6-311+G(d,p)/gas) at 298.15 K of the computed molecules. Fc = ferrocene. PCET = proton coupled electron transfer.

Molecule	ΔG_1 (kcal/mol)	ΔG_2 (kcal/mol)	ΔG_3 (kcal/mol)	ΔG_{Fc} (kcal/mol)	$\Delta G_{overall}$ (kcal/mol)
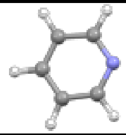 **1py**	7.0	135.6	-27.2	19.6	-0.6
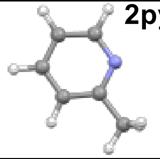 **2py**	6.8	133.7	-28.1	17.7	-3.6
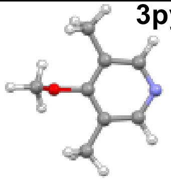 **3py**	6.5	131.6	-27.7	15.6	-5.6

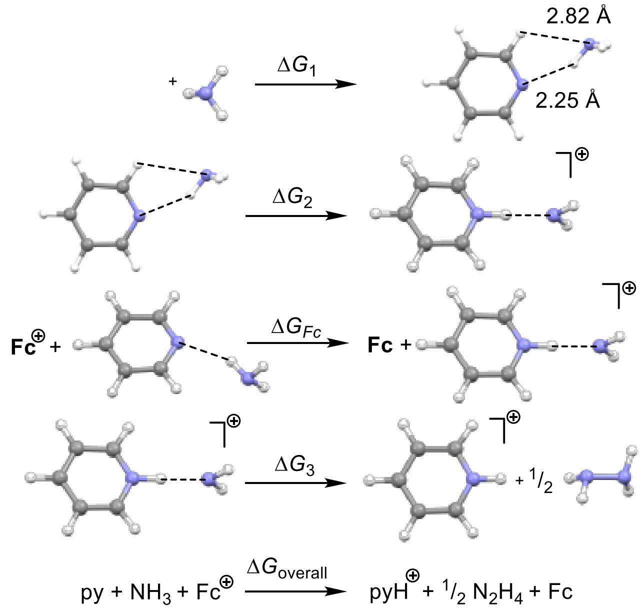

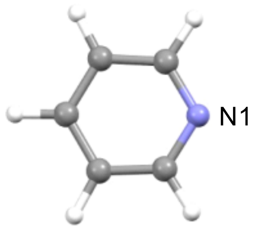

Figure S42. Geometry optimized structure of pyridine (**1py**) at B3LYP/6-311+G(d,p) level of theory with charge = 0, multiplicity = 1.

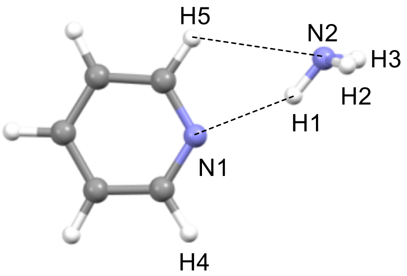

Figure S43. Geometry optimized structure of py···H_3N at B3LYP/6-311+G(d,p) level of theory with charge = 0, multiplicity = 1. Selected calculated bond distances (Å): N1–H1 2.256, N2–H5 2.822.

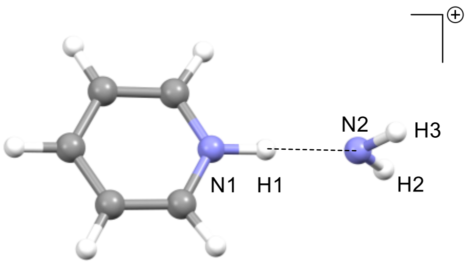

Figure S44. Geometry optimized structure of $\{pyH\cdots NH_2\}^+$ at B3LYP/6-311+G(d,p) level of theory with charge = 1, multiplicity = 2. Selected calculated bond distances (Å): N1–H1 1.051, N2–H1 1.793, N2–H2 1.026.

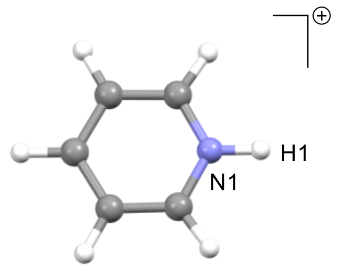

Figure S45. Geometry optimized structure of pyH^+ at B3LYP/6-311+G(d,p) level of theory with charge = 1, multiplicity = 1. Selected calculated bond distances (Å): N1–H1 1.016.

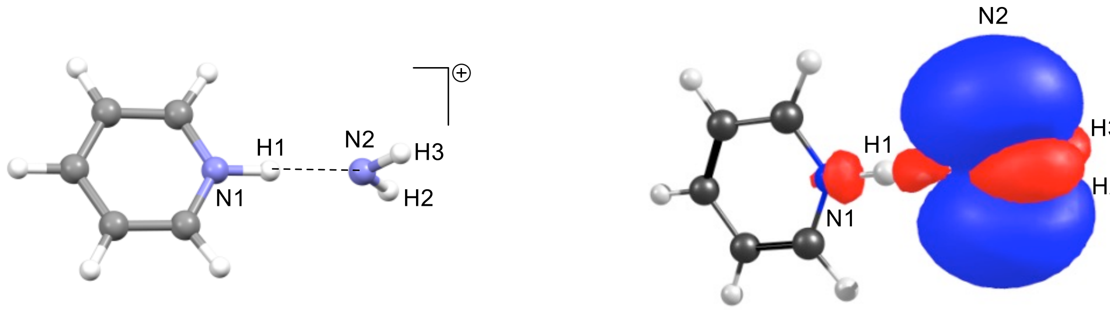

Figure S46. Spin density plot of $\{pyH^{\cdots}NH_2\}^+$. Isosurface value = 0.0005. Spin density: N2 102%.

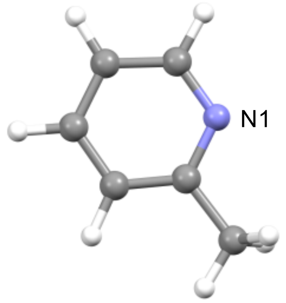

Figure S47. Geometry optimized structure of 2-methyl-pyridine (**2py**) at B3LYP/6-311+G(d,p) level of theory with charge = 0, multiplicity = 1.

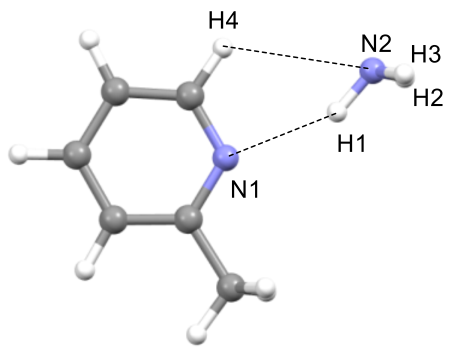

Figure S48. Geometry optimized structure of 2-Me-py···H_3N at B3LYP/6-311+G(d,p) level of theory with charge = 0, multiplicity = 1. Selected calculated bond distances (Å): N1–H1 2.254, N2–H4 2.836.

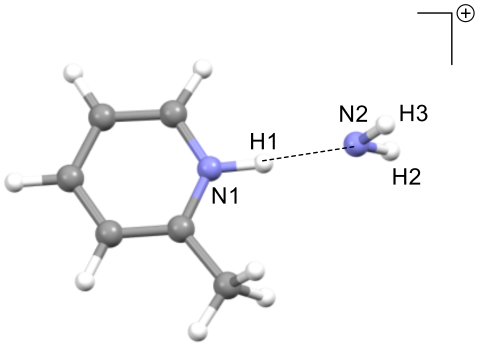

Figure S49. Geometry optimized structure of $\{2\text{-Me-pyH}\cdots NH_2\}^+$ at B3LYP/6- 311+G(d,p) level of theory with charge = 1, multiplicity = 2. Selected calculated bond distances (Å): N1–H1 1.045, N2–H1 1.841, N2–H2 1.026.

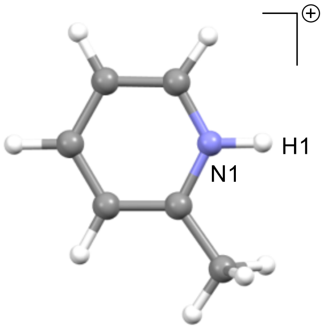

Figure S50. Geometry optimized structure of $\{2\text{-Me-pyH}\}^+$ at B3LYP/6-311+G(d,p) level of theory with charge = 1, multiplicity = 1. Selected calculated bond distances (Å): N1–H1 1.016.

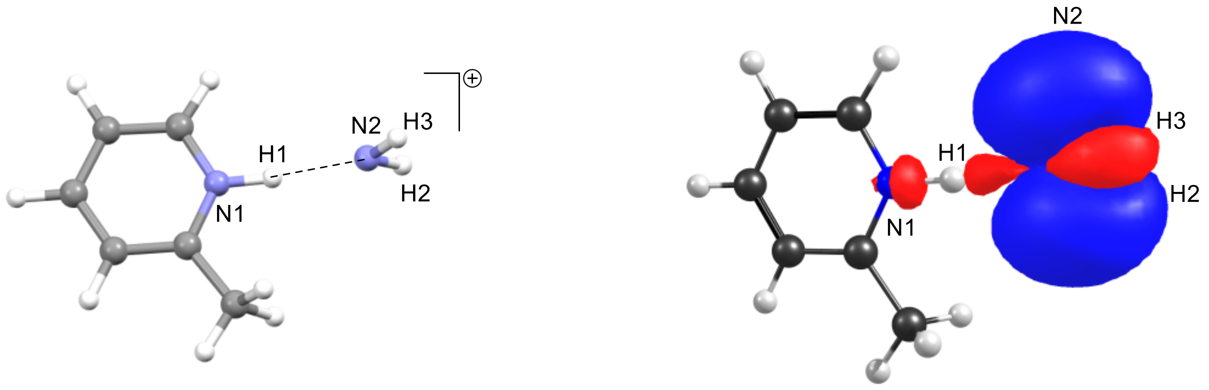

Figure S51. Spin density plot of {2-Me-pyH···NH_2}$^+$. Isosurface value = 0.0005. Spin density: N2 109%.

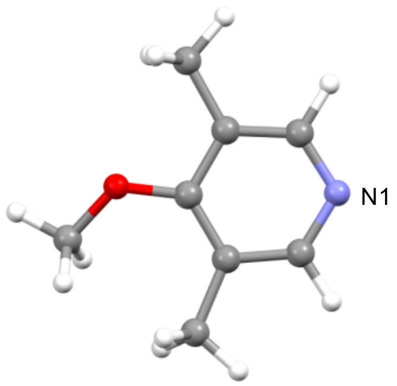

Figure S52. Geometry optimized structure of Me_2OMe-pyridine (**3py**) at B3LYP/6-311+G(d,p) level of theory with charge = 0, multiplicity = 1.

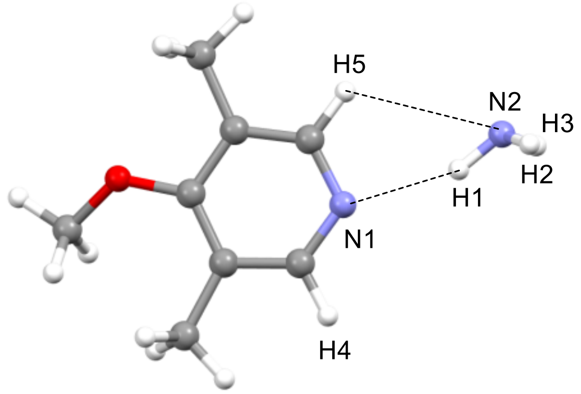

Figure S53. Geometry optimized structure of $Me_2OMepy{\cdots}H_3N$ at B3LYP/6-311+G(d,p) level of theory with charge = 0, multiplicity = 1. Selected calculated bond distances (Å): N1–H1 2.219, N2–H5 2.521.

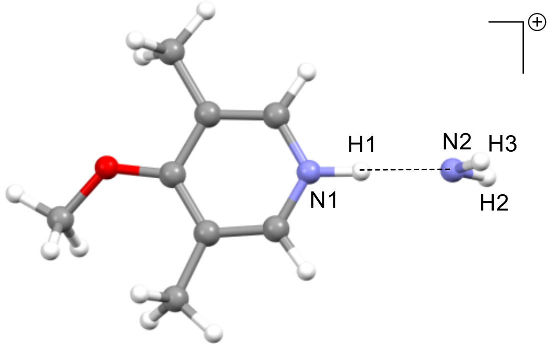

Figure S54. Geometry optimized structure of $\{Me_2OMepyH{\cdots}NH_2\}^+$ at B3LYP/6-311+G(d,p) level of theory with charge = 1, multiplicity = 2. Selected calculated bond distances (Å): N1–H1 1.041, N2–H1 1.846, N2–H2 1.026.

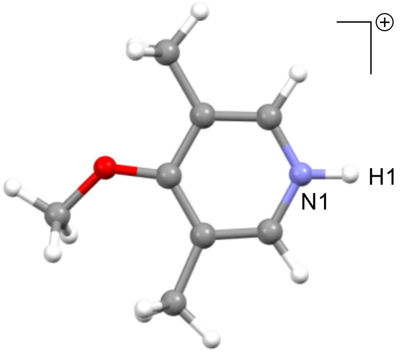

Figure S55. Geometry optimized structure of $\{Me_2OMepyH\}^+$ at B3LYP/6-311+G(d,p) level of theory with charge = 1, multiplicity = 1. Selected calculated bond distances (Å): N1–H1 1.013.

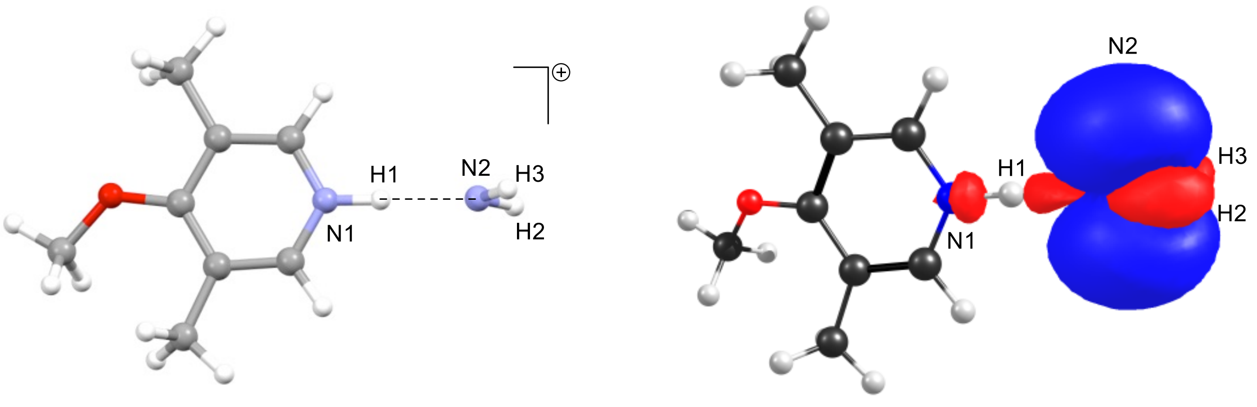

Figure S56. Spin density plot of $\{Me_2OMepyH^{\cdots}NH_2\}^+$. Isosurface value = 0.0005. Spin density: N2 109%.

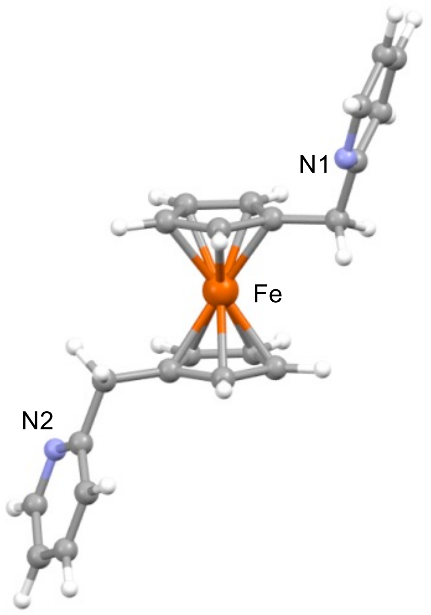

Figure S57. Geometry optimized structure of **^{py2}Fc** (**1**) at B3LYP/6-311+G(d,p) level of theory with charge = 0, multiplicity = 1. Fe-centroid distance 1.684 Å.

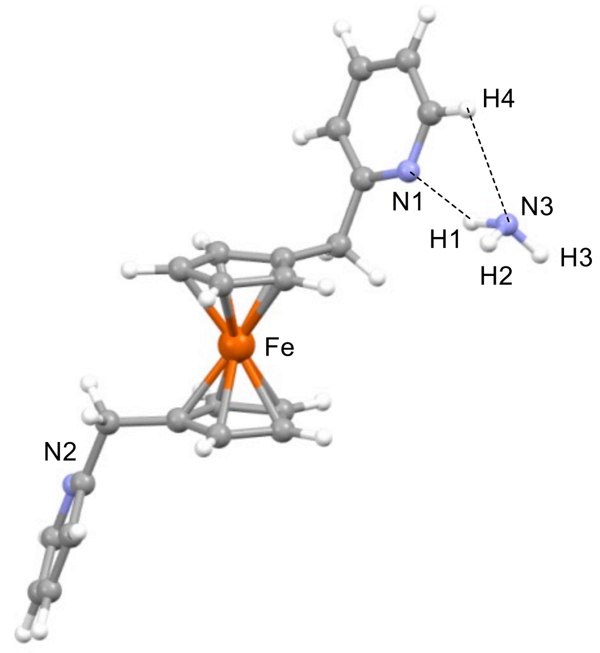

Figure S58. Geometry optimized structure of NH_3···**^{py2}Fc** at B3LYP/6-311+G(d,p) level of theory with charge = 0, multiplicity = 1. Fe-centroid distance 1.684 Å. Selected calculated bond distances (Å): N1–H1 2.251, N2–H4 2.850.

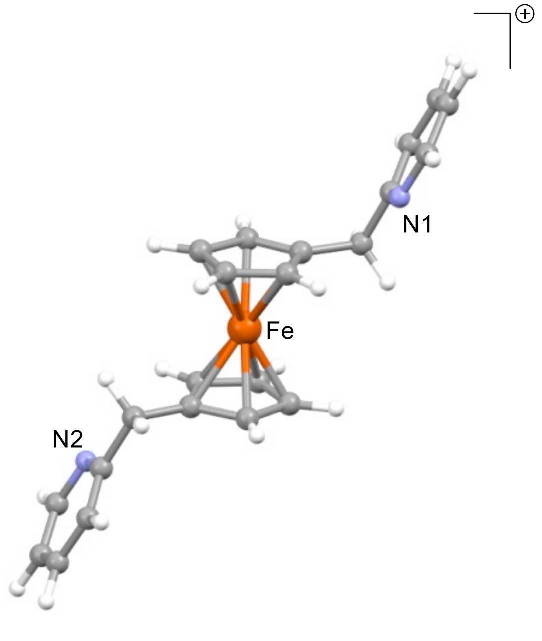

Figure S59. Geometry optimized structure of {**^{py2}Fc**}$^+$ at B3LYP/6-311+G(d,p) level of theory with charge = 1, multiplicity = 2. Fe-centroid distance 1.684 Å.

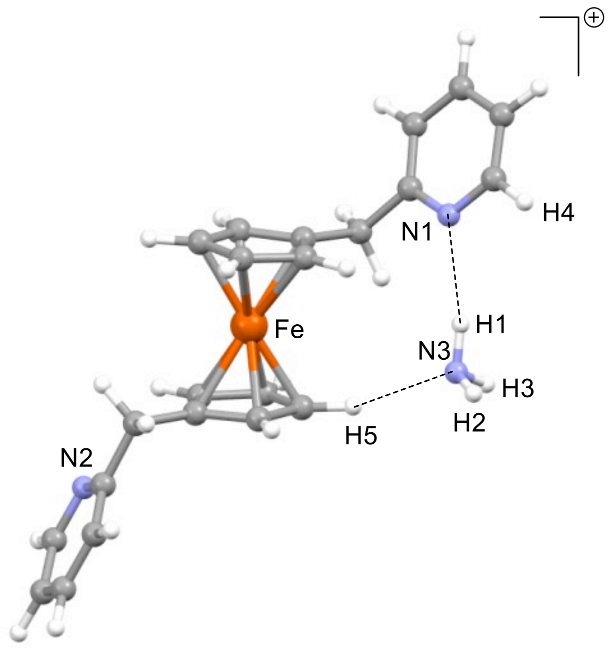

Figure S60. Geometry optimized structure of $\{NH_3{\cdots}^{\mathbf{py2}}\mathbf{Fc}\}^+$ at B3LYP/6-311+G(d,p) level of theory with charge = 1, multiplicity = 2. Fe-centroid distances 1.747 and 1.741 Å. Selected calculated bond distances (Å): N1–H1 2.327, N3–H5 2.331, Fe---N3 4.490.

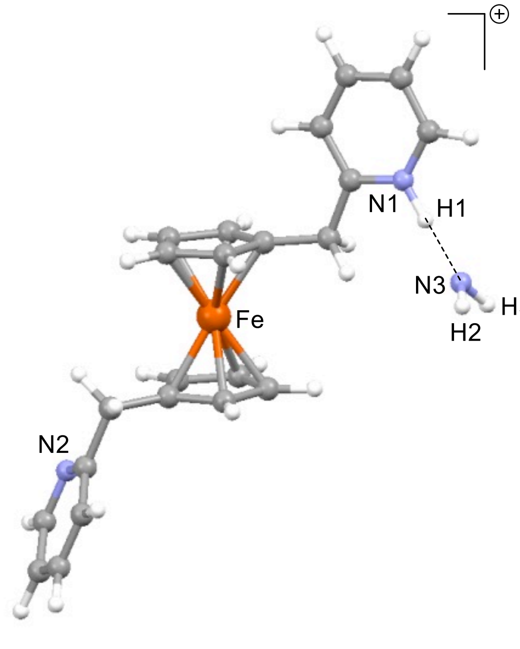

Figure S61. Geometry optimized structure of $\{NH_2{\cdots}H^{\mathbf{py2}}\mathbf{Fc}\}^+$ at B3LYP/6-311+G(d,p) level of theory with charge = 1, multiplicity = 2. Fe-centroid distance 1.698 Å. Selected calculated bond distances (Å): N1–H1 1.022, N3–H1 1.829, Fe---N3 6.086.

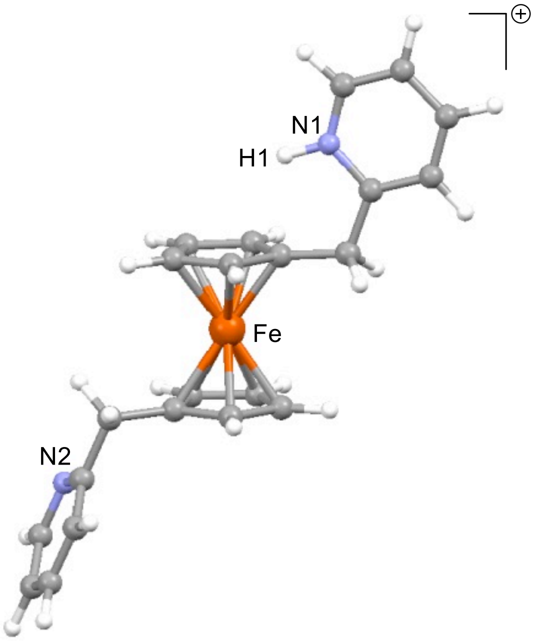

Figure S62. Geometry optimized structure of $\{H^{py2}\mathbf{Fc}\}^+$ at B3LYP/6-311+G(d,p) level of theory with charge = 1, multiplicity = 1. Fe-centroid distances 1.675 and 1.690 Å. Selected calculated bond distances (Å): N1–H1 1.023.

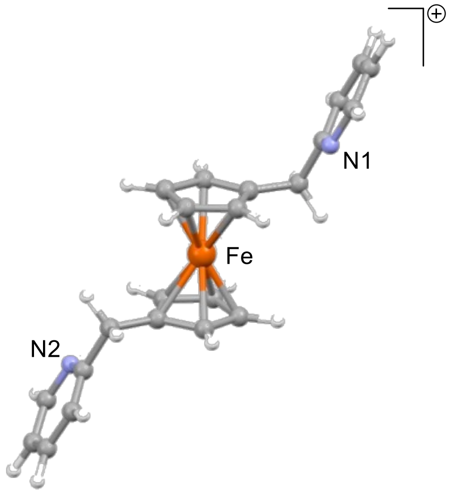

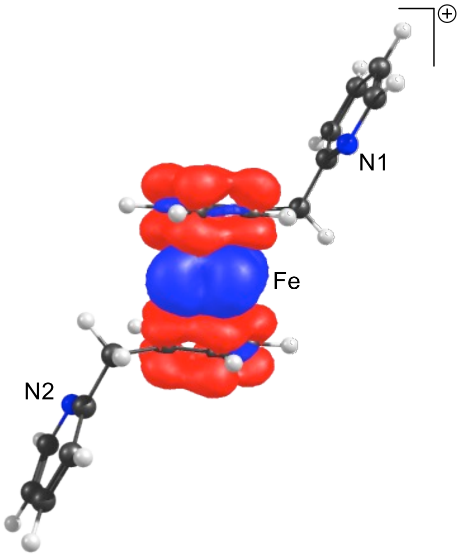

Figure S63. Spin density plot of $\{^{py2}\mathbf{Fc}\}^+$. Isosurface value = 0.0005. Spin densities: Fe 133%, N1 0.01%.

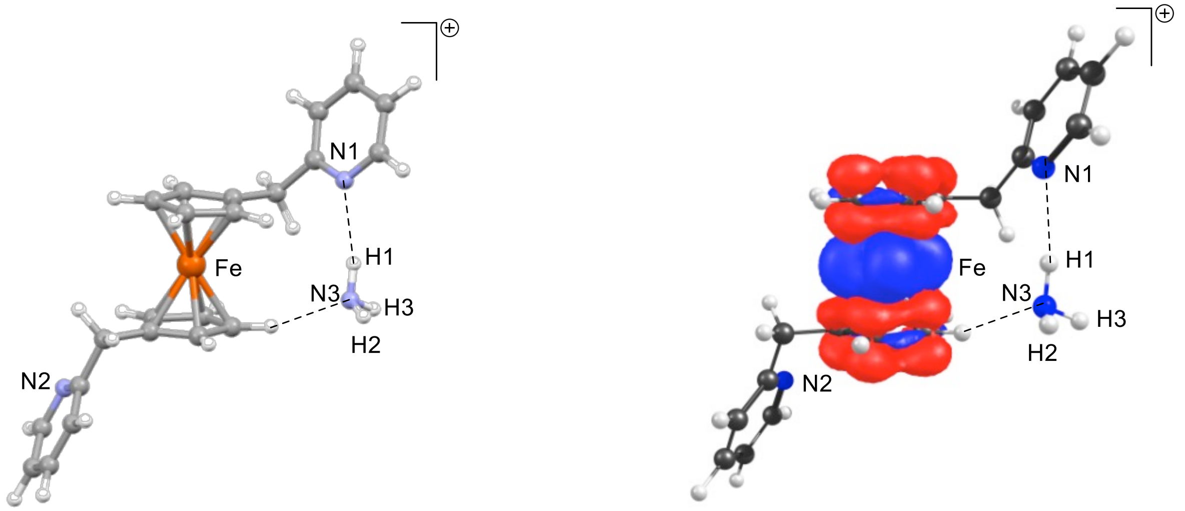

Figure S64. Spin density plot of $\{NH_3{}^{\cdots\mathbf{py2}}\mathbf{Fc}\}^+$. Isosurface value = 0.0005. Spin densities: Fe 133%, N1 -0.01%, N3 0.02%.

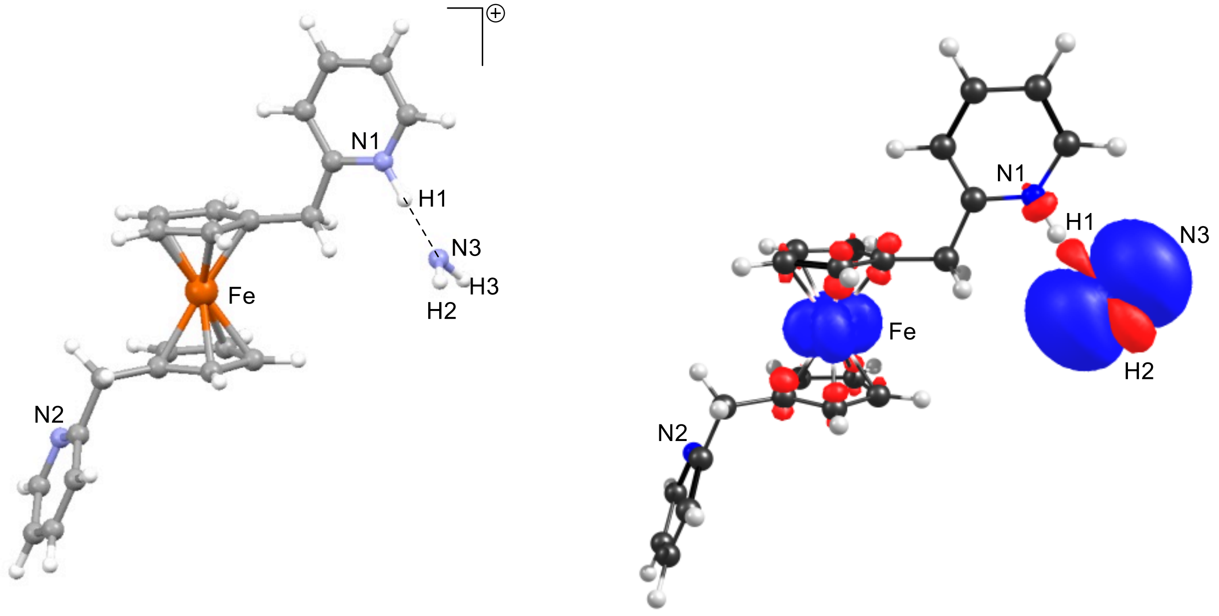

Figure S65. Spin density plot of $\{NH_2{}^{\cdots}H^{\mathbf{py2}}\mathbf{Fc}\}^+$. Isosurface value = 0.0005. Spin densities: Fe 38%, N3 81%.

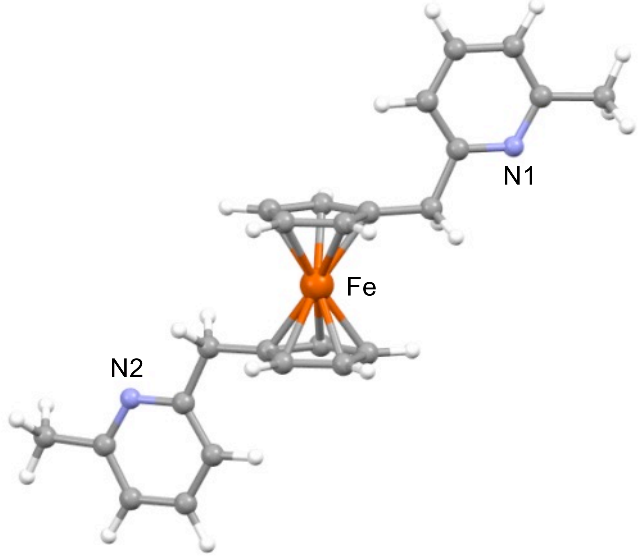

Figure S66. Geometry optimized structure of **$^{(Mepy)2}$Fc** (**2**) at B3LYP/6-311+G(d,p) level of theory with charge = 0, multiplicity = 1. Fe-centroid distance 1.683 Å.

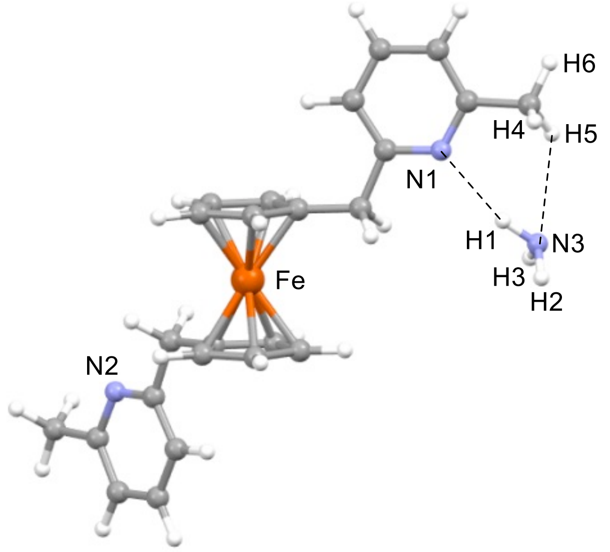

Figure S67. Geometry optimized structure of NH_3···**$^{(Mepy)2}$Fc** at B3LYP/6-311+G(d,p) level of theory with charge = 0, multiplicity = 1. Fe-centroid distance 1.683 Å. Selected calculated bond distances (Å): N1–H1 2.286, N3–H5 2.762.

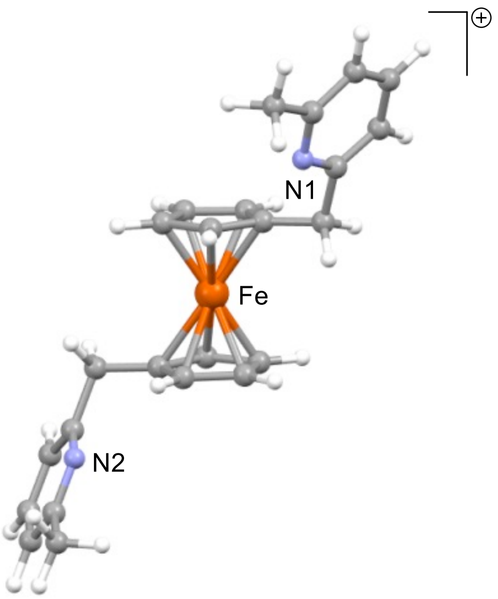

Figure S68. Geometry optimized structure of {$^{\mathbf{(Mepy)2}}$**Fc**}$^+$ at B3LYP/6-311+G(d,p) level of theory with charge = 1, multiplicity = 2. Fe-centroid distance 1.684 Å.

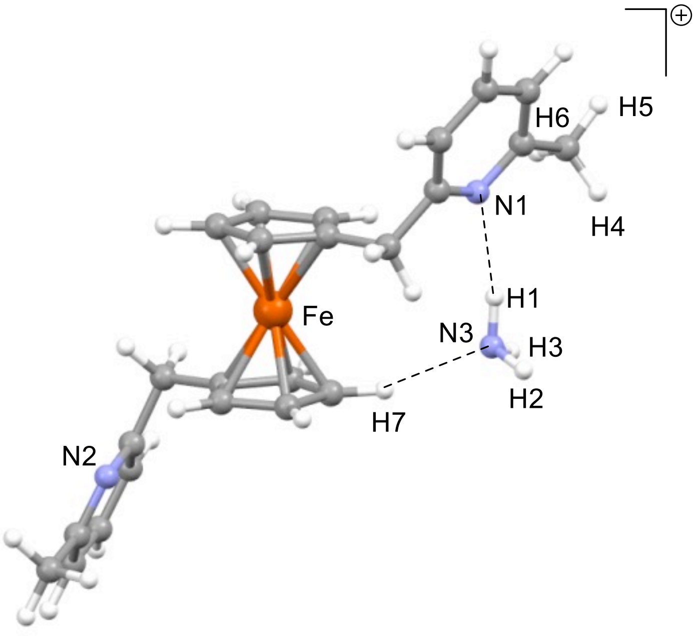

Figure S69. Geometry optimized structure of {$NH_3$$^{\cdots\mathbf{(Mepy)2}}$**Fc**}$^+$ at B3LYP/6-311+G(d,p) level of theory with charge = 1, multiplicity = 2. Fe-centroid distances 1.746 and 1.742 Å. Selected calculated bond distances (Å): N1–H1 2.352, N3–H7 2.349, Fe---N3 4.501.

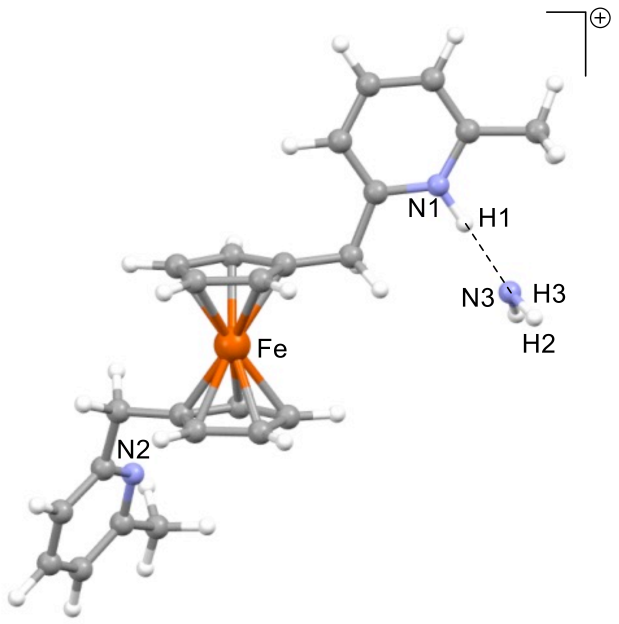

Figure S70. Geometry optimized structure of {NH_2···$H^{(Mepy)2}$**Fc**}$^+$ at B3LYP/6-311+G(d,p) level of theory with charge = 1, multiplicity = 2. Fe-centroid distance 1.689 and 1.695 Å. Selected calculated bond distances (Å): N1–H1 1.026, N3–H1 1.816, Fe---N3 6.131.

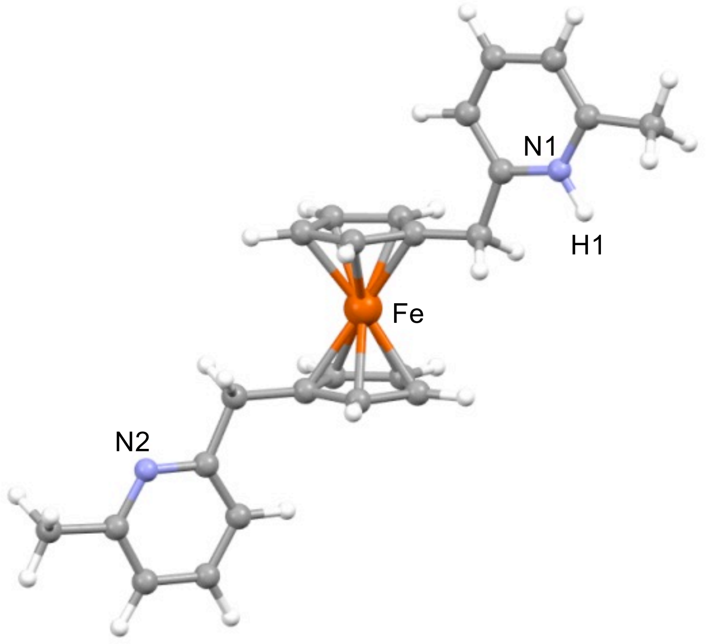

Figure S71. Geometry optimized structure of {$H^{(Mepy)2}$**Fc**}$^+$ at B3LYP/6-311+G(d,p) level of theory with charge = 1, multiplicity = 1. Fe-centroid distances 1.680 and 1.688 Å. Selected calculated bond distances (Å): N1–H1 1.017.

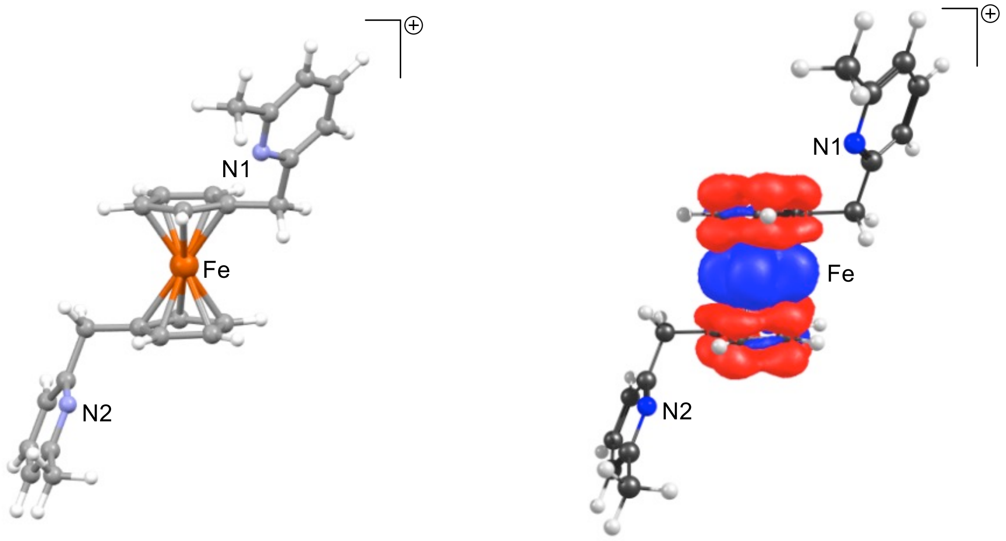

Figure S72. Spin density plot of $\{^{(\mathbf{Mepy})2}\mathbf{Fc}\}^{+}$. Isosurface value = 0.0005. Spin densities: Fe 134%, N1 0.03%.

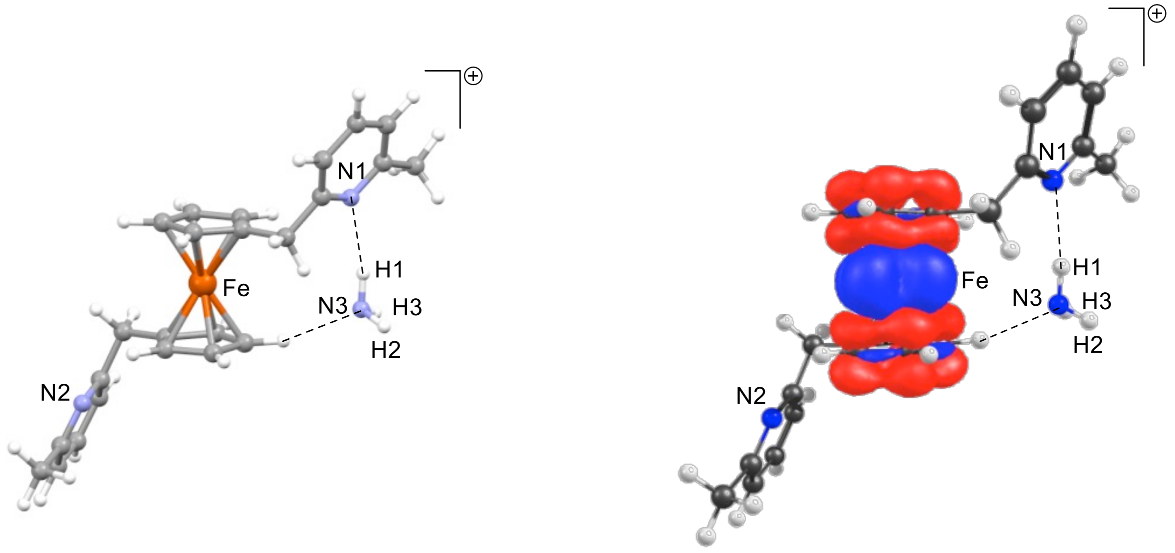

Figure S73. Spin density plot of $\{NH_3{}^{\cdots(\mathbf{Mepy})2}\mathbf{Fc}\}^{+}$. Isosurface value = 0.0005. Spin densities: Fe 133%, N1 0.0%, N3 0.02%.

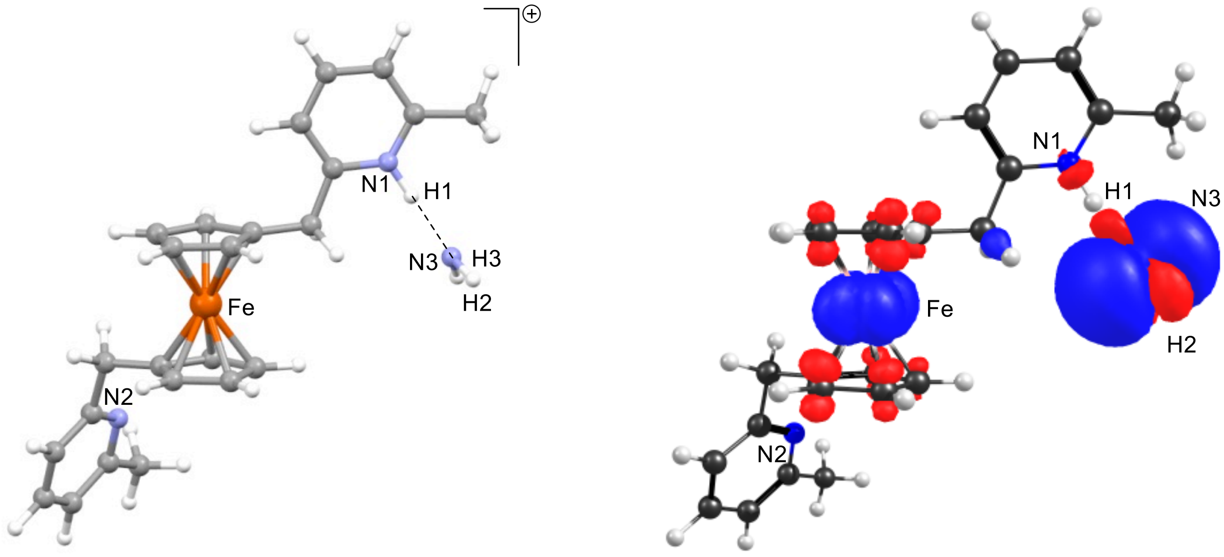

Figure S74. Spin density plot of $\{NH_2{\cdots}H^{\mathbf{(Mepy)2}}\mathbf{Fc}\}^{+}$. Isosurface value = 0.0005. Spin densities: Fe 21.6%, N3 93%.

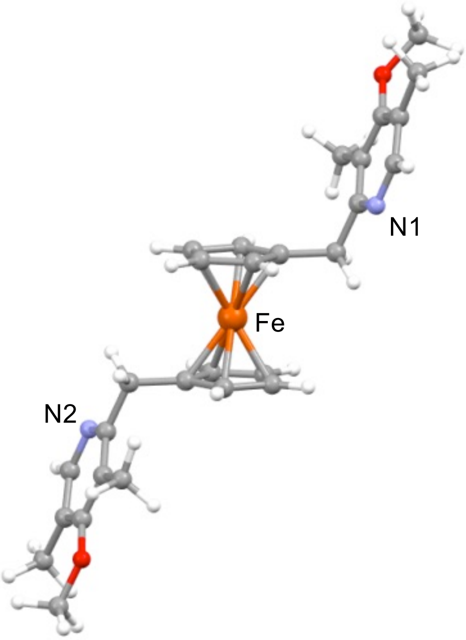

Figure S75. Geometry optimized structure of $^{\mathbf{(Me2OMepy)2}}$**Fc** (**3**) at B3LYP/6-311+G(d,p) level of theory with charge = 0, multiplicity = 1. Fe-centroid distance 1.687 Å.

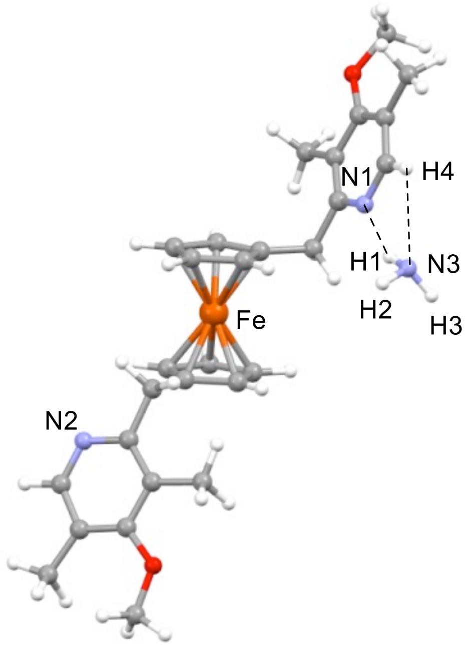

Figure S76. Geometry optimized structure of NH_3···**$^{(Me2OMepy)2}$Fc** at B3LYP/6-311+G(d,p) level of theory with charge = 0, multiplicity = 1. Fe-centroid distance 1.684 Å. Selected calculated bond distances (Å): N1–H1 2.225, N3–H4 2.897.

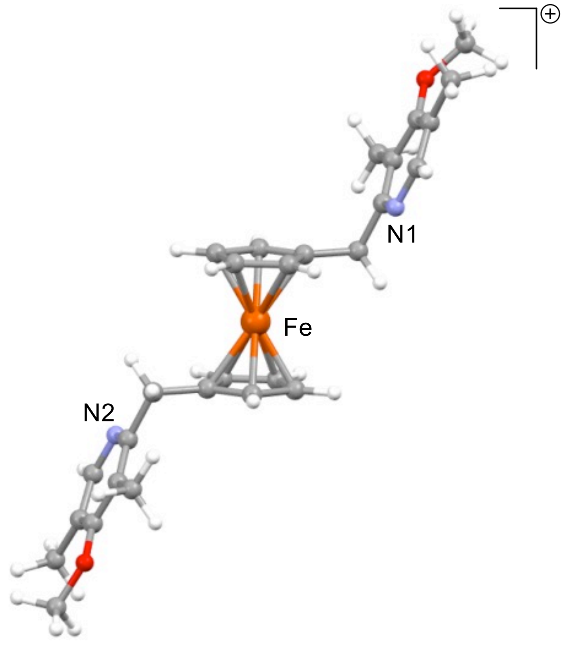

Figure S77. Geometry optimized structure of {**$^{(Me2OMepy)2}$Fc**}$^+$ at B3LYP/6-311+G(d,p) level of theory with charge = 1, multiplicity = 2. Fe-centroid distance 1.745 Å.

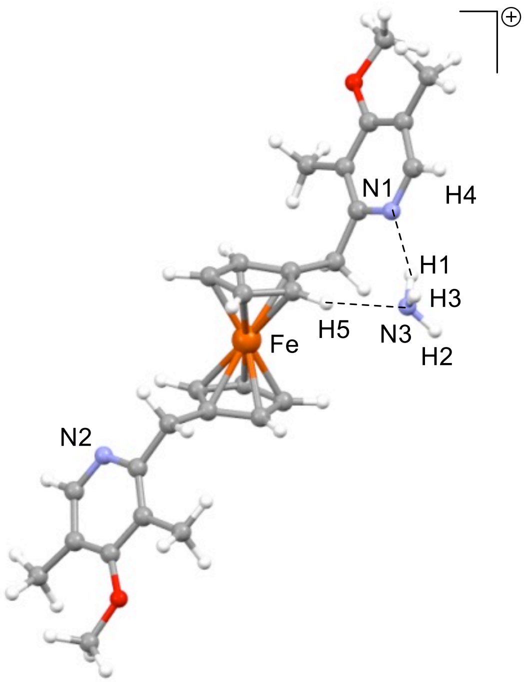

Figure S78. Geometry optimized structure of $\{NH_3{\cdots}^{(Me2OMepy)2}\mathbf{Fc}\}^+$ at B3LYP/6-311+G(d,p) level of theory with charge = 1, multiplicity = 2. Fe-centroid distances 1.742 and 1.745 Å. Selected calculated bond distances (Å): N1–H1 2.263, N3–H5 2.240, Fe---N3 4.932.

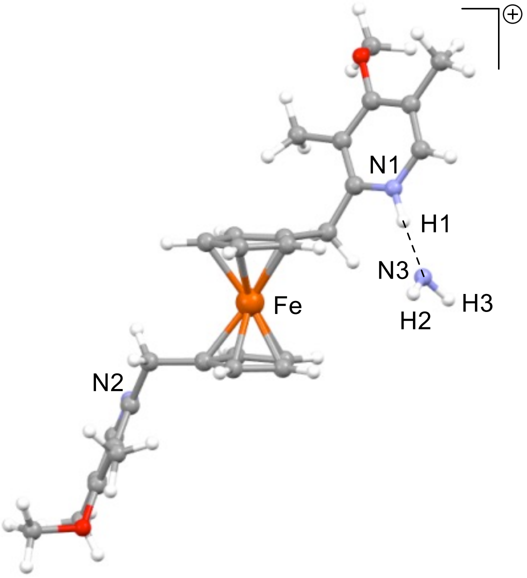

Figure S79. Geometry optimized structure of $\{NH_2{\cdots}H^{(Me2OMepy)2}\mathbf{Fc}\}^+$ at B3LYP/6-311+G(d,p) level of theory with charge = 1, multiplicity = 2. Fe-centroid distances 1.695 and 1.699 Å. Selected calculated bond distances (Å): N1–H1 1.020, N3–H1 1.777, Fe---N3 4.869.

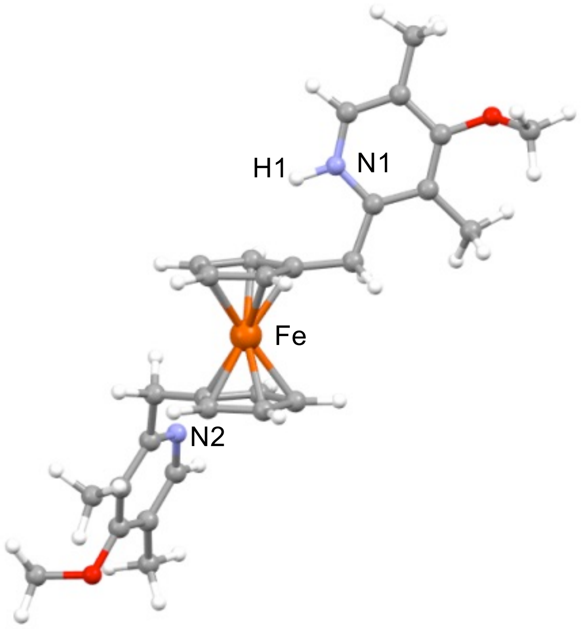

Figure S80. Geometry optimized structure of $\{H^{(Me2OMepy)2}Fc\}^+$ at B3LYP/6-311+G(d,p) level of theory with charge = 1, multiplicity = 1. Fe-centroid distances 1.675 and 1.689 Å. Selected calculated bond distances (Å): N1–H1 1.021.

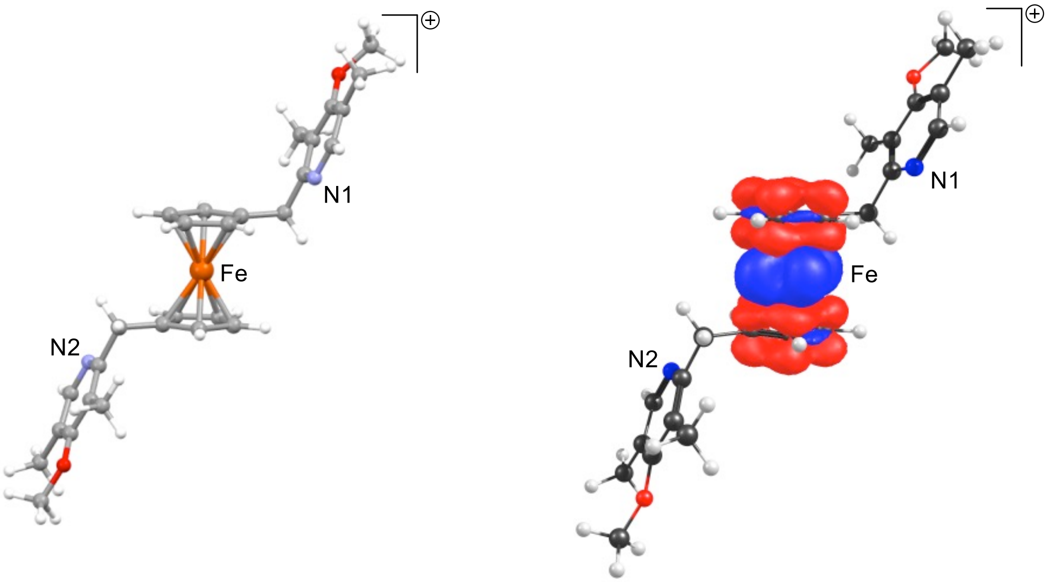

Figure S81. Spin density plot of $\{^{(Me2OMepy)2}Fc\}^+$. Isosurface value = 0.0005. Spin densities: Fe 135%, N1 0.0%.

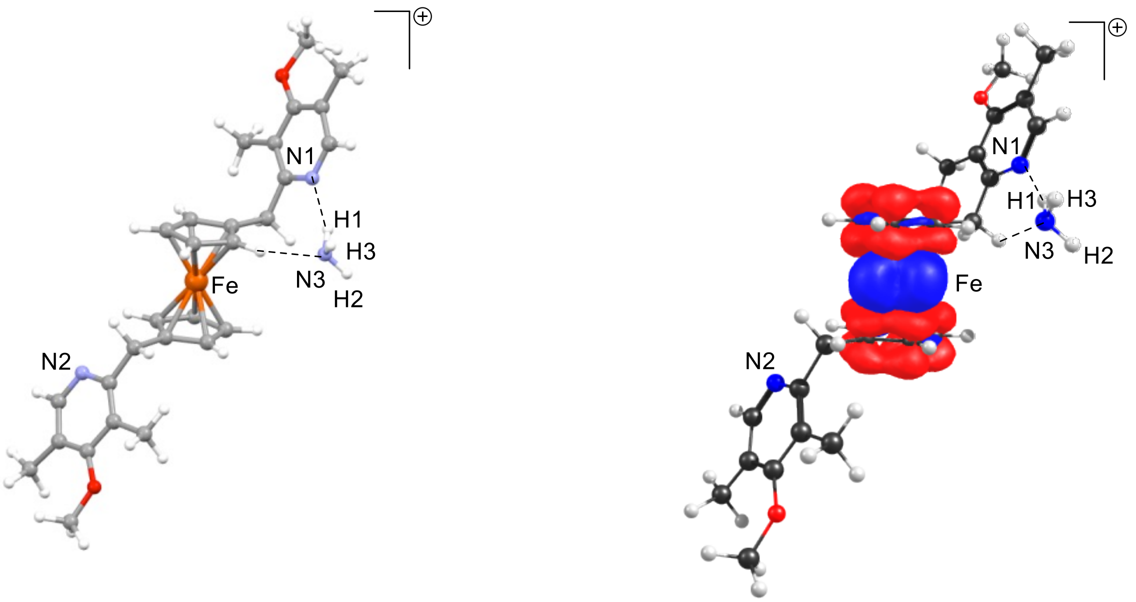

Figure S82. Spin density plot of {NH_3···$^{\mathbf{(Me2OMepy)2}}$**Fc**}$^+$. Isosurface value = 0.0005. Spin densities: Fe 135%, N1 -0.01%, N3 0.0%.

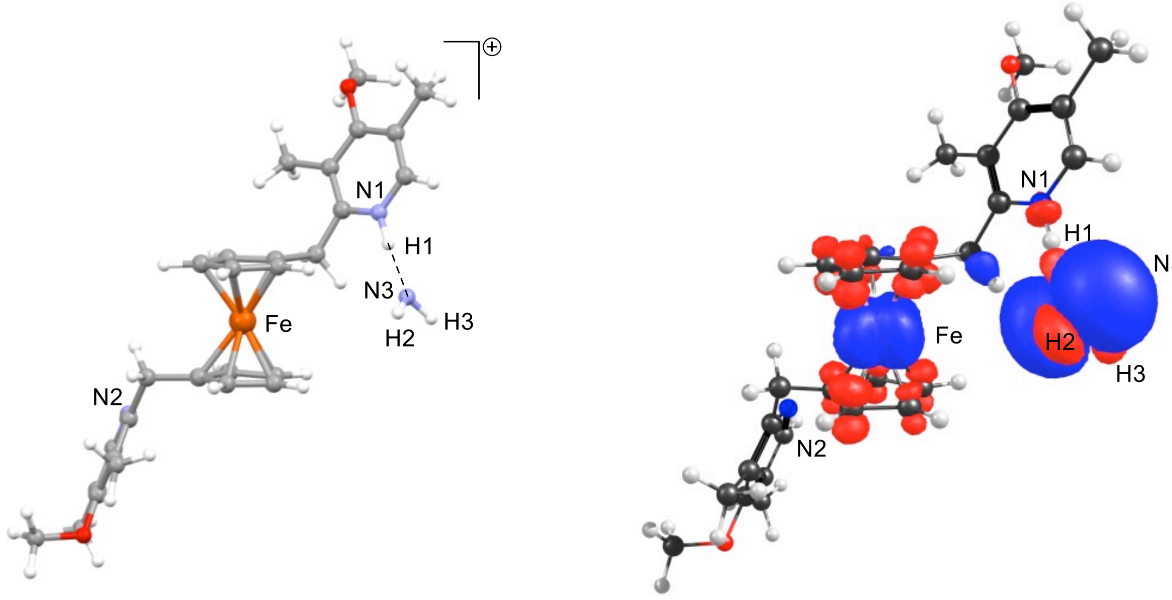

Figure S83. Spin density plot of {NH_2···H$^{\mathbf{(Me2OMepy)2}}$**Fc**}$^+$. Isosurface value = 0.0005. Spin densities: Fe 32%, N3 86%.

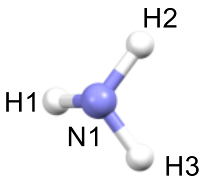

Figure S84. Geometry optimized structure of NH_3 at B3LYP/6-311+G(d,p) level of theory with charge = 0, multiplicity = 1. Selected calculated bond distances (Å): N1–H1 1.015.

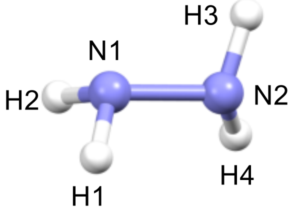

Figure S85. Geometry optimized structure of N_2H_4 at B3LYP/6-311+G(d,p) level of theory with charge = 0, multiplicity = 1. Selected calculated bond distances (Å): N1–H1 1.013, N1–H2 1.017, N1–N2 1.431.

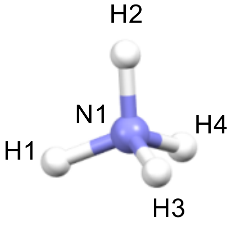

Figure S86. Geometry optimized structure of NH_4^+ at B3LYP/6-311+G(d,p) level of theory with charge = 1, multiplicity = 1. Selected calculated bond distances (Å): N1–H1 1.026.

16.2. Estimating Uphill Free Energies for PCET in Conversion of $\{H_2N\text{-}H^{\cdots py2\prime}Fc\}^+$ to $\{H_2N^{\cdots}H^{py2\prime}Fc\}^+$

From the analysis of the 1-electron oxidation of py$^{\cdots}$H-NH_2 adducts that result in pyridinium cations H-bonded to amidyl radicals $\{pyH^{\cdots}NH_2\}^{+/\bullet}$, the free energy of this transformation is approximately 15-20 kcal/mol uphill from the Fc/Fc^+ couple. Thus, we anticipated that the intramolecular electron transfer from the H-bonded ammonia-pyridine pendant to the oxidized ferrocenium center similarly would be uphill. Thus it is necessary to apply constraints to identify the structure that represents intramolecular PCET.

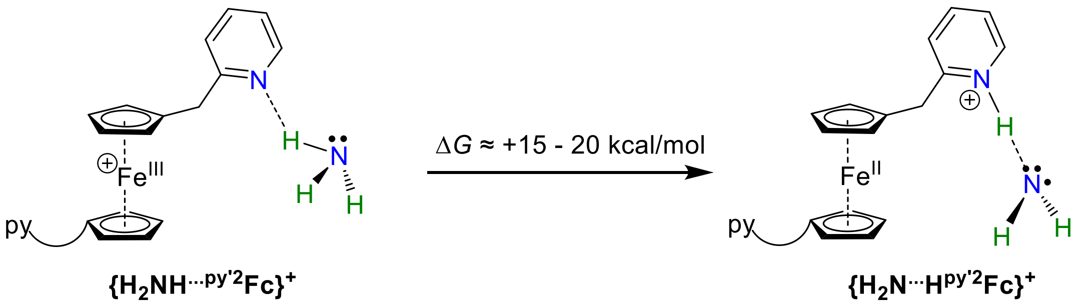

To provide a starting point for the ground state structures of $\{H_2N^{\cdots}H^{py'2}Fc\}^+$ species, we began with a relaxed energy scan starting from the $\{NH_3{}^{\cdots py'2}Fc\}^+$ complex. The process involved gradually increasing the N–H bond distance in NH_3 by 0.01 Å increments until the formation of the pyridinium and amide radical pair $\{H_2N^{\cdots}H^{py'2}Fc\}^+$ occurred.

To refine the lowest energy $\{H_2N^{\cdots}H^{py'2}Fc\}^+$ structure that results from PCET, we optimized a series of structures in which the pyridinium N-H distance was fixed at different distances. The range of pyridinium distances was generally 0.98 – 1.06 Å (Figures S87-S89) and was varied by 0.01 Å in this series of scans. Among this series of optimized structures that resulted by fixing the pyridinium N-H distance, the $\{H_2N^{\cdots}H^{py'2}Fc\}^+$ structure with the lowest electronic energy was used for free energy calculations and spin density plots.

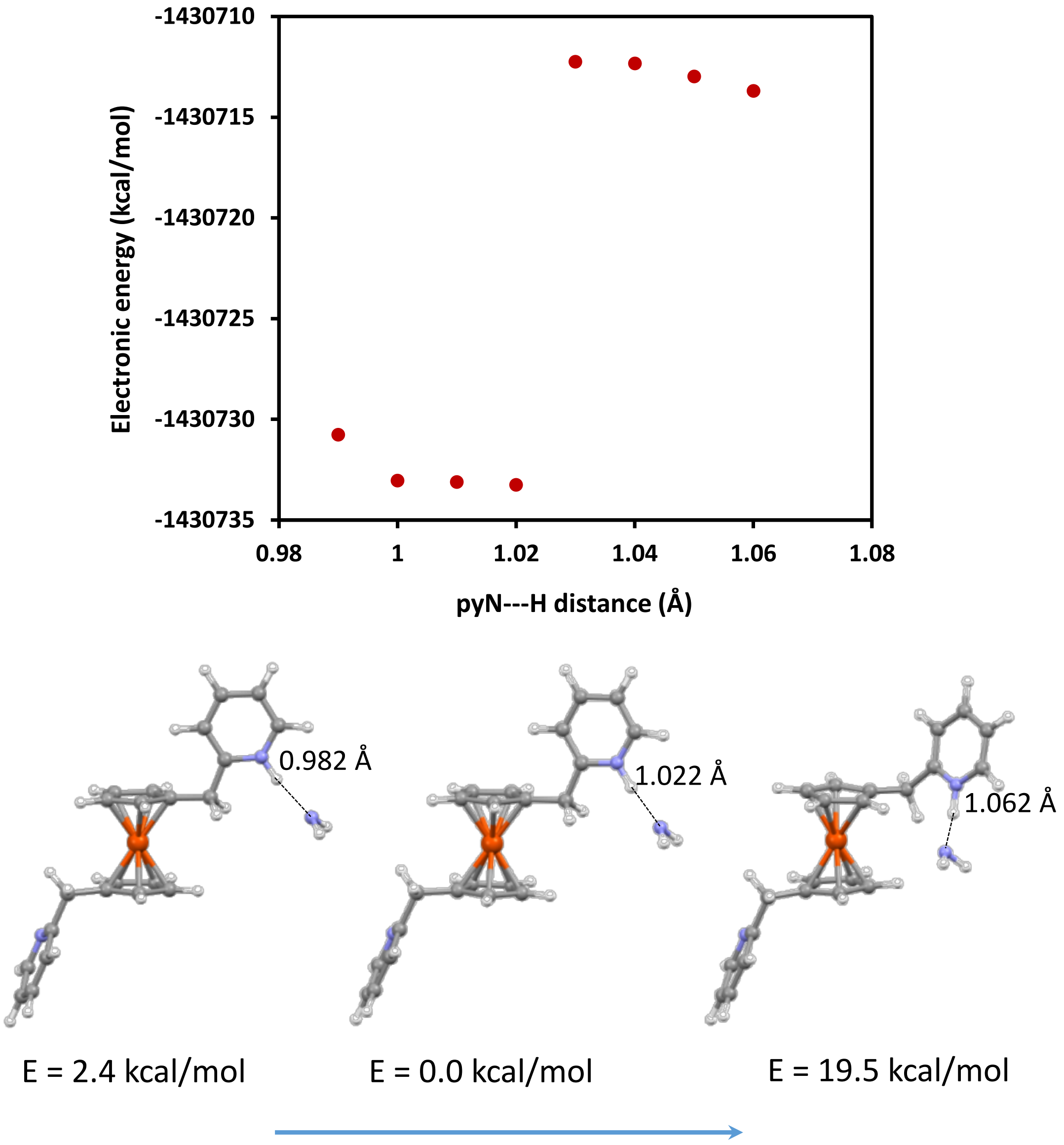

Figure S87. Electronic energy of optimized structures for $^{H2N\ldots Hpy'2}$**Fc**$^{+}$ based on catalyst **1** as the pyN–H distance is varied from 0.982 to 1.062 Å. The lowest energy structure from this scan has pyN–H = 1.022 Å.

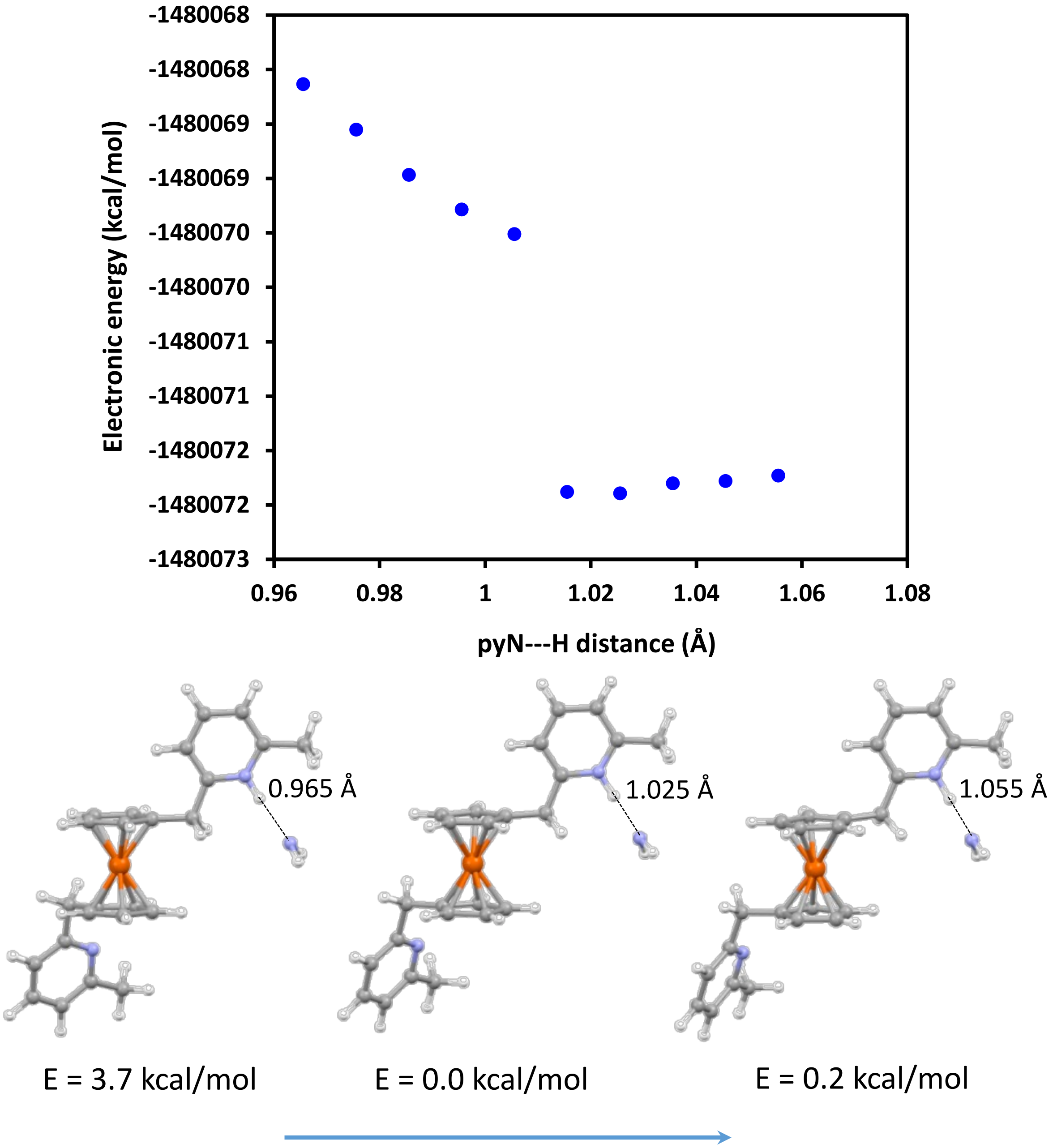

Figure S88. Electronic energy of optimized structures for $^{H2N\cdots Hpy'2}$**Fc**$^{+}$ based on catalyst **2** as the pyN–H distance is varied from 0.982 to 1.062 Å. The lowest energy structure from this scan has pyN–H = 1.022 Å.

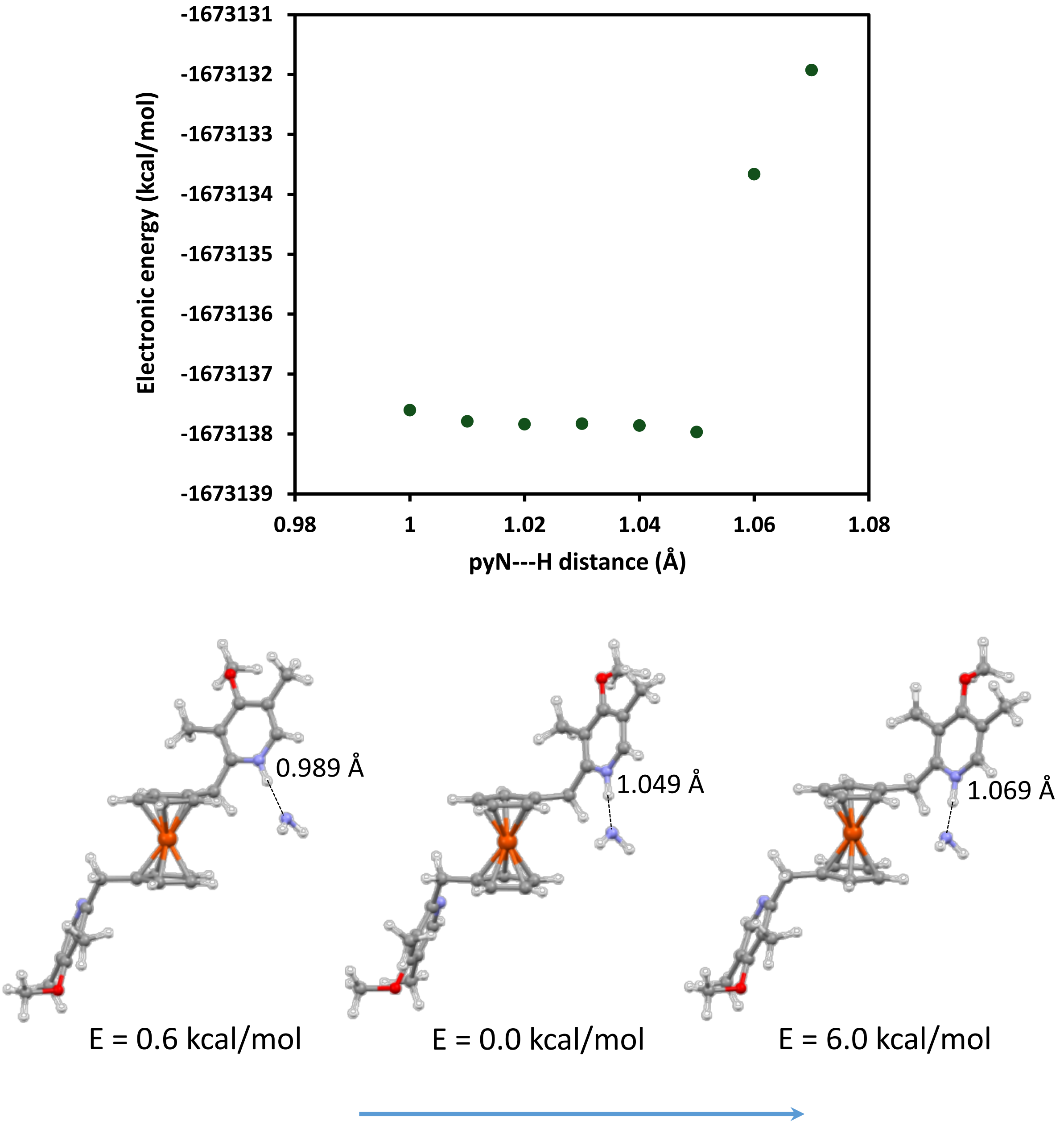

Figure S89. Electronic energy of optimized structures for $^{H2N...Hpy'2}$**Fc**$^{+}$ based on catalyst **3** as the pyN–H distance is varied from 0.989 to 1.069 Å. The lowest energy structure from this scan has pyN–H = 1.049 Å.

17. References

1. Kern, R. J., Tetrahydrofuran complexes of transition metal chlorides. *Journal of Inorganic and Nuclear Chemistry* **1962,** *24*, 1105-1109.
2. Gomberg, M., AN INSTANCE OF TRIVALENT CARBON: TRIPHENYLMETHYL. *J. Am. Chem. Soc.* **1900,** *22*, 757-771.
3. Liu, T.; Guo, M.; Orthaber, A.; Lomoth, R.; Lundberg, M.; Ott, S.; Hammarström, L., Accelerating proton-coupled electron transfer of metal hydrides in catalyst model reactions. *Nat. Chem.* **2018,** *10*, 881-887.
4. Ahmed, M. E.; Raghibi Boroujeni, M.; Ghosh, P.; Greene, C.; Kundu, S.; Bertke, J. A.; Warren, T. H., Electrocatalytic Ammonia Oxidation by a Low-Coordinate Copper Complex. *J. Am. Chem. Soc.* **2022,** *144*, 21136-21145.
5. Costentin, C.; Drouet, S.; Robert, M.; Savéant, J.-M., Turnover Numbers, Turnover Frequencies, and Overpotential in Molecular Catalysis of Electrochemical Reactions. Cyclic Voltammetry and Preparative-Scale Electrolysis. *J. Am. Chem. Soc.* **2012,** *134*, 11235-11242.
6. Habibzadeh, F.; Miller Susanne, L.; Hamann Thomas, W.; Smith Milton, R., Homogeneous electrocatalytic oxidation of ammonia to N2 under mild conditions. *Proc. Natl. Acad. Sci. U.S.A.* **2019,** *116*, 2849-2853.
7. Spek, A. L., PLATON SQUEEZE: a tool for the calculation of the disordered solvent contribution to the calculated structure factors. *Acta Crystallogr C Struct Chem* **2015,** *71*, 9-18.

7. Gaussian 16, Revision A.03, M. J. Frisch, G. W. Trucks, H. B. Schlegel, G. E. Scuseria, M. A. Robb, J. R. Cheeseman, G. Scalmani, V. Barone, G. A. Petersson, H. Nakatsuji, X. Li, M. Caricato, A. V. Marenich, J. Bloino, B. G. Janesko, R. Gomperts, B. Mennucci, H. P. Hratchian, J. V. Ortiz, A. F. Izmaylov, J. L. Sonnenberg, D. Williams-Young, F. Ding, F. Lipparini, F. Egidi, J. Goings, B. Peng, A. Petrone, T. Henderson, D. Ranasinghe, V. G. Zakrzewski, J. Gao, N. Rega, G. Zheng, W. Liang, M. Hada, M. Ehara, K. Toyota, R. Fukuda, J. Hasegawa, M. Ishida, T. Nakajima, Y. Honda, O. Kitao, H. Nakai, T. Vreven, K. Throssell, J. A. Montgomery, Jr., J. E. Peralta, F. Ogliaro, M. J. Bearpark, J. J. Heyd, E. N. Brothers, K. N. Kudin, V. N. Staroverov, T. A. Keith, R. Kobayashi, J. Normand, K. Raghavachari, A. P. Rendell, J. C. Burant, S. S. Iyengar, J. Tomasi, M. Cossi, J. M. Millam, M. Klene, C. Adamo, R. Cammi, J. W. Ochterski, R. L. Martin, K. Morokuma, O. Farkas, J. B. Foresman, and D. J. Fox, Gaussian, Inc., Wallingford CT, **2016**.
8. (a) **ChemCraft 1.8** [http://www.chemcraftprog.com], (b) Macrae, C. F.; Sovago, I.; Cottrell, S. J.; Galek, P. T. A.; McCabe, P.; Pidcock, E.; Platings, M.; Shields, G. P.; Stevens, J. S.; Towler, M.; Wood P. A. Mercury 4.0: from Visualization to Analysis, Design and Prediction. *J. Appl. Cryst.* **2020**, *53*, 226-235.
9. (a) A. D. Becke, Density-Functional Thermochemistry. III. The Role of ExactE. *J. Chem. Phys.* **1993**, *98*, 5648-5652. (b) C. Lee, W. Yang, R. G. Parr, Development of the Colle-Salvetti Correlation-Energy Formula into a Functional of the Electron Density. *Phys. Rev. B* **1988**, *37*, 785-789.
10. Wachters, A. J. H. Gaussian Basis Set for Molecular Wavefunctions Containing Third-Row Atoms. *J. Chem. Phys.* **1970**, *52*, 1033-1036.
11. Hay, P. J. Gaussian basis sets for molecular calculations. The representation of 3d orbitals in Transition-metal atoms. *J. Chem. Phys.* **1977**, *66*, 4377-4384.
12. Raghavachari, K.; Trucks, G. W. Highly Correlated Systems: Excitation Energies of First Row Transition Metals Sc-Cu. *J. Chem. Phys.* **1989**, *91*, 1062-1065.
13. Binning Jr., R. C.; Curtiss, L. A. Compact Contracted Basis Sets for Third-Row Atoms: Ga–Kr. *J. Comp. Chem.* **1990**, *11*, 1206-1216.

14. McGrath, M. P.; Radom, L. Extension of Gaussian-1 (G1) Theory to Bromine-Containing Molecules. *J. Chem. Phys.* **1991**, *94*, 511-516.
15. Curtiss, L. A.; McGrath, M. P.; Blaudeau, J.-P.; Davis, N. E.; Binning Jr., R. C.; Radom, L. Extension of Gaussian-2 Theory to Molecules Containing Third-Row Atoms Ga-Kr. *J. Chem. Phys.* **1995**, *103*, 6104-6113.
16. Grimme, S.; Ehrlich, S.; Goerigk, L. Effect of the Damping Function in Dispersion Corrected Density Functional Theory. *J. Comp. Chem.* **2011**, *32*, 1456-1465.
17. Marenich, A. V.; Cramer, C. J.; Truhlar, D. G. Universal Solvation Model Based on Solute Electron Density and on a Continuum Model of the Solvent Defined by the Bulk Dielectric Constant and Atomic Surface Tensions. *J. Phys. Chem. B* **2009**, *113*, 6378-6396.
18. Hariharan, P. C.; Pople, J. A. The Influence of Polarization Functions on Molecular Orbital Hydrogenation Energies. *Theoret. Chimica. Acta*. **1973**, *28*, 213-222.
19. Francl, M. M.; Petro, W. J.; Hehre, W. J.; Binkley, J. S.; Gordon, M. S.; DeFrees, D. J.; Pople, J. A. Self-Consistent Molecular Orbital Methods. XXIII. A Polarization-type Basis Set for Second-Row Elements. *J. Chem. Phys.* **1982**, *77*, 3654-3665.
20. Rassolov, V.; Pople, J. A.; Ratner, M.; Windus, T. L. 6-31G* Basis Set for Atoms K Through Zn. *J. Chem. Phys.* **1998**, *109*, 1223-1229.
21. McLean, A. D.; Chandler, G. S. Contracted Gaussian Basis Sets for Molecular Calculations. I. Second Row Atoms, Z=11-18. *J. Chem. Phys.* **1980**, **72**, 5639-5648.
22. Raghavachari, K.; Binkley, J. S.; Seeger, R.; Pople, J. A. Self-Consistent Molecular Orbital Methods. XX. A Basis Set for Correlated Wave Functions. *J. Chem. Phys.* **1980**, *72*, 650-654.
23. Blaudeau, J.-P.; McGrath, M. P.; Curtiss, L. A.; Radom, L. Extension of Gaussian-2 (G2) Theory to Molecules Containing Third-Row Atoms K and Ca. *J. Chem. Phys.* **1997**, 107, 5016-5021.
